# Cation Effects on the Adsorbed Intermediates of CO_2_ Electroreduction Are Systematic and Predictable

**DOI:** 10.1021/acscatal.4c00727

**Published:** 2024-05-23

**Authors:** Elizabeth Sargeant, Paramaconi Rodriguez, Federico Calle-Vallejo

**Affiliations:** † School of Chemistry, University of Birmingham, Edgbaston, Birmingham B15 2TT, United Kingdom; ‡ Department of Materials Science and Chemical Physics & Institute of Theoretical and Computational Chemistry (IQTC), University of Barcelona, Barcelona 08028, Spain; § Centre for Cooperative Research on Alternative Energies (CIC energiGUNE), Basque Research and Technology Alliance (BRTA), Alava Technology Park, Vitoria-Gasteiz 01510, Spain; ∥ IKERBASQUE, Basque Foundation for Science, Plaza de Euskadi 5, Bilbao 48009, Spain; ⊥ Nano-Bio Spectroscopy Group and European Theoretical Spectroscopy Facility (ETSF), Department of Advanced Materials and Polymers: Physics, Chemistry and Technology, University of the Basque Country UPV/EHU, Avenida Tolosa 72, San Sebastian 20018, Spain

**Keywords:** cation effects, adsorbate-solvent interactions, adsorbate-electrolyte interactions, CO_2_ electroreduction, scaling relations

## Abstract

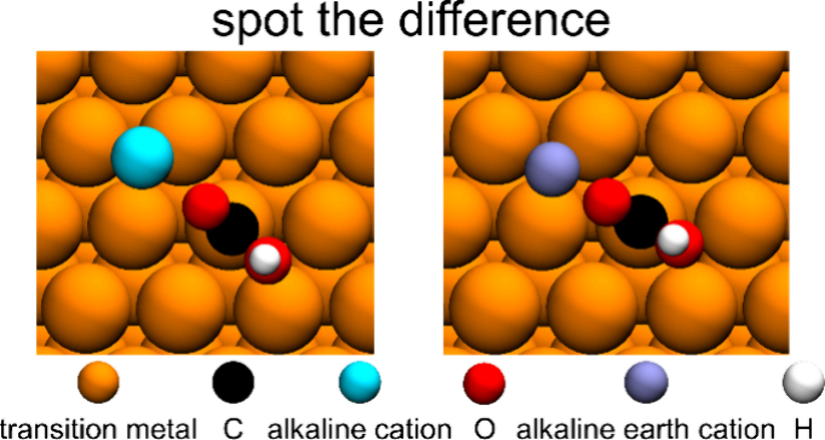

The electrode–electrolyte interface, and in particular the nature of the cation, has considerable effects on the activity and product selectivity of the electrochemical reduction of CO_2_. Therefore, to improve the electrocatalysis of this challenging reaction, it is paramount to ascertain whether cation effects on adsorbed intermediates are systematic. Here, DFT calculations are used to show that the effects of K^+^, Na^+^, and Mg^2+^, on single carbon CO_2_ reduction intermediates can either be stabilizing or destabilizing depending on the metal and the adsorbate. Because systematic trends are observed, cation effects can be accurately predicted in simple terms for a wide variety of metals, cations and adsorbed species. These results are then applied to the reduction of CO_2_ to CO on four different catalytic surfaces (Au, Ag, Cu, Pd) and activation of weak-binding metals is consistently observed by virtue of the stabilization of the key intermediate *COOH.

## 1 Introduction

In the transition toward carbon neutrality, many large CO_2_ emitting industries will face difficulties to moving swiftly and smoothly to utilization of alternative sources of energy. Therefore, effective and selective electrochemical CO_2_ reduction reaction (CO_2_RR) electrolyzers will be an important technology for transforming and valorising CO_2_ emissions.^1,2^ Despite its great potential, the industrial application of CO_2_RR electrolyzers is yet to be realized due in part to the large number of possible products. On one hand, current efforts focus on the development of new catalysts and reactor design.^3−6^ On the other, more attention is paid to understanding the effect of the local reaction environment i.e. the liquid-electrode interface.^7−10^

Density functional theory (DFT) calculations have been used in the past to develop a deeper understanding of experimental results. For example, on Cu(100) at low overpotentials, mainly C_2_ products are observed in experiments, and the formation of ethylene is shown to be independent of pH.^11,12^ Results from DFT calculations showed that at low overpotential, the formation of an adsorbed C_2_O_2_ intermediate (which was observed in its hydrogenated form in FTIR experiments), via a decoupled proton–electron transfer step, is the rate-determining step for the reduction of CO to ethylene on this surface.^13,14^ Further calculations proposed that at high overpotential, the reaction mechanism changes and the C_2_ intermediate is formed from the coupling of *CO and *CHO^15^ or the dimerization of *CH_2_. The protonation of *CO forms either *CHO or *COH and is considered one of the potential limiting steps toward C_1_ species.^16−19^ DFT screening of various facets of transition metals was able to show that *COH and *CHO are stabilized differently by various surfaces and so different strategies are required to stabilize each intermediate.^20,21^ In addition, the selectivity between CO and HCOOH is intricate and has been postulated to depend on a number of factors.^22,23^ All this highlights the difficulty in optimizing the CO_2_RR since there may be no single approach which will stabilize all intermediates appropriately, which is aggravated by scaling relations between adsorbed intermediates.^88^

Scaling relations are the basis of the most widely used method of screening many surfaces for catalytic purposes.^24,25^ The adsorption energy of similar intermediates correlates in a linear fashion, for example, *OH and *OOH which are key intermediates in oxygen catalysis,^26−28^ and C, S, N and O with their respective hydrogenated forms and other species.^24,29,30^ These scaling relations allow for the elaboration of Sabatier-type volcano plots where the most active catalysts sit at the apex. However, scaling relations are thought to cause intrinsic limitations to the minimization of the overpotential for reactions in which more than two electrons are transferred due to the constant separation of energy between intermediates that scale together with a unity slope.^27^ For CO_2_RR and akin reactions, there has been progress in detecting^17,21^ and understanding how to “break” these scaling relations^88,31−33^ but also criticism that there is insufficient consideration given to solvent effects and structural sensitivity.^20,34−36^

There are multiple ways to include solvent and/or electrolyte effects to try to improve the predictions of DFT calculations.^37^ There are two commonly used methods to mimic the effect of the surrounding solution. Implicit solvent methods treat the surrounding liquid as a continuous media and generally rely on its dielectric constant.^38−41^ These methods are computationally inexpensive but, because they often do not account for local and directional interactions such as hydrogen bonding between solvent or electrolyte molecules and adsorbates, they can be inaccurate.^42−45^ Explicit solvent models include water molecules in the calculations and, although these models have a greater accuracy, they are highly demanding in computational terms.^43^ An affordable approach is “*micro-solvation*”, in which a few explicit solvent and/or electrolyte species are included around the adsorbate. In the case of CO_2_RR and other reactions with small adsorbed intermediates, an iterative microsolvation model has been proposed that provides a good approximation to experimental catalytic activity results.^45−47^

The composition of the electrolyte can have many effects on the selectivity and the activity of the CO_2_RR in aqueous^48,49^ and non-aqueous electrolytes.^7,50^ Previous works studied the electrochemical conversion of CO_2_ in concentrated Mg(ClO_4_)_2_ and NaOH brines at subfreezing temperatures for applications on Mars and observed drastic changes in the selectivity with respect to experiments at room temperature. While the changes were mainly attributed to the temperature effect, the influence of the identity and charge of the cation, as well as the difference in hydration layers cannot be disregarded.^51^ In fact, it has been observed that the size of the cation can tune the selectivity of the reaction in aqueous electrolytes. Previous reports have shown that on Ag electrodes, larger cations promoted CO production over H_2_ production^52^ and increased the current density due to formation of CO and HCOO^–^.^53,54^ On Cu electrodes, *K and *I modify the adsorption energies of *CO and its first hydrogenation products,^55^ and increasing cation size also favors CO_2_ reduction over H_2_ evolution as well as promoting C_2_ products rather than C_1_.^56^ DFT calculations have provided an explanation by showing that larger cations provide greater stabilization to C_2_ intermediates.^57^ However, the reason for this stabilization is a subject of much debate. Hori and co-workers^56,58^ hypothesized that the cations adsorbed to the electrode surface changing the potential profile of the electric double layer. In this regard, smaller cations such as Li^+^ are strongly solvated and not able to adsorb to the surface, whereas larger cations such as Cs^+^, which are weakly solvated, can adsorb to the surface more easily and have a greater effect on the potential. In more recent studies implementing attenuated total reflection surface-enhanced infrared spectroscopy (ATR-SEIRAS), Ayemoba and Cuesta^59^ showed that larger cations are more effective at buffering the pH since the polarization of water in the solvation shell is greater, closer to the electrode surface. Such an increased buffering capability lowers the pH near the electrode surface, favoring an increase of the CO_2_ solubility.^60^ Another suggested interpretation for the change in selectivity and reactivity is associated with the stabilizing electrostatic interactions between the reaction intermediate and the cation at the solid–liquid interface.^53,57,61−63^

In perspective, the afore cited works showed various separate cases in which cation effects were present but did not ascertain whether and how cation effects are systematic. Herein, DFT calculations incorporating explicit water molecules and cations in the vicinity of the adsorbed species on metal electrodes are used to investigate the trends in stabilization of key CO_2_RR intermediates by metal cations. First, we show by means of scaling relations the qualitative and quantitative variations in the way cations affect the intermediates of CO_2_RR. Second, by virtue of the systematic trends, we illustrate how it is possible to predict cation effects in various ways. Finally, we show the activation of otherwise inactive sites by cations during CO_2_ reduction to CO via *COOH stabilization on several experimentally relevant electrodes.

## 2 Methods

All calculations were performed using the Vienna *ab initio* simulation package^64^ using the PBE exchange-correlation functional^65^ and the projector augmented-wave (PAW) method^66^ to describe the ion-electron interactions. Previous works showed that PBE and RPBE^67^ cannot simultaneously predict with reasonable accuracy the reaction energy and onset potentials of CO_2_RR to CO on metals.^68^ While errors in the reaction energy are similar (∼0.4 eV), errors in the onset potentials are larger for RPBE. Gas-phase corrections help both functionals match the experimental reaction energy and predict onset potentials close to experiments. Hence, the choice of PBE or RPBE is facultative when gas-phase corrections are used. Extensive analyses with more reactions, functionals, and adsorbates can be found in the literature that confirm our assessment.^89^ Geometry optimization was carried out using the conjugate gradient method with a plane wave cut-off of 450 eV. For calculations of slabs with adsorbates, relaxation was stopped when the maximal residual forces on all atoms was less than 0.05 eV/Å. The Methfessel–Paxton method^69^ was used to sample the Brillouin zone with an electronic temperature of 0.2 eV and all energies were then extrapolated to 0 K. The vertical distance between slabs was greater than 12 Å and dipole corrections were added to avoid spurious electrostatic interactions between vertically stacked images.

The gas-phase energies of species (*E*_*A*(g)_, where *A*(g) is C, CH, CH_2_, CH_3_, CH_4_, CO, COOH, COH, CHO) were used as a reference for the corresponding adsorption energies: Δ*E*_*A*_ = E_*A_ – E_*_ – *E*_*A*(g)_, with E_*_: energy of the clean surface, E_*A_: energy of the surface with the adsorbate *A*. In presence of a cation: Δ*E*_[*A*+cat]_ = *E*_[*A+*cat]_ – E_*cat_ – *E*_*A*(g)_, where E_*cat_ is the energy of the surface with the cation (cat: K, Na, Mg), and the brackets indicate co-adsorption in close proximity. The values of *E*_*A*(g)_ were either taken from the literature or calculated spin-unrestricted and using Gaussian smearing with an electronic temperature of 0.001 eV and again, all energies were extrapolated to 0 K.^21^ For gas-phase calculations a 15 Å box was used and the Brillouin zone was sampled using a (1 × 1 × 1) Monkhorst–Pack mesh.^70^

### 2.1 Reaction Energies

For the reduction of CO_2_ to CO, the reaction energy, Δ*G*, in vacuum was calculated using eq 1.
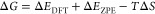
1where Δ*E*_DFT_ is the calculated DFT reaction energy, Δ*E*_ZPE_ is the zero-point energy change, *T* is the absolute temperature (set to 298.15 K), and Δ*S* is the entropic contribution. Δ*E*_ZPE_ was calculated with DFT using the vibrational frequencies obtained using the harmonic oscillator approximation. For gas and liquid molecules, Δ*S* was taken from standard entropies reported in the literature.^71^ A liquid-phase correction was applied to Δ*S* for water.^13,71,72^ For adsorbed intermediates, Δ*S* includes only the vibrational contribution. Heat capacity effects were not included since their effects are negligible on formation energies at 298.15 K.^73^ To account for the errors intrinsic to DFT calculation of gas phase energies, for CO_2_ and CO specifically, semiempirical corrections have been applied.^68^ When water solvation or cationic corrections are applied, it is assumed that any changes in Δ*E*_ZPE_ and Δ*S* are negligible.^45^ This was verified for the co-adsorption of *K and *CO on Ag(111) where Δ*E*_ZPE_ and Δ*S* were calculated and applied and the difference was shown to be less than 0.05 eV.

### 2.2 Effect of Water Solvation on CO_2_ Reduction to CO

When investigating the effect of solvation by water on the electroreduction of CO_2_ to CO, (3 × 3) slabs were used of the (111) surface. Four layers of atoms were used where the bottom 2 layers were fixed at the optimized bulk distance. Again, a Monkhorst–Pack mesh was used to sample the Brillouin zone but with grid size (4 × 4 × 1). For explicit solvation, stepwise addition of H_2_O molecules was used.^45,47^ The additional stabilizing energy provided by each additional water molecule solvating the CO_2_ reduction intermediate *A*, Ω_*A*_^*n*^ = E_*[*A*+H_2O_]_ + E_*_ – E_*A_ – E_*H_2_O_ = Δ*E*_[*A*+H_2_O]_ – Δ*E*_*A*_, is compared to the stabilizing energy provided when the water molecule is solvated by other water molecules, Ω_H_2_O_, and if Ω_*A*_^*n*^ < Ω_H_2_O_ it is assumed that the intermediate is solvated and another water molecule is added. In general, *COOH is solvated via hydrogen bonding by one water molecule (*n* = 1), whereas *CO is not solvated via hydrogen bonding (*n* = 0). We include explicit water molecules around the adsorbates and disregard the solvent beyond the first solvation shell because previous works showed that including it implicitly or explicitly does not appreciably modify Ω_*A*_^*n*^.^45,74^

### 2.3 Effect of Cations on C_1_ Intermediates

To investigate the effect of cations on CO_2_RR intermediates, (4 × 4) (111) slabs were used. These were large enough to avoid lateral interactions between adsorbates, as shown in Table S11 and Figure S7. Due to computational resource limits, 3 layers of atoms were used, where the bottom layer was fixed at the optimized bulk distances and the top two layers and any absorbates were allowed to fully relax. The most stable co-adsorption configurations for *K and C_1_ species on Cu are provided in Section S5 and Figure S8. To find those configurations we ran several calculations using the configuration in vacuum and displacing the cations around the adsorbate. The Brillouin zone was sampled using a (3 × 3 × 1) Monkhorst–Pack mesh. Ag, Au, Co, Cu, Ni, Ir, Pt, Pd and Rh surfaces were used in their spin-restricted face-centered cubic (fcc) configuration. Previous work shows that this configuration is acceptable for Co and that, although there are differences in the spin-restricted and unrestricted values for Co and Ni surfaces, the trends are unaffected.^21^ The stabilization of each intermediate by a co-adsorbed cation is modeled using eq 2.
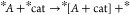
2

Again, the brackets indicate co-adsorption in proximity. And so, the stabilization energy provided by *cat, Ω_*cat_, is given by eq 3:
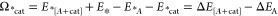
3

This definition of cation effects is analogous to that of solvation effects (see Section 2.2) and enables a direct comparison of Ω_*A*_^*n*^, Ω_H_2_O_ and Ω_*cat_.

## 3 Results and Discussion

### 3.1 Scaling Relations

**Figure 1:**
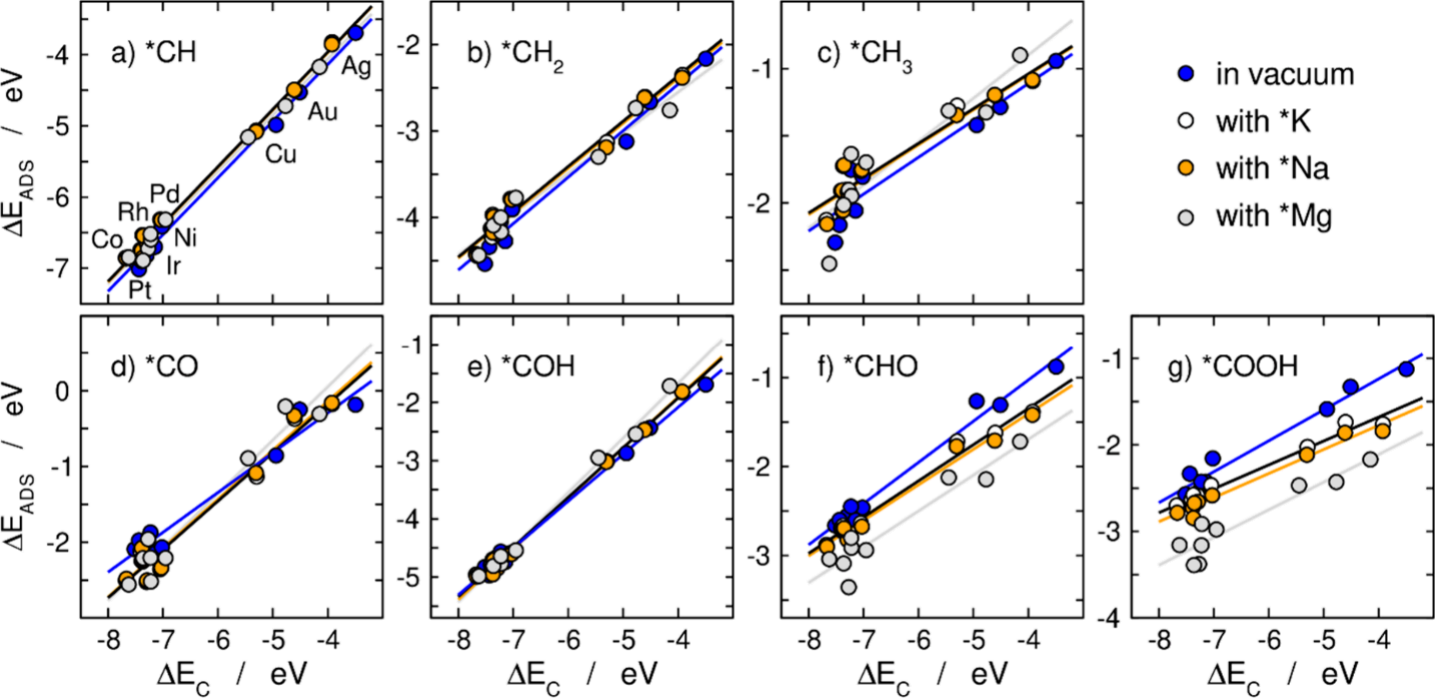
Adsorption energy scaling relations on the (111) surface of the 9 metals investigated, between *C and (a) *CH, (b) *CH_2_, (c) *CH_3_, (d) *CO, (e) *COH, (f) *CHO, and (g) *COOH.

Scaling relations for the adsorption energies of CO_2_RR intermediates are shown in Figure 1. In addition, Figure S1 presents the same data plotted with the same scale on all panels, Figure S2 contains labeled data points, and Figure S3 classifies the data depending on the presence or absence of co-adsorbed cations. The results for each intermediate in those figures are plotted vs Δ*E*_C_ and the trends are described using linear regressions the general formula of which is provided in eq 4.
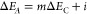
4

The slope or gradient of the line can be predicted using a simple electron counting relation, *m* = *N*_*A*_/*N*_C_, where *N*_*A*_ is the number of electrons required for an intermediate *A* to reach a full outer shell and *N*_C_ is the number of electrons for C to complete its outer shell, that is 4.^24,29^ This relation is applicable in many situations where the interaction between the surface and each species is similar. In the light of this, stepwise hydrogenation progressively lowers the gradient, for example for CH_*x*_*vs* C scaling relations, the predicted slopes are 0.75, 0.50, and 0.25 for *x* = 1, 2, 3 (see Table S1).^24^ In addition, atoms with the same number of valence electrons (*e.g*., C and Si) scale linearly with a gradient of 1.^29^ However, other factors can influence the slope such as solvation or covalent character in the interaction between the adsorbed intermediate and the surface.^34^ Furthermore, scaling relations may or may not exist depending on the chemical nature of the adsorbates and the composition and coordination number of the adsorption sites.^75^Table S1 in the Supporting Information contains the slopes, offsets, correlation coefficients, mean and maximum absolute deviations (MADs and MAXs) of all linear fits in Figure 1. The overall MAD and MAX are 0.09 and 0.39 eV. We note that *Mg co-adsorption generally lowers the correlation coefficients and increases the MADs and MAXs.

**Figure 2:**
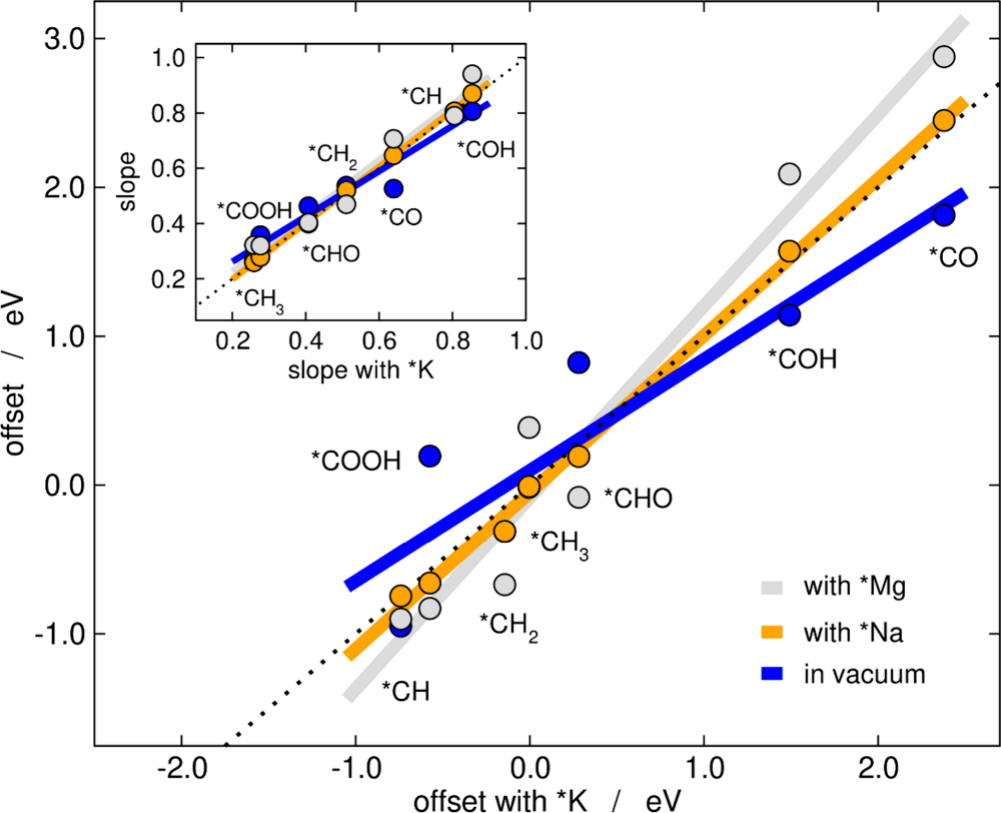
Offsets of the scaling relations with and without co-adsorbed cations shown in Figure 1 as a function of those with *K. Inset: slopes of the scaling relations in vacuum, with *Na, and with *Mg as a function of those with *K. Following the nomenclature of eq 4 and Table S1, the main panel depicts *i*_A_^vac^, *i*_A_^*Na^ and *i*_A_^*Mg^ vs *i*_A_^*K^, and the inset depicts *m*_A_^vac^, *m*_A_^*Na^ and *m*_A_^*Mg^ vs *m*_A_^*K^. The dashed lines are parity lines.

Figure 2 condenses all the slopes and offsets of the scaling relations in Figure 1. The data in vacuum, with *Na and *Mg are presented as a function of the data with *K. As discussed in the next paragraphs, Figure 2 shows that the slopes are linearly related, and so are the offsets, which is why we posit that cation effects are systematic and predictable among C_1_ species on transition metals.

For the intermediates alone on the surface, shown in blue in Figure 1, the slopes follow the literature and abide by the expected valence electron relationship. As shown in the inset of Figure 2, intermediates where *N*_*A*_ is 3, (*CH and *COH) have gradients close to 0.75, where *N*_*A*_ is 2 (*CH_2_, *CO, and *CHO) the slope is close to 0.5, and where *N*_*A*_ is 1 (*CH_3_ and *COOH) the slope is close to 0.25 see Table S1 for details. More importantly, the inset of Figure 2 and Figure S4 show that upon co-adsorption of *K, *Na, and *Mg, cation effects modify the scaling relations observed in vacuum: the slopes of *COH and *CO increase, those of *CH_2_, *CHO, *COOH decrease, and those of *CH and *CH_3_ remain approximately constant (except for *CH_3_ with *Mg). These changes in the slopes suggest that *N*_*A*_ is partially modified by the interaction of the adsorbates with the cations. For instance, for CO, as the electronegativity of the cation increases, the slope increases from close to 0.5 to close to 0.75 suggesting a loss of the double bond character in *CO and a change from 2 to 1 electron needed to complete the valence shell. Previous work has shown that for CO the stretching vibration, which is inversely proportional to the bond strength, is affected by approaching cations, which supports the change in slope and, hence, in relative bond orders, observed here.^57,76,77^

Appreciable changes are also observed in the offset upon addition of a co-adsorbed cation, as shown in Figure 2 and Figure S5. The offsets of the scaling relations are linearly related in presence and absence of cations and some offsets are made more negative than in vacuum by cation co-adsorption and some others are made less negative. Interestingly, the variations have the same direction as those of the slopes: the offsets of *COH and *CO increase, those of *CH_2_, *CHO, *COOH decrease, and those of *CH and *CH_3_ remain approximately constant (except for *CH_3_ with *Mg). In principle, Figure 2 and Figures S4 and S5 imply that cation effects can either stabilize or destabilize the adsorption of a given species on a series of metals, and the magnitude and direction of the effects can be predicted. Our main conclusion from Figure 2 is that cations can be used to modify adsorption energies without changing the morphology of the active sites. As will be shown later, this can be used in electrocatalysis to activate or deactivate specific active sites.

**Figure 3:**
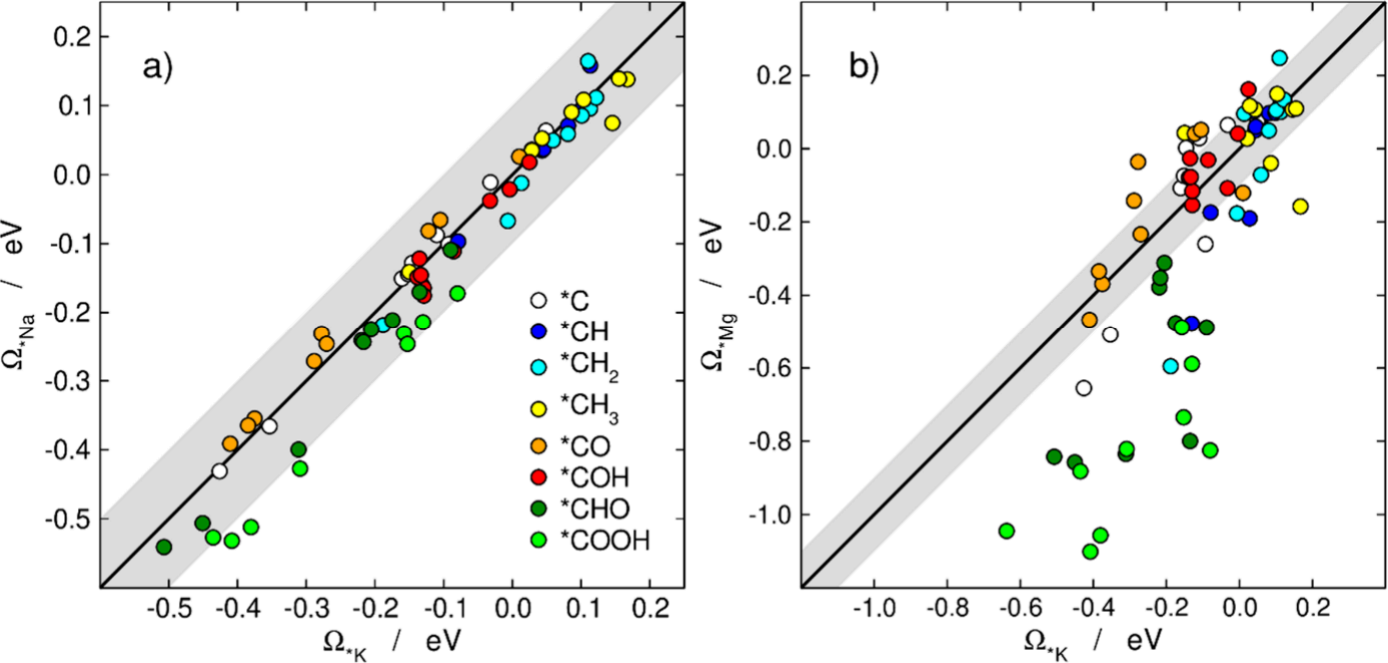
Energy stabilization provided by (a) *Na (Ω_*Na_) and (b) *Mg (Ω_*Mg_) plotted against the energy stabilization provided by *K (Ω_*K_) for C_1_ intermediates in the reduction of CO_2_ on nine transition metals. Data points over the parity line *y* = *x* indicate that both cations influence the adsorbate identically. The MAEs are 0.03 and 0.18 eV for Ω_*Na_ vs Ω_*K_ and Ω_*Mg_*vs* Ω_*K_, respectively, and the gray bands correspond to ±0.10 eV around the parity line.

Figure 3a,b are parity plots to illustrate how the stabilization varies depending on the cation. Figure 3a shows how the difference in binding energy between the sole intermediate and the intermediate in the presence of *K, Ω_*K_ (see eq 3), compares to the presence of *Na, Ω_*Na_, for each of the intermediates. If the ions affect the intermediates in the same manner, then there will be a linear relationship between Ω_*K_ and Ω_*Na_ with a slope of 1 and a null offset. To show the expected relationship, the parity line *y* = *x* is included with an error band around it of ±0.10 eV. For these two cations the mean absolute deviation (MAD) is 0.03 eV. We observe that all the intermediates, apart from *COOH, are close to the parity line, indicating that they are stabilized in a similar way by both *K and *Na for all metals. *COOH is stabilized more by *Na than *K by 0.10 eV, on average, and the effect is essentially constant for all metals.

The same analysis was carried out to determine the correlation between stabilization by *Mg and *K and the results plotted in Figure 3b. Here, the MAD was 0.18 eV, and we observe that the deviations grow as Ω_*K_ becomes increasingly negative. For each intermediate, the parameters for the linear fit for Ω_*Mg_*vs* Ω_*K_ in Figure 3b are shown in Table S2. Altogether, Figure 3a,b clearly indicates that potassium and sodium stabilize the adsorbates approximately in the same way, while magnesium affects the intermediates differently. Hence, one can easily predict the adsorbate stabilization provided by *Na based on the one provided by *K and vice versa, but this is not the case for the stabilization granted by *Mg.

**Figure 4:**
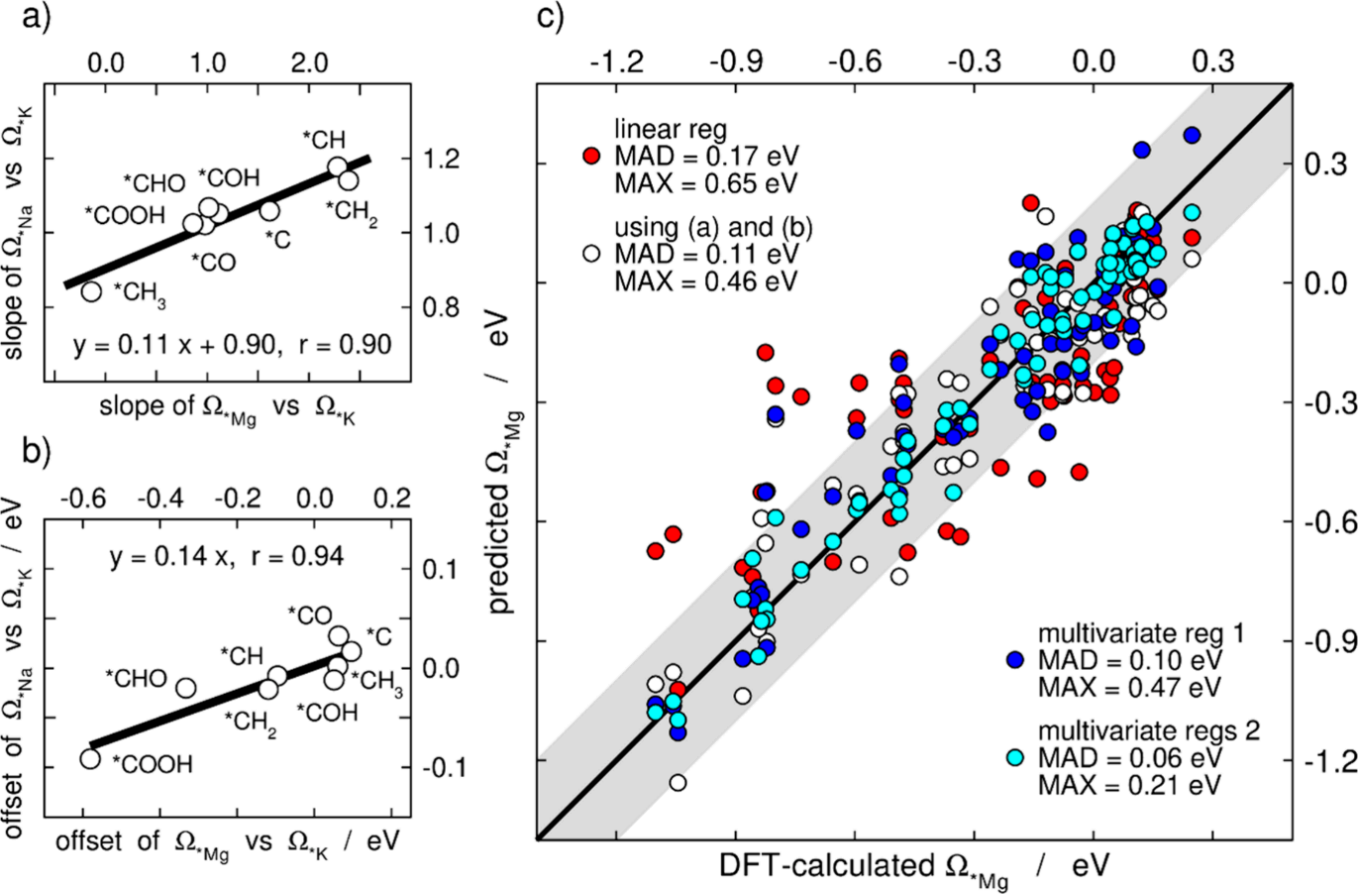
Correlations between (a) the slopes and (b) the offsets of the linear fits of Ω_*Na_*vs* Ω_*K_ and Ω_*Mg_*vs* Ω_*K_. (c) Parity plot comparing the DFT-calculated stabilization granted by *Mg (Ω_*K_) and the stabilization predicted by means of a linear fit (red), an equation based on the parameters in panels (a) and (b) (white), a multivariate regression using Ω_*K_ and Ω_*Na_ (blue), and multivariate regressions using Ω_*K_, the number of valence electrons of the transition metal and the *d*-series it belongs to (cyan). The equations of the regressions appear in Section S4. The gray band covers an area of ±0.20 eV around the parity line.

This poses an important question: Is it possible to predict the effects due to *Mg co-adsorption based on those of *K and/or *Na? Seeking an answer, we resorted to four approaches: *(i)* a linear regression of the data in Figure 3b; *(ii)* a correlation of the Ω_*Mg_*vs* Ω_*K_ parameters with those of Ω_*Na_*vs* Ω_*K_ (see Table S2), which are found in Figure 4a,b to be linearly related; *(iii)* a multivariate regression of Ω_*Mg_ as a function of Ω_*K_ and Ω_*Na_; and *(iv)* per-adsorbate multivariate regressions using 3 input variables, namely Ω_*K_, the number of valence electrons of the transition metals (9 for Co, Rh, Ir; 10 for Ni, Pd, Pt; 11 for Cu, Ag, Au) and their *d*-series (3d, 4d, 5d).

Figure 4c shows that the linear fit is the least accurate of the three approaches, and the MAD of the predictions is 0.17 eV while the maximum absolute deviation (MAX) is 0.65 eV. Only 65% of the data are within ±0.20 eV of the parity line in Figure 4c. This was expected judging by the large departures from the parity plot in Figure 3b. Furthermore, capitalizing on the linear relationships in Figure 4a,b and the parameters in Table S2, the MAD can be lowered to 0.11 eV and the MAX to 0.46 eV. Now, 83% of the data are within ±0.20 eV of the parity line in Figure 4c. Similar accuracy, namely MAD/MAX of 0.10/0.47 eV, is delivered by a simpler multivariate regression using Ω_*Na_ and Ω_*K_ as inputs. In addition, 85% of the data are within ±0.20 eV of the parity line in Figure 4c. Multivariate regressions using Ω_*K_, the number of valence electrons of the transition metals and the location in the *d*-series lead to MAD and MAX values as low as 0.06 and 0.21 eV, see Section S4. In fact, 99% of the predictions are within ±0.20 eV of the parity line, and 86% are in the narrow range of ±0.10 eV of the parity line. In the light of Figure 4c, we conclude that it is possible to predict *Mg stabilization effects on C_1_ species with reasonable accuracy by combining the respective data for *K and *Na or using the data for *K and two simple descriptors trivially obtained from the periodic table.

### 3.2 Application to the Reduction of CO_2_ to CO

**Figure 5:**
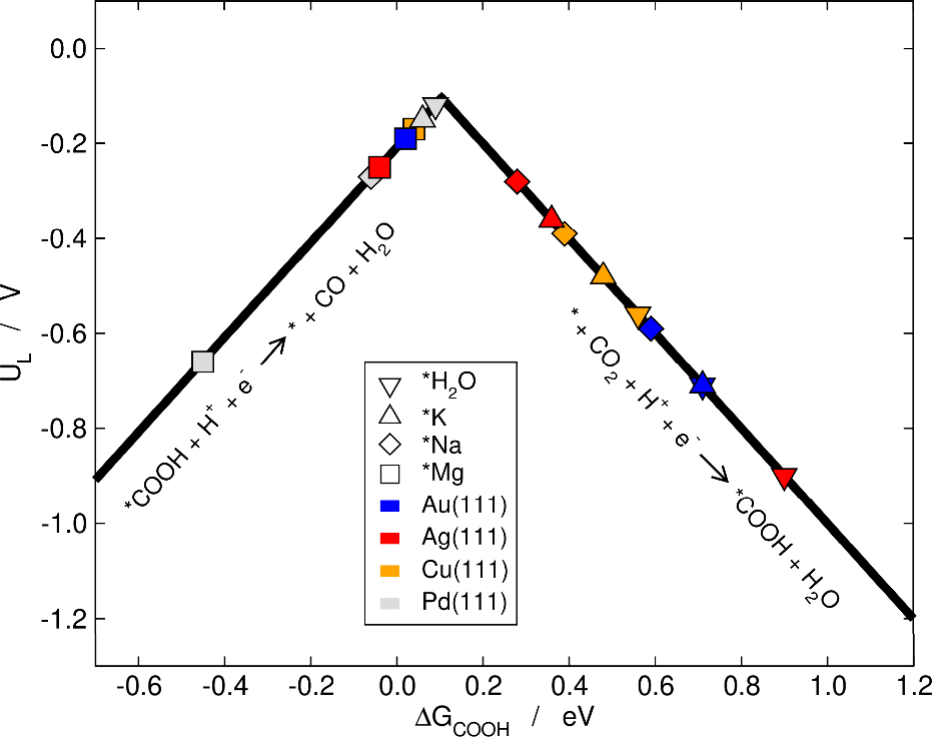
Volcano plot for the reduction of CO_2_ to CO via *COOH on Au(111) (blue), Ag(111) (red), Cu(111) (orange), and Pd(111) (gray). Results in vacuum and with three different cations are provided for each metal. The data for Au(111) and Ag(111) with *H_2_O with water were taken from ref (68).

The reduction of CO_2_ to form CO via *COOH is the first stage common to the production of numerous CO_2_ reduction products with one carbon atom with the exception of formic acid, which is typically formed via *OCHO.^49^ To illustrate the electrocatalytic effects of cation co-adsorption, in the following we compare the reduction of CO_2_ to CO on four experimentally relevant surfaces with and without cations. Previous experimental work showed an increase of the CO_2_RR activity and selectivity on Cu, Pd and Au single-crystal electrodes at subzero temperatures in highly concentrated brines.^51^ While the authors proposed that the change in catalytic activity and selectivity was mainly associated with an increase of the CO_2_ solubility at low temperatures, they also echoed on possible effects of the highly concentrated cations and the water availability at the interface.^51^ In addition to those metals, Ag has also been highlighted in Figure 5 given its academic and industrial relevance in the CO_2_RR to CO. The first proton–electron transfer is * + CO_2_ + H^+^ + e^–^ → *COOH (Δ*G*_1_ = Δ*G*_COOH_). In turn, the second proton–electron transfer is *COOH + H^+^ + e^–^ → * + CO + H_2_O (Δ*G*_2_ = Δ*G*^0^ – Δ*G*_COOH_, where Δ*G*^0^ is the overall reaction energy). The limiting potential is given as *U*_L_ = −max (Δ*G*_1_, Δ*G*_2_)/e^–^. We emphasize that, as only electrochemical steps are considered in Figure 5, the volcano stands alone for metals that bind *CO weakly or moderately, such as Au, Ag and Cu. However, it is advisable to collate the volcano with the separate free-energy diagram for strong-binding metals, such as Pd. The free-energy diagrams for the four metals are provided in Figure S6.

According to Figure 5, the first proton–electron transfer is the potential limiting step when *COOH is in contact with *H_2_O for Au(111), Ag(111), and Cu(111) surfaces, which implies that they are all on the weak-binding side of the volcano. Cation stabilization of *COOH generally enhances the activity of Au(111), Ag(111) and Cu(111) and *Mg has a more pronounced effect than *Na and *K. Interestingly, stabilization by *Mg changes the potential limiting step on these three metals to the second electrochemical step. On the other hand, Pd(111) with *H_2_O is on the strong-binding side of the volcano and close to the apex, the presence of the three cations does not change the potential limiting step and in all cases increases the overpotential. In addition, we stress that the desorption of *CO to CO_(g)_ is rather energetically unfavorable on Pd(111) with and without cations, as illustrated in Figure S6, such that Pd(111) is generally expected to be blocked by *CO.

The main piece of information we extract from Figure 5 is that cations activate weak-binding metals and facets for CO_2_ electroreduction to CO. This gives another perspective on the observation in the literature that cationic surfactants activate Ag surfaces for CO_2_ reduction, as the enhancement is usually attributed to the suppression of hydrogen evolution.^54,78−80^ Furthermore, only a few works have probed the effect of Mg^2+^ during the CO_2_RR. Besides the well-known work by Monteiro *et al*.^81^ in nearly acidic media on Au electrodes, in a previous work^51^ the onset potential for the CO_2_RR on Cu electrodes in Mg(ClO_4_)_2_ brines was observed as low as −0.25 V *vs* RHE. Wang et al.^82^ also reported an enhancement of the CO_2_RR on CoPc in the presence of Mg^2+^ ions. Along the same line, Lyu et al.^83^ reported and enhancement in the CO_2_RR on CoPc anchored to Mg(OH)_2_ substrates, which allegedly facilitate the formation of the *CO_2_^–^ intermediate.

Similarly, according to the *in situ* measurements in ATR configuration reported by Zhu *et al*.,^84^ during the CO_2_RR in carbonate solutions on polycrystalline Cu electrodes, the CO bands start appearing at potentials as low as −0.2 V *vs* RHE. Finally, we note that Seong *et al*.^85^ observed the formation of *CO on Au sites during the CO_2_RR on AuCu catalyst in phosphate buffer solution (pH 7–2) at potentials as low as −0.3 V *vs* RHE.

To close this section, we emphasize that our calculations were carried out considering only low surface coverage of species on the (111) facet of transition metals. Future, more comprehensive works ought to systematically examine coverage effects of adsorbates, cations, and spectators on scaling relations and electrocatalytic activity predictions with structural sensitivity, as done elsewhere.^55,86,87^

## 4 Conclusions

This work provides a trends-based view of cation effects on C-based species. The presence of *K, *Na and *Mg changes the adsorption energies of C_1_ species, and the magnitude and sign of the effect depend on the specific transition metals which they adsorb on. Because we found that the effects are systematic and gradually change the slopes and the offsets of adsorption-energy scaling relations, we conclude that cations change surface–adsorbate interactions both qualitatively and quantitatively.

Since the stabilization granted by *K and *Na is similar, a simple linear regression suffices to predict one provided that the other is known. However, the stabilization granted by *Mg is different from those of *K and *Na and the differences accentuate alongside the magnitude of the stabilization effect. In view of that, multivariate regressions were provided that enable the accurate prediction of stabilization due to *Mg in terms of *(i)* those of *K and *Na, and *(ii)* that of *K and two simple descriptors obtained from the periodic table.

Analyzing CO_2_ electroreduction to CO by means of a Sabatier-type volcano plot and free-energy diagrams led us to conclude that cation effects activate weak-binding metals, such as Au, Ag, and Cu, and weak-binding facets such as the (111). The enhancement is because *COOH formation is usually energy intensive, but the presence of cations sizably lowers its potential requirements.

In summary, we have shown here that cation effects on transition metals follow systematic trends. This opens new perspectives in electrocatalysis, as cation effects are most often analyzed separately, that is considering only a specific cation, on a given facet of a specific metal. The fact that the effects are systematic gives hope for a straightforward incorporation of cation effects in materials screening routines, circumventing the need for demanding calculations with numerous atoms or advanced yet arduous treatments of the double layer.

## Supporting Information Available

Parameters for graphs in the main text, tabulated adsorption energies, supplementary figures and equations, converged geometries in CONTCAR format (PDF)
