# Supplementary material for: Cation Effects on the Adsorbed Intermediates of CO2 Electroreduction Are Systematic and Predictable
Source: ACS Catal. 2024 May 23;14(11):8814–22. doi: 10.1021/acscatal.4c00727 (PMC11165452; doi:10.1021/acscatal.4c00727)
Supplement: Supplementary file 1 — cs4c00727_si_001.pdf [file cs4c00727_si_001.pdf]

Supporting Information

Cation effects on the adsorbed intermediates of CO_2 electroreduction are systematic and predictable

Elizabeth Sargeant,[a,b] Paramaconi Rodriguez, [a,c,d,*] Federico Calle-Vallejo[b,d,e,*]

[a] School of Chemistry, University of Birmingham, Edgbaston, Birmingham, B15 2TT, United Kingdom.
[b] Department of Materials Science and Chemical Physics & Institute of Theoretical and Computational Chemistry (IQTC), University of Barcelona, Barcelona, 08028, Spain.
[c] Centre for Cooperative Research on Alternative Energies (CICenergiGUNE), Basque Research and Technology Alliance (BRTA), Alava Technology Park, 01510 Vitoria-Gasteiz, Spain.
[d] IKERBASQUE, Basque Foundation for Science, Plaza de Euskadi 5, 48009 Bilbao, Spain.
[e] Nano-Bio Spectroscopy Group and European Theoretical Spectroscopy Facility (ETSF), Department of Advanced Materials and Polymers: Physics, Chemistry and Technology, University of the Basque Country UPV/EHU, Avenida Tolosa 72, 20018 San Sebastian, Spain.

Emails: prodriguez@cicenergigune.com (PR); federico.calle@ehu.es (FCV)

Table of contents

S1. Parameters for graphs in the main text S2
S2. Adsorption energies S3
S3. Supplementary figures S6
S4. Supplementary equations S11
S5. Co-adsorption configurations and CONTCARs S12

S1. Parameters for graphs in the main text

Table S1. *Parameters from the linear fits in Figure 1. m, i and R are the slope, offset and correlation coefficient of the scaling relations.*

Parameter	*Cation*	*Adsorbed intermediate*						
		CH	CH_2	CH_3	CO	COOH	COH	CHO
m	-	0.80	0.54	0.27	0.53	0.36	0.81	0.46
	*K	0.81	0.52	0.26	0.64	0.28	0.85	0.41
	*Na	0.81	0.52	0.26	0.65	0.28	0.87	0.40
	*Mg	0.79	0.47	0.32	0.71	0.32	0.94	0.40
i / eV	-	-0.95	-0.31	-0.01	1.81	0.20	1.14	0.82
	*K	-0.74	-0.27	0.00	2.38	-0.57	1.49	0.28
	*Na	-0.75	-0.31	-0.01	2.45	-0.66	1.57	0.19
	*Mg	-0.90	-0.67	0.39	2.88	-0.83	2.09	-0.08
R	-	0.99(7)	0.99	0.95	0.98	0.98	0.99(7)	0.99
	*K	0.99(6)	0.99	0.96	0.98	0.99	0.99(8)	0.99
	*Na	0.99(7)	0.99	0.96	0.98	0.99	0.99(8)	0.99
	*Mg	0.99(6)	0.98	0.91	0.97	0.94	0.99(7)	0.95
MAD / eV	-	0.08	0.11	0.11	0.14	0.09	0.08	0.06
	*K	0.09	0.10	0.08	0.14	0.05	0.07	0.07
	*Na	0.08	0.09	0.08	0.14	0.05	0.06	0.06
	*Mg	0.07	0.11	0.13	0.17	0.12	0.08	0.13
MAX / eV	-	0.14	0.19	0.24	0.31	0.16	0.12	0.20
	*K	0.21	0.18	0.19	0.24	0.11	0.13	0.16
	*Na	0.17	0.17	0.20	0.25	0.13	0.12	0.16
	*Mg	0.18	0.18	0.39	0.30	0.23	0.15	0.34

Table S2. *Parameters for the linear fits of the data in Figure 3 for Ω_{*Mg} vs. Ω_{*K} and Ω_{*Na} vs. Ω_{*K} for each intermediate.*

	C	CH	CH_2	CH_3	CO	COOH	COH	CHO
$m_{Mg\ vs\ K}$	1.61	2.28	2.39	-0.14	0.98	0.86	1.11	1.02
$i_{Mg\ vs\ K}$	0.10	-0.10	-0.12	0.06	0.06	-0.58	0.05	-0.33
$R_{Mg\ vs\ K}$	0.91	0.90	0.94	0.14	0.76	0.75	0.74	0.61
$m_{Na\ vs\ K}$	1.06	1.18	1.14	0.84	1.02	1.02	1.05	1.07
$i_{Na\ vs\ K}$	0.02	-0.01	-0.02	0.00	0.03	-0.09	-0.01	-0.02
$R_{Na\ vs\ K}$	0.99(8)	0.99	0.95	0.97	0.99(7)	0.99	0.97	0.99

S2. Adsorption energies

Table S3. *Adsorption energies (in eV) of *C, [*C + K], [*C + Na] and [*C + Mg] on (111) terraces.*

metal	ΔE_{C}	$\Delta E_{[C+K]}$	$\Delta E_{[C+Na]}$	$\Delta E_{[C+Mg]}$
Ag	-3.49	-3.92	-3.93	-4.15
Au	-4.51	-4.60	-4.61	-4.77
Co	-7.52	-7.68	-7.67	-7.62
Cu	-4.94	-5.29	-5.31	-5.45
Ir	-7.30	-7.41	-7.39	-7.27
Ni	-7.15	-7.30	-7.29	-7.22
Pd	-7.02	-7.05	-7.03	-6.95
Pt	-7.44	-7.39	-7.37	-7.36
Rh	-7.23	-7.37	-7.36	-7.23

Table S4. *Adsorption energies (in eV) of *CH, [*CH + K], [*CH + Na] and [*CH + Mg] on (111) terraces.*

metal	ΔE_{CH}	$\Delta E_{[CH+K]}$	$\Delta E_{[CH+Na]}$	$\Delta E_{[CH+Mg]}$
Ag	-3.69	-3.82	-3.85	-4.17
Au	-4.53	-4.50	-4.49	-4.72
Co	-6.89	-6.85	-6.86	-6.85
Cu	-4.99	-5.06	-5.08	-5.16
Ir	-6.82	-6.74	-6.75	-6.72
Ni	-6.70	-6.66	-6.66	-6.60
Pd	-6.41	-6.31	-6.32	-6.32
Pt	-7.01	-6.90	-6.86	-6.89
Rh	-6.58	-6.54	-6.54	-6.52

Table S5. *Adsorption energies (in eV) of *CH_2, [*CH_2 + K], [*CH_2 + Na] and [*CH_2 + Mg] on (111) terraces.*

metal	ΔE_{CH2}	$\Delta E_{[CH2+K]}$	$\Delta E_{[CH2+Na]}$	$\Delta E_{[CH2+Mg]}$
Ag	-2.16	-2.35	-2.38	-2.76
Au	-2.66	-2.61	-2.61	-2.73
Co	-4.54	-4.42	-4.44	-4.44
Cu	-3.12	-3.13	-3.19	-3.30
Ir	-4.14	-4.13	-4.15	-4.04
Ni	-4.27	-4.17	-4.19	-4.17
Pd	-3.91	-3.78	-3.79	-3.77
Pt	-4.34	-4.23	-4.17	-4.09
Rh	-4.05	-3.97	-3.99	-4.00

Table S6. *Adsorption energies (in eV) of *CH$_3$, [*CH$_3$ + K], [*CH$_3$ + Na] and [*CH$_3$ + Mg] on (111) terraces.*

metal	ΔE_{CH3}	$\Delta E_{[CH3+K]}$	$\Delta E_{[CH3+Na]}$	$\Delta E_{[CH3+Mg]}$
Ag	-0.94	-1.09	-1.08	-0.90
Au	-1.29	-1.20	-1.20	-1.33
Co	-2.29	-2.13	-2.16	-2.45
Cu	-1.42	-1.28	-1.35	-1.31
Ir	-1.93	-1.91	-1.91	-1.90
Ni	-2.06	-1.90	-1.92	-1.95
Pd	-1.80	-1.76	-1.75	-1.70
Pt	-2.16	-2.06	-2.06	-2.01
Rh	-1.75	-1.72	-1.72	-1.64

Table S7. *Adsorption energies (in eV) of *CO, [*CO + K], [*CO + Na] and [*CO + Mg] on (111) terraces.*

metal	ΔE_{CO}	$\Delta E_{[CO+K]}$	$\Delta E_{[CO+Na]}$	$\Delta E_{[CO+Mg]}$
Ag	-0.18	-0.17	-0.16	-0.31
Au	-0.25	-0.37	-0.33	-0.21
Co	-2.09	-2.50	-2.48	-2.56
Cu	-0.86	-1.13	-1.09	-0.89
Ir	-2.01	-2.12	-2.08	-1.96
Ni	-2.15	-2.53	-2.51	-2.52
Pd	-2.07	-2.36	-2.34	-2.21
Pt	-1.98	-2.25	-2.22	-2.21
Rh	-1.87	-2.26	-2.24	-2.21

Table S8. *Adsorption energies (in eV) of *COOH, [*COOH + K], [*COOH + Na] and [*COOH + Mg] on (111) terraces.*

metal	ΔE_{COOH}	$\Delta E_{[COOH+K]}$	$\Delta E_{[COOH+Na]}$	$\Delta E_{[COOH+Mg]}$
Ag	-1.12	-1.76	-1.84	-2.17
Au	-1.33	-1.74	-1.86	-2.43
Co	-2.57	-2.70	-2.78	-3.16
Cu	-1.59	-2.02	-2.11	-2.47
Ir	-2.55	-2.63	-2.73	-3.38
Ni	-2.43	-2.58	-2.66	-2.92
Pd	-2.16	-2.47	-2.58	-2.98
Pt	-2.33	-2.71	-2.85	-3.39
Rh	-2.43	-2.58	-2.67	-3.16

Table S9. *Adsorption energies (in eV) of *COH, [*COH + K], [*COH + Na] and [*COH + Mg] on (111) terraces.*

metal	ΔE_{COH}	$\Delta E_{[COH+K]}$	$\Delta E_{[COH+Na]}$	$\Delta E_{[COH+Mg]}$
Ag	-1.69	-1.83	-1.81	-1.72
Au	-2.43	-2.47	-2.47	-2.54
Co	-4.83	-4.96	-4.99	-4.98
Cu	-2.87	-3.01	-3.02	-2.95
Ir	-4.66	-4.79	-4.83	-4.77
Ni	-4.74	-4.83	-4.85	-4.77
Pd	-4.58	-4.59	-4.60	-4.54
Pt	-4.97	-4.95	-4.95	-4.81
Rh	-4.56	-4.70	-4.71	-4.64

Table S10. *Adsorption energies (in eV) of *CHO, [*CHO + K], [*CHO + Na] and [*CHO + Mg] on (111) terraces.*

metal	ΔE_{CHO}	$\Delta E_{[CHO+K]}$	$\Delta E_{[CHO+Na]}$	$\Delta E_{[CHO+Mg]}$
Ag	-0.88	-1.38	-1.42	-1.72
Au	-1.31	-1.62	-1.71	-2.14
Co	-2.66	-2.88	-2.90	-3.04
Cu	-1.27	-1.72	-1.77	-2.12
Ir	-2.56	-2.69	-2.73	-3.36
Ni	-2.60	-2.81	-2.83	-2.91
Pd	-2.46	-2.64	-2.67	-2.94
Pt	-2.60	-2.69	-2.71	-3.09
Rh	-2.45	-2.67	-2.69	-2.80

Table S11. *Convergence test for the effect of *K (Ω_{*K}, in eV) on the adsorption energy of *CO on Cu(111).*

slab size	k-points	Ω_{*K}
2×2	6×6×1	-0.68
3×3	4×4×1	-0.28
4×4	3×3×1	-0.28

S3. Supplementary figures

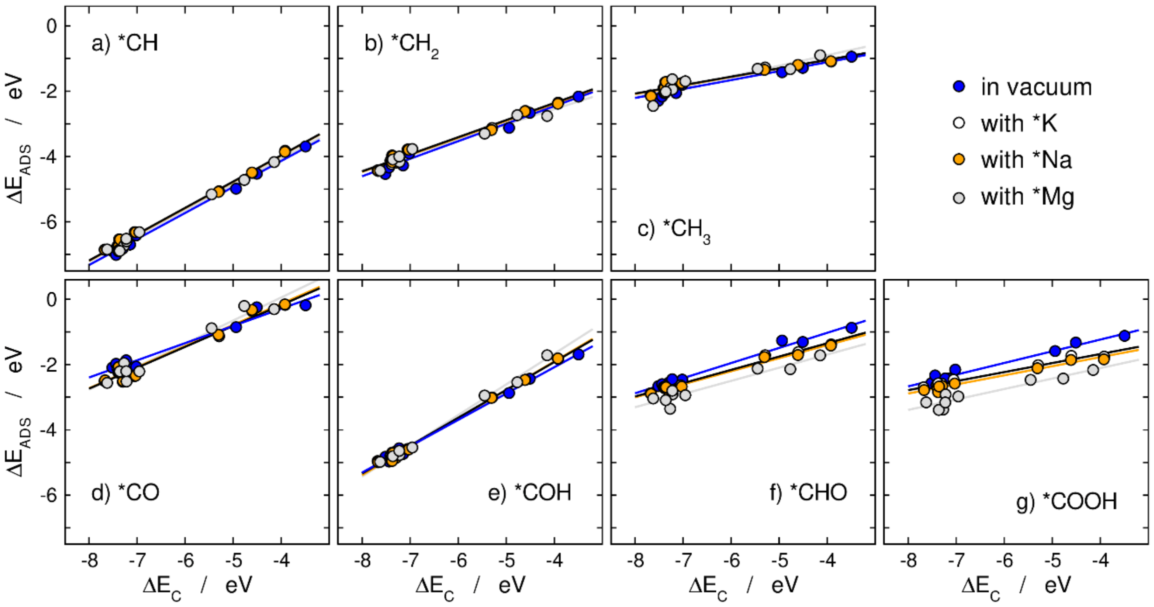

Figure S1. *Scaling relations for the binding energies of* C_1 *adsorbates vs.* ΔE_C *as in Figure 1 but with the same scale in all panels. The adsorbates are indicated in each case.*

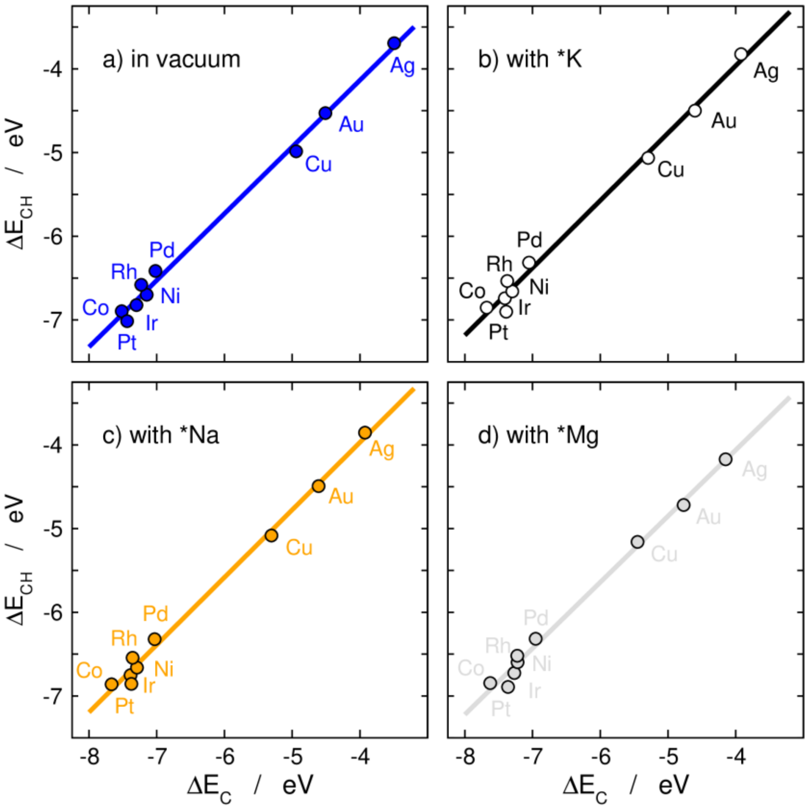

Figure S2. *Scaling relations for* ΔE_{CH} *vs.* ΔE_C *as in Figure 1a and S1a but with labelled datapoints.*

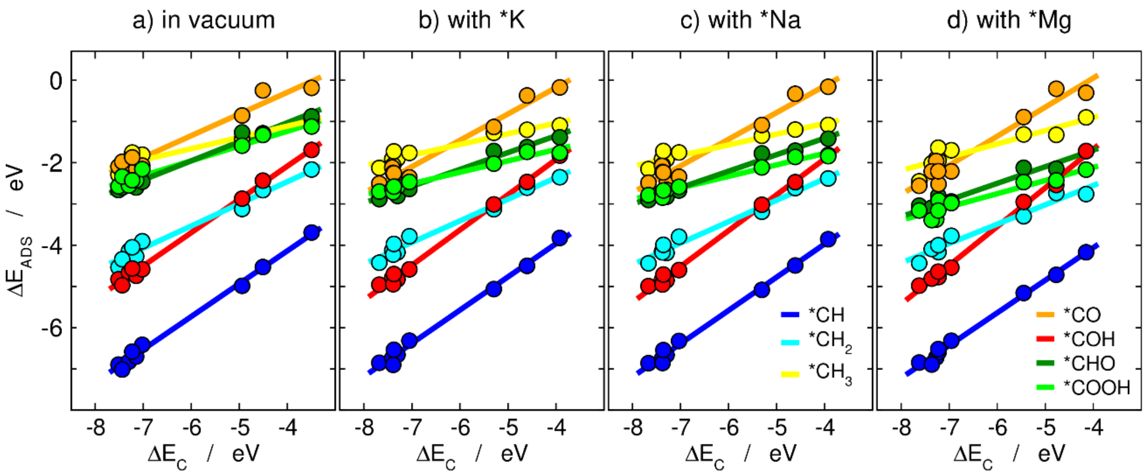

Figure S3. *Scaling relations for the binding energies of* C_1 *adsorbates vs.* ΔE_C *as in Figure 1 but separated depending on the presence or absence of specific co-adsorbed cations.*

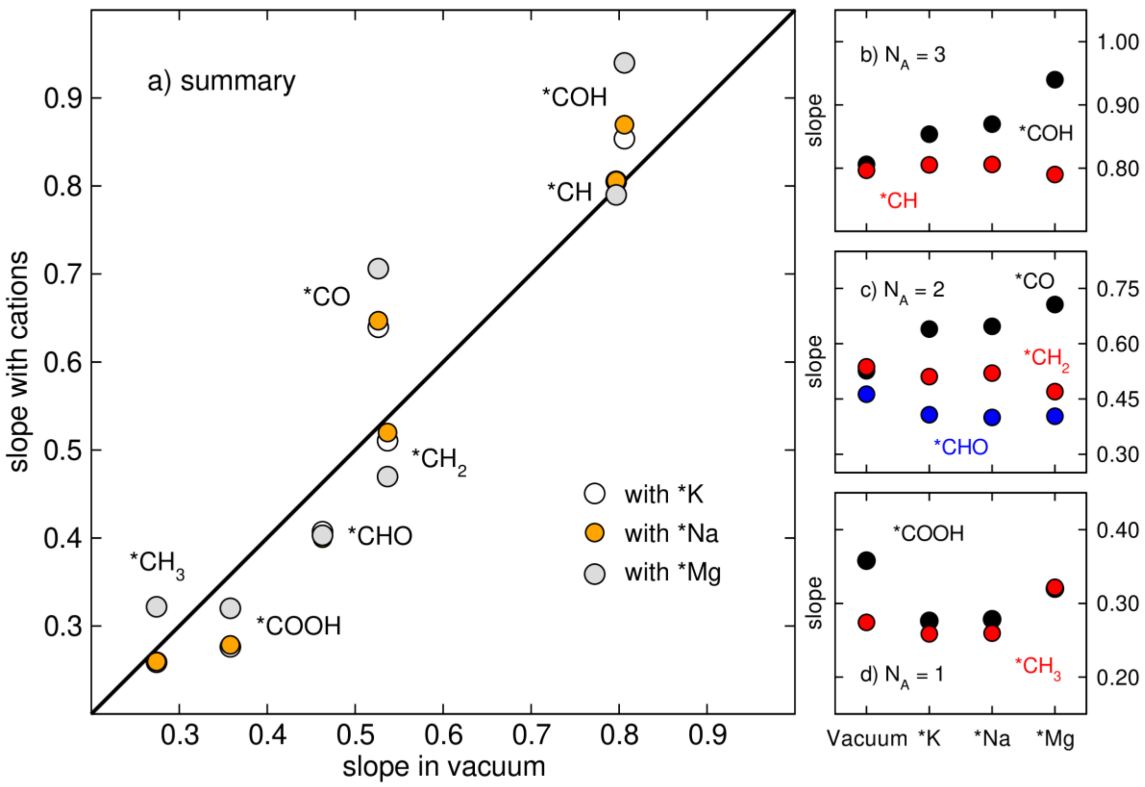

Figure S4. *a) Slopes of the scaling relations with *K, with *Na, and with *Mg as a function of those in vacuum. The slopes are also separated as a function of the valency of the adsorbates for b)* $N_A = 3$*, c)* $N_A = 2$*, d)* $N_A = 1$*.*

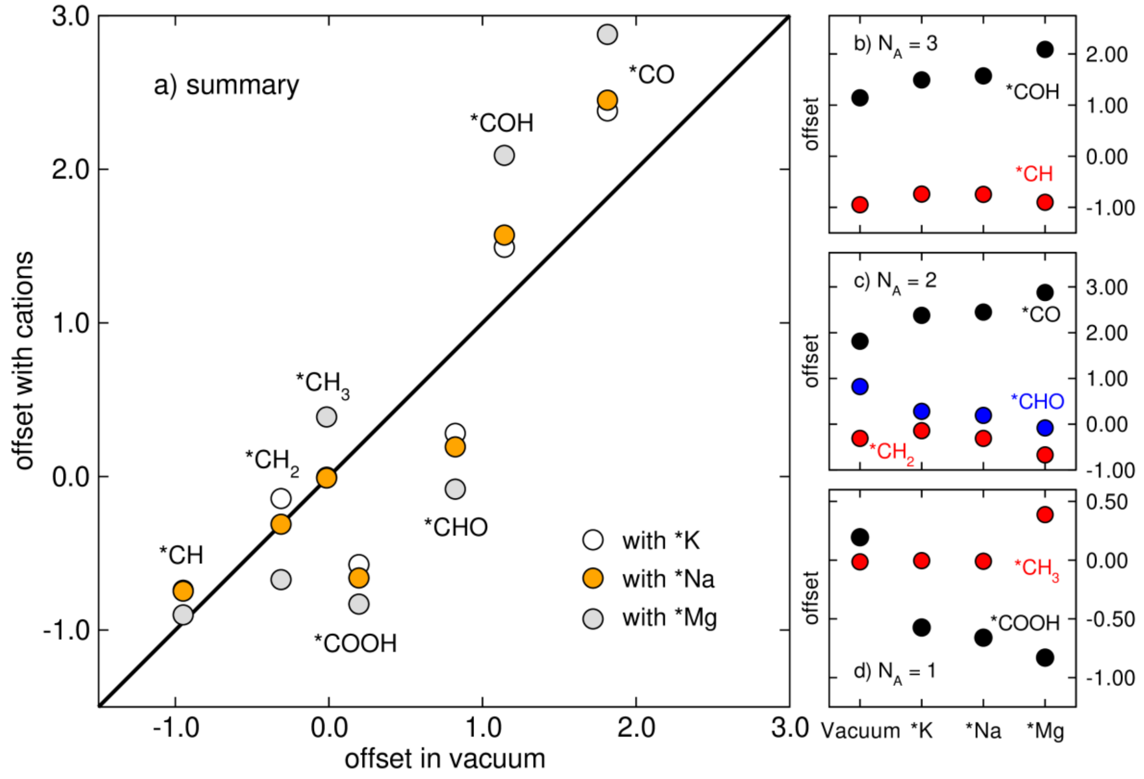

Figure S5. *a) Offset of the scaling relations with *K, with *Na, and with *Mg as a function of those in vacuum. The offsets are also separated as a function of the valency of the adsorbates for b)* $N_A = 3$*, c)* $N_A = 2$*, d)* $N_A = 1$*.*

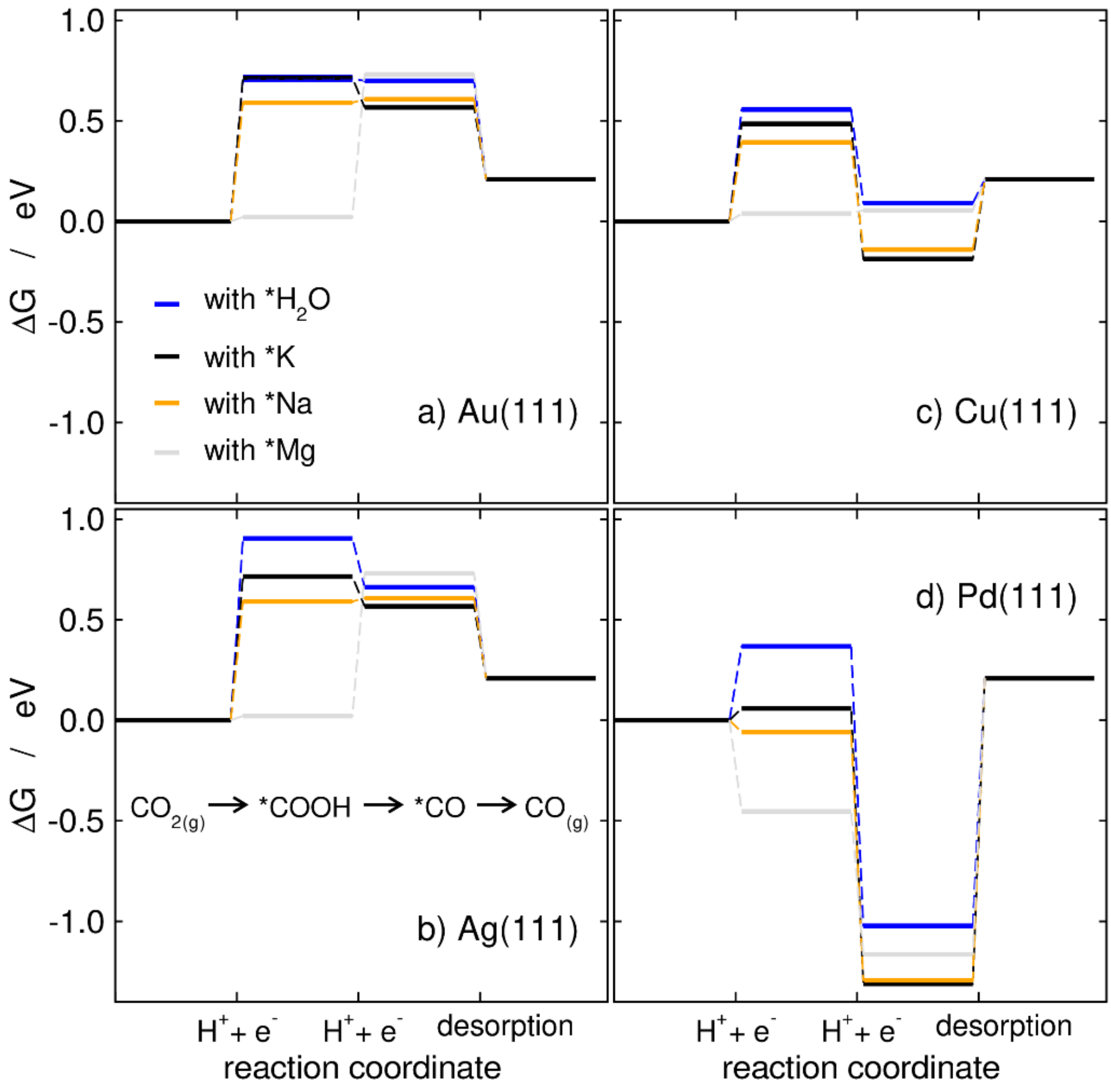

Figure S6. *Free-energy diagrams for CO_2 reduction to CO on the (111) facet of four transition metals solvated with explicit water molecules and co-adsorbed with cations. The data for Au(111) and Ag(111) with *H_2O with water was taken from Granda-Marulanda et al., ACS Catal. 10, 2020, 6900-6907.*

a) 2×2 cell, $d_{K\text{-}O}$ = 2.97 & 2.97 Å, $d_{K\text{-}K}$ = 5.14 Å

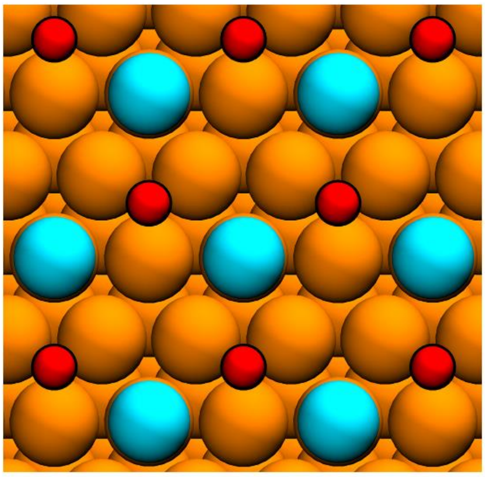

$\Omega_K = -0.68\ eV$

b) 3×3 cell, $d_{K\text{-}O}$ = 2.73 & 5.53 Å, $d_{K\text{-}K}$ = 7.72 Å

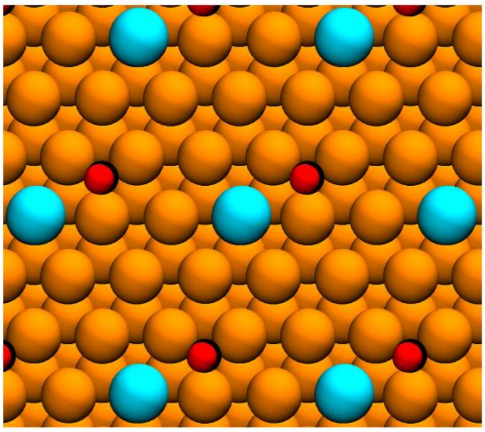

$\Omega_K = -0.28\ eV$

c) 4×4 cell, $d_{K\text{-}O}$ = 2.75 & 8.04 Å, $d_{K\text{-}K}$ = 10.29 Å

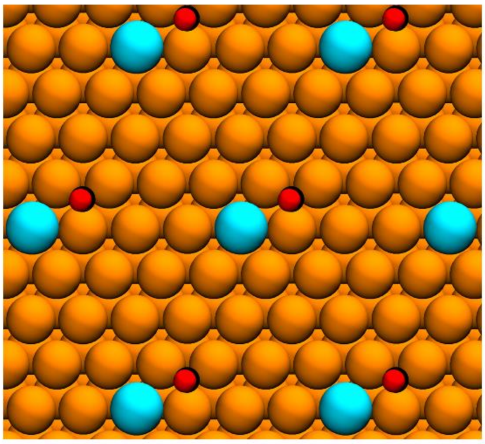

$\Omega_K = -0.28\ eV$

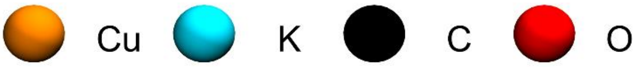

Figure S7. *Illustration of the test in Table S11. K-K and K-O distances and Ω_K are given in each case.*

S4. Supplementary equations

The equations of the linear fits in the main panel of Figure 2 are the following:

$$offset^{vacuum} = 0.75\, offset^{*K} + 0.10 \tag{S1}$$

$$offset^{*Na} = 1.05\, offset^{*K} - 0.05 \tag{S2}$$

$$offset^{*Mg} = 1.29\, offset^{*K} - 0.08 \tag{S3}$$

The equation of multivariate regression 1 in Figure 4 in the main text is:

$$\Omega_{*Mg} = 4.58\, \Omega_{*Na} - 3.40\, \Omega_{*K} - 0.01 \tag{S4}$$

The equations of the multivariate regressions 2 in Figure 4 in the main text are listed in the following:

$$\Omega_{*Mg}^{C} = 1.37 - 0.12e^{-} - 0.03d + 1.39\Omega_{*K}^{C} \tag{S5}$$

$$\Omega_{*Mg}^{CH} = 0.59 - 0.04e^{-} - 0.08d + 2.35\Omega_{*K}^{CH} \tag{S6}$$

$$\Omega_{*Mg}^{CH_2} = 0.29 - 0.06e^{-} + 0.05d + 2.14\Omega_{*K}^{CH_2} \tag{S7}$$

$$\Omega_{*Mg}^{CH_3} = -0.17 + 0.02e^{-} + 0.01d - 0.07\Omega_{*K}^{CH_3} \tag{S8}$$

$$\Omega_{*Mg}^{CO} = -0.88 + 0.06e^{-} + 0.07d + 0.58\Omega_{*K}^{CO} \tag{S9}$$

$$\Omega_{*Mg}^{COOH} = 0.07 - 0.01e^{-} - 0.15d + 0.75\Omega_{*K}^{COOH} \tag{S10}$$

$$\Omega_{*Mg}^{COH} = 0.03 + 0.01e^{-} - 0.01d + 1.15\Omega_{*K}^{COH} \tag{S11}$$

$$\Omega_{*Mg}^{CHO} = 0.57 - 0.01e^{-} - 0.17d + 1.32\Omega_{*K}^{CHO} \tag{S12}$$

S5. Co-adsorption configurations and CONTCARs

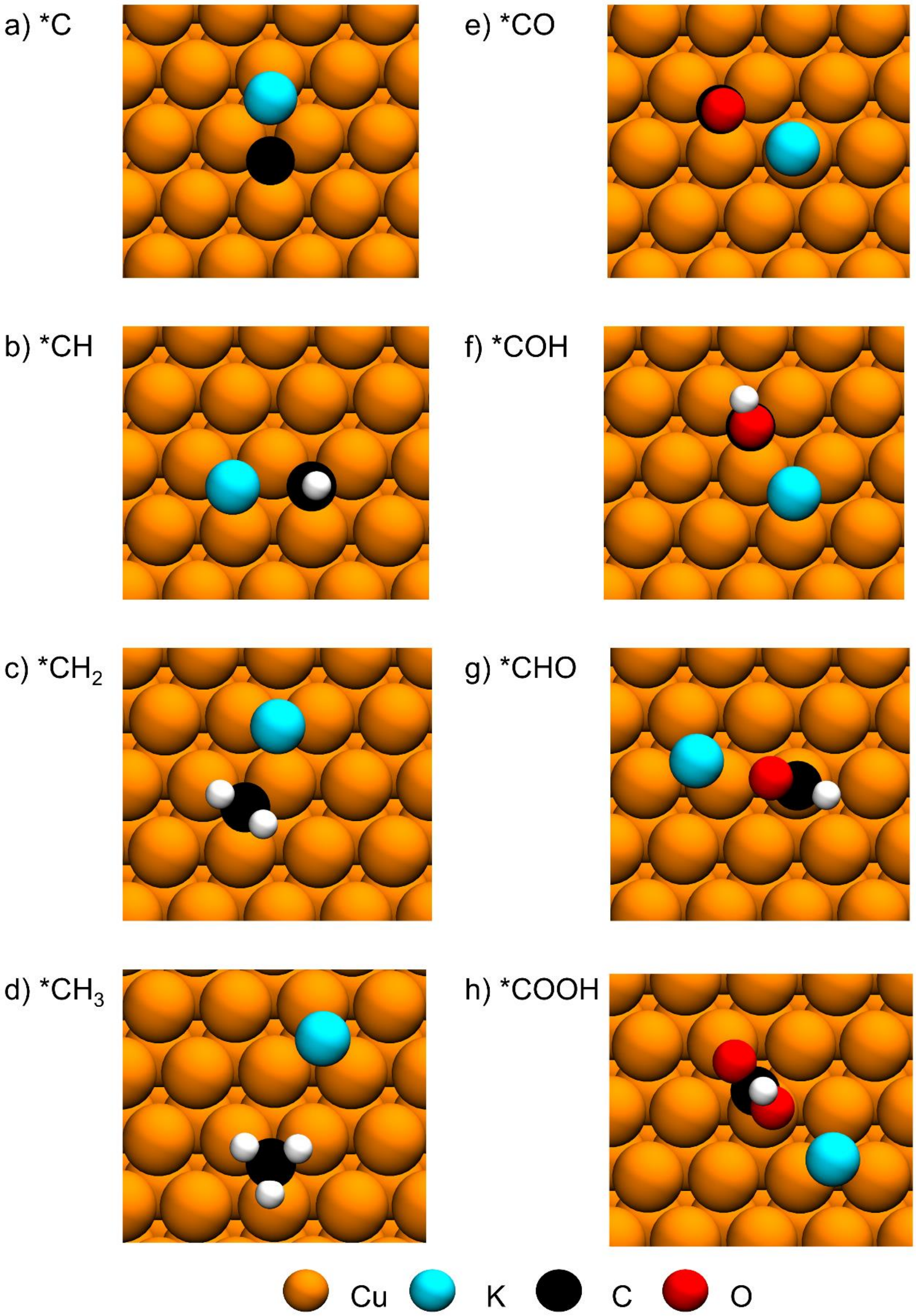

Figure S8. *Most stable configurations found for C_1 species co-adsorbed with *K on Cu(111).*

```
Cu111_C
  1.00000000000000
   10.2889409999999994    0.0000000000000000    0.0000000000000000
    0.0000000000000000    8.9104840000000003    0.0000000000000000
    0.0000000000000000    0.0000000000000000   22.0000000000000000
  Cu   C
  48     1
Selective dynamics
Direct
 -0.0000000000000000  0.9989064682534664  0.9972385544335627   T   T   T
  0.1224510042552251  0.2493487539553432  0.9972750187764792   T   T   T
  0.8775489957447747  0.2493487539553432  0.9972750187764792   T   T   T
  0.6262873020540262  0.7446177523206400  0.9961625896450035   T   T   T
  0.2482434808540875  0.9973809603595448  0.9979798898641363   T   T   T
  0.3671782124188854  0.2534180113406956  0.0010833183002639   T   T   T
  0.5000000485958651  0.9872969131253991  0.0014297915095287   T   T   T
  0.7517565191459122  0.9973809603595448  0.9979798898641363   T   T   T
  0.6328217875811146  0.2534180113406956  0.0010833183002639   T   T   T
  0.5000000485958651  0.4981963261494713  0.9973979556655386   T   T   T
  0.2470377212653259  0.5002325629513779  0.9967965356448593   T   T   T
  0.3737126979459737  0.7446177523206400  0.9961625896450035   T   T   T
  0.7529622787346740  0.5002325629513779  0.9967965356448593   T   T   T
 -0.0000000000000000  0.4994859819271359  0.9978582177353240   T   T   T
  0.8756843308126468  0.7490830706630386  0.9974335784498778   T   T   T
  0.1243156691873533  0.7490830706630386  0.9974335784498778   T   T   T
  0.1254181538936908  0.0833450018044353  0.9032767176909337   T   T   T
  0.3757076862682920  0.0819463176937913  0.9043811866257871   T   T   T
  0.2503559737629824  0.3316965426952704  0.9035182806702616   T   T   T
  0.5000000485958651  0.3307854638616798  0.9036162240604048   T   T   T
  0.6242923137317079  0.0819463176937913  0.9043811866257871   T   T   T
  0.8745818461063090  0.0833450018044353  0.9032767176909337   T   T   T
  0.7496440262370176  0.3316965426952704  0.9035182806702616   T   T   T
 -0.0000000000000000  0.3335142706131029  0.9029139170702091   T   T   T
  0.3750636837453434  0.5804780562249534  0.9031347455736235   T   T   T
  0.6249363162546565  0.5804780562249534  0.9031347455736235   T   T   T
  0.5000000485958651  0.8324585716558852  0.9035799615193090   T   T   T
  0.7505214088536981  0.8332262113978772  0.9031151016596161   T   T   T
  0.8756650669153836  0.5832071261027968  0.9028933776759819   T   T   T
  0.1243349330846164  0.5832071261027968  0.9028933776759819   T   T   T
 -0.0000000000000000  0.8335864838678204  0.9030986181747322   T   T   T
  0.2494785911463017  0.8332262113978772  0.9031151016596161   T   T   T
  0.0000000000000000  0.1666667040757801  0.8090707727272743   F   F   F
  0.2499999757020674  0.1666667040757801  0.8090707727272743   F   F   F
  0.1250000364468988  0.4166667040757801  0.8090707727272743   F   F   F
  0.3750000121489663  0.4166667040757801  0.8090707727272743   F   F   F
  0.5000000485958651  0.1666667040757801  0.8090707727272743   F   F   F
  0.7500000242979326  0.1666667040757801  0.8090707727272743   F   F   F
  0.6249999878510337  0.4166667040757801  0.8090707727272743   F   F   F
  0.8749999635531012  0.4166667040757801  0.8090707727272743   F   F   F
  0.2499999757020674  0.6666667040757801  0.8090707727272743   F   F   F
  0.5000000485958651  0.6666667040757801  0.8090707727272743   F   F   F
  0.3750000121489663  0.9166667040757801  0.8090707727272743   F   F   F
  0.6249999878510337  0.9166667040757801  0.8090707727272743   F   F   F
  0.7500000242979326  0.6666667040757801  0.8090707727272743   F   F   F
  0.0000000000000000  0.6666667040757801  0.8090707727272743   F   F   F
  0.8749999635531012  0.9166667040757801  0.8090707727272743   F   F   F
  0.1250000364468988  0.9166667040757801  0.8090707727272743   F   F   F
  0.4999999514041349  0.1648928007661585  0.0453670688904309   T   T   T
```

```
Cu111_CH
  1.00000000000000
   10.2889409999999994    0.0000000000000000    0.0000000000000000
    0.0000000000000000    8.9104840000000003    0.0000000000000000
    0.0000000000000000    0.0000000000000000   22.0000000000000000
  Cu   C   H
  48     1     1
Selective dynamics
Direct
  0.0000000000000000 -0.0001470976340238  0.0202258927380632   T   T   T
  0.1231397861540389  0.2505561321875488  0.0201480749499151   T   T   T
  0.8768602138459610  0.2505561321875488  0.0201480749499151   T   T   T
  0.6257924393897472  0.7457576736745937  0.0189574680686249   T   T   T
  0.2490100390412900  0.9982601147950619  0.0203037667904422   T   T   T
  0.3703356702355732  0.2517652713823969  0.0247121369312334   T   T   T
  0.5000000485958651  0.9892743071440204  0.0234802962359026   T   T   T
  0.7509899609587100  0.9982601147950619  0.0203037667904422   T   T   T
  0.6296643297644269  0.2517652713823969  0.0247121369312334   T   T   T
  0.5000000485958651  0.4981565793655306  0.0197892746278973   T   T   T
  0.2481676203543819  0.4999637649097504  0.0199304502759213   T   T   T
  0.3742075606102528  0.7457576736745937  0.0189574680686249   T   T   T
  0.7518323796456179  0.4999637649097504  0.0199304502759213   T   T   T
  0.0000000000000000  0.5007557508898293  0.0205451473301808   T   T   T
  0.8756524488459576  0.7499422893779086  0.0202846633220127   T   T   T
  0.1243475511540426  0.7499422893779086  0.0202846633220127   T   T   T
  0.1253566848099456  0.0836419917814818  0.9262043567701097   T   T   T
  0.3753099297079521  0.0822126615939828  0.9260805128293531   T   T   T
  0.2513044147565240  0.3314413889578639  0.9269087251509388   T   T   T
  0.5000000485958651  0.3313272220215989  0.9260527257641078   T   T   T
  0.6246900702920479  0.0822126615939828  0.9260805128293531   T   T   T
  0.8746433151900543  0.0836419917814818  0.9262043567701097   T   T   T
  0.7486955852434759  0.3314413889578639  0.9269087251509388   T   T   T
  0.0000000000000000  0.3346861426816696  0.9257735201522863   T   T   T
  0.3749562728939034  0.5806353298690858  0.9260044354014816   T   T   T
  0.6250437271060963  0.5806353298690858  0.9260044354014816   T   T   T
  0.5000000485958651  0.8337434746504879  0.9266112936584144   T   T   T
  0.7499749344927856  0.8331981552934801  0.9260789611409616   T   T   T
  0.8755328207975913  0.5838295293767531  0.9260580887126498   T   T   T
  0.1244671792024091  0.5838295293767531  0.9260580887126498   T   T   T
  0.0000000000000000  0.8339659634388443  0.9260762163820175   T   T   T
  0.2500250655072142  0.8331981552934801  0.9260789611409616   T   T   T
  0.0000000000000000  0.1666667040757801  0.8319822799999983   F   F   F
  0.2499999757020674  0.1666667040757801  0.8319822799999983   F   F   F
  0.1250000364468988  0.4166667040757801  0.8319822799999983   F   F   F
  0.3750000121489663  0.4166667040757801  0.8319822799999983   F   F   F
  0.5000000485958651  0.1666667040757801  0.8319822799999983   F   F   F
  0.7500000242979326  0.1666667040757801  0.8319822799999983   F   F   F
  0.6249999878510337  0.4166667040757801  0.8319822799999983   F   F   F
  0.8749999635531012  0.4166667040757801  0.8319822799999983   F   F   F
  0.2499999757020674  0.6666667040757801  0.8319822799999983   F   F   F
  0.5000000485958651  0.6666667040757801  0.8319822799999983   F   F   F
  0.3750000121489663  0.9166667040757801  0.8319822799999983   F   F   F
  0.6249999878510337  0.9166667040757801  0.8319822799999983   F   F   F
  0.7500000242979326  0.6666667040757801  0.8319822799999983   F   F   F
  0.0000000000000000  0.6666667040757801  0.8319822799999983   F   F   F
  0.8749999635531012  0.9166667040757801  0.8319822799999983   F   F   F
  0.1250000364468988  0.9166667040757801  0.8319822799999983   F   F   F
  0.4999999514041349  0.1610503008326323  0.0747633959140340   T   T   T
  0.4999999514041349  0.1606847234267350  0.1247207561953386   T   T   T
```

```
Cu111_CH2
  1.00000000000000
   10.2889409999999994    0.0000000000000000    0.0000000000000000
    0.0000000000000000    8.9104840000000003    0.0000000000000000
    0.0000000000000000    0.0000000000000000   22.0000000000000000
  Cu   C   H
  48     1     2
Selective dynamics
Direct
  0.0000000000000000  0.9995072725545988  0.0204688439728070   T   T   T
  0.1244607358813407  0.2493764494708482  0.0198618490127862   T   T   T
  0.8755392641186595  0.2493764494708482  0.0198618490127862   T   T   T
  0.6262884142827126  0.7468218598844757  0.0190258601679046   T   T   T
  0.2464756157053787  0.9960829102150758  0.0187703338975363   T   T   T
  0.3765840160858289  0.2529137525729508  0.0244936323899192   T   T   T
  0.5000000485958651  0.9909560865693743  0.0243698455199029   T   T   T
  0.7535243842946215  0.9960829102150758  0.0187703338975363   T   T   T
  0.6234159839141709  0.2529137525729508  0.0244936323899192   T   T   T
  0.5000000485958651  0.5016071259871352  0.0202840380494737   T   T   T
  0.2485904665023436  0.4991955075971714  0.0202739573351788   T   T   T
  0.3737115857172870  0.7468218598844757  0.0190258601679046   T   T   T
  0.7514095334976562  0.4991955075971714  0.0202739573351788   T   T   T
  0.0000000000000000  0.4988023337043517  0.0205748382767210   T   T   T
  0.8760799461348572  0.7482028864621678  0.0204943238291274   T   T   T
  0.1239200538651430  0.7482028864621678  0.0204943238291274   T   T   T
  0.1245169049964489  0.0838993309090327  0.9259296299464154   T   T   T
  0.3751500813944798  0.0827245659045588  0.9268533314893602   T   T   T
  0.2524268111744684  0.3307988887851892  0.9274667838771861   T   T   T
  0.5000000485958651  0.3318781087342236  0.9264651395572848   T   T   T
  0.6248499186055202  0.0827245659045588  0.9268533314893602   T   T   T
  0.8754830950035513  0.0838993309090327  0.9259296299464154   T   T   T
  0.7475731888255315  0.3307988887851892  0.9274667838771861   T   T   T
  0.0000000000000000  0.3341432223446453  0.9256522559187886   T   T   T
  0.3752337665044840  0.5810172074758509  0.9259537354897920   T   T   T
  0.6247662334955163  0.5810172074758509  0.9259537354897920   T   T   T
  0.5000000485958651  0.8353603795399779  0.9263831969216042   T   T   T
  0.7500652110595186  0.8324409503144042  0.9256481996022687   T   T   T
  0.8749588647286564  0.5829505287806296  0.9259648311382971   T   T   T
  0.1250411352713434  0.5829505287806296  0.9259648311382971   T   T   T
  0.0000000000000000  0.8336939714897595  0.9260815441313124   T   T   T
  0.2499347889404812  0.8324409503144042  0.9256481996022687   T   T   T
  0.0000000000000000  0.1666667040757801  0.8319822799999983   F   F   F
  0.2499999757020674  0.1666667040757801  0.8319822799999983   F   F   F
  0.1250000364468988  0.4166667040757801  0.8319822799999983   F   F   F
  0.3750000121489663  0.4166667040757801  0.8319822799999983   F   F   F
  0.5000000485958651  0.1666667040757801  0.8319822799999983   F   F   F
  0.7500000242979326  0.1666667040757801  0.8319822799999983   F   F   F
  0.6249999878510337  0.4166667040757801  0.8319822799999983   F   F   F
  0.8749999635531012  0.4166667040757801  0.8319822799999983   F   F   F
  0.2499999757020674  0.6666667040757801  0.8319822799999983   F   F   F
  0.5000000485958651  0.6666667040757801  0.8319822799999983   F   F   F
  0.3750000121489663  0.9166667040757801  0.8319822799999983   F   F   F
  0.6249999878510337  0.9166667040757801  0.8319822799999983   F   F   F
  0.7500000242979326  0.6666667040757801  0.8319822799999983   F   F   F
  0.0000000000000000  0.6666667040757801  0.8319822799999983   F   F   F
  0.8749999635531012  0.9166667040757801  0.8319822799999983   F   F   F
  0.1250000364468988  0.9166667040757801  0.8319822799999983   F   F   F
  0.4999999514041349  0.1528801132316776  0.0853798368140914   T   T   T
  0.5856284481095759  0.1602701379240093  0.1157641288724292   T   T   T
  0.4143714546986937  0.1602701379240093  0.1157641288724292   T   T   T
```

```
Cu111_CH3
  1.00000000000000
   10.2889409999999994    0.0000000000000000    0.0000000000000000
    0.0000000000000000    8.9104840000000003    0.0000000000000000
    0.0000000000000000    0.0000000000000000   22.0000000000000000
  Cu   C   H
  48     1     3
Selective dynamics
Direct
  0.0000582986809243  0.9977967457408355  0.9971938641746619   T   T   T
  0.1245183325814053  0.2478656473404963  0.9970230350610791   T   T   T
  0.8756469467592097  0.2478762403375149  0.9969911955612399   T   T   T
  0.6262472521927755  0.7487750437282882  0.9965748585845823   T   T   T
  0.2464381015593576  0.9963379633063212  0.9954651254593678   T   T   T
  0.3763425857621460  0.2469460768322355  0.0006922222066700   T   T   T
  0.5000809465921913 -0.0008208245354887 -0.0007085310519861   T   T   T
  0.7537005749527161  0.9963094194954598  0.9954268125264789   T   T   T
  0.6238413967475521  0.2469589423402572  0.0006404769719563   T   T   T
  0.5000237699419965  0.5023931177016669  0.9956940384956672   T   T   T
  0.2500971216408677  0.4983601800757649  0.9969682820324771   T   T   T
  0.3738211810734510  0.7488071324565696  0.9965807051718633   T   T   T
  0.7499906922105939  0.4983764629805614  0.9969445080877901   T   T   T
  0.0000429790624318  0.4974134261791642  0.9972009430298290   T   T   T
  0.8756041689152095  0.7469612286399753  0.9972230242098352   T   T   T
  0.1244622867907895  0.7469678352174691  0.9972216653130579   T   T   T
  0.1243651417035812  0.0827064810439773  0.9027125997538844   T   T   T
  0.3748964576046850  0.0822927525985458  0.9030393952910769   T   T   T
  0.2526891647690523  0.3303808858175723  0.9048492855739102   T   T   T
  0.5000152157289453  0.3319422191926708  0.9031900433553235   T   T   T
  0.6251681654297669  0.0822780655731856  0.9030112069778672   T   T   T
  0.8756920543379223  0.0827118988088856  0.9027008218602823   T   T   T
  0.7473724159888678  0.3303906282299002  0.9048247545375813   T   T   T
  0.0000489509570961  0.3328166761581490  0.9026577942553842   T   T   T
  0.3747259535080167  0.5821906234867598  0.9026903288551218   T   T   T
  0.6252797864997630  0.5821724179571235  0.9026863285735330   T   T   T
  0.5000114613162255  0.8349948296492611  0.9044714472154886   T   T   T
  0.7502571320986950  0.8314940309248601  0.9026154257219982   T   T   T
  0.8751628970805398  0.5820195491162601  0.9028711185483131   T   T   T
  0.1248856719172488  0.5820186895039544  0.9028752848541591   T   T   T
  0.0000113409675082  0.8324428345691229  0.9030356801478672   T   T   T
  0.2497586963010015  0.8315230364605084  0.9026211611496820   T   T   T
  0.0000000000000000  0.1666667040757801  0.8090707727272743   F   F   F
  0.2499999757020674  0.1666667040757801  0.8090707727272743   F   F   F
  0.1250000364468988  0.4166667040757801  0.8090707727272743   F   F   F
  0.3750000121489663  0.4166667040757801  0.8090707727272743   F   F   F
  0.5000000485958651  0.1666667040757801  0.8090707727272743   F   F   F
  0.7500000242979326  0.1666667040757801  0.8090707727272743   F   F   F
  0.6249999878510337  0.4166667040757801  0.8090707727272743   F   F   F
  0.8749999635531012  0.4166667040757801  0.8090707727272743   F   F   F
  0.2499999757020674  0.6666667040757801  0.8090707727272743   F   F   F
  0.5000000485958651  0.6666667040757801  0.8090707727272743   F   F   F
  0.3750000121489663  0.9166667040757801  0.8090707727272743   F   F   F
  0.6249999878510337  0.9166667040757801  0.8090707727272743   F   F   F
  0.7500000242979326  0.6666667040757801  0.8090707727272743   F   F   F
  0.0000000000000000  0.6666667040757801  0.8090707727272743   F   F   F
  0.8749999635531012  0.9166667040757801  0.8090707727272743   F   F   F
  0.1250000364468988  0.9166667040757801  0.8090707727272743   F   F   F
  0.5005656880854648  0.1571303526083457  0.0762564532052892   T   T   T
  0.4145846880154295  0.2114808887640211  0.0968747792142578   T   T   T
  0.5866211319433715  0.2116398870366843  0.0967541481190399   T   T   T
  0.5007509882698452  0.0404520725069447  0.0937088472134143   T   T   T
```

```
Cu_CO
  1.00000000000000
   10.2889409999999994    0.0000000000000000    0.0000000000000000
    0.0000000000000000    8.9104840000000003    0.0000000000000000
    0.0000000000000000    0.0000000000000000   22.0000000000000000
  Cu   C   O
  48     1     1
Selective dynamics
Direct
 -0.0023869223303258 -0.0009778891212649  0.0192163013307531   T   T   T
  0.1238753009401366  0.2505132936917567  0.0227958004934774   T   T   T
  0.8743314289436911  0.2504818954892508  0.0200924811848473   T   T   T
  0.6255110829068692  0.7492959750424207  0.0200977331552772   T   T   T
  0.2500006560441932 -0.0007943951203265  0.0230523414081489   T   T   T
  0.3761262055317103  0.2505132936917567  0.0227958004934774   T   T   T
  0.5023883316104425 -0.0009778891212649  0.0192163013307531   T   T   T
  0.7500020653243098 -0.0001842045468335  0.0198282057637569   T   T   T
  0.6256727017049285  0.2504818954892508  0.0200924811848473   T   T   T
  0.5002527331826492  0.5007679687856641  0.0197851201556047   T   T   T
  0.2500006560441932  0.5028398806621016  0.0185244516897553   T   T   T
  0.3756996022392383  0.7507056725739549  0.0196210575030096   T   T   T
  0.7500020653243098  0.4998806291680025  0.0206176543524125   T   T   T
 -0.0002513239025320  0.5007679687856641  0.0197851201556047   T   T   T
  0.8744930477417504  0.7492959750424207  0.0200977331552772   T   T   T
  0.1243019042326086  0.7507056725739549  0.0196210575030096   T   T   T
  0.1251491684854560  0.0833665473926814  0.9256844748680761   T   T   T
  0.3748523379863908  0.0833665473926814  0.9256844748680761   T   T   T
  0.2500006560441932  0.3329792006613908  0.9258308148470934   T   T   T
  0.4989462421212799  0.3326416488899926  0.9267437575525921   T   T   T
  0.6253535168531283  0.0840740168547428  0.9258195881168221   T   T   T
  0.8746506137954912  0.0840740168547428  0.9258195881168221   T   T   T
  0.7500020653243098  0.3339857786196311  0.9259400206723225   T   T   T
  0.0010551671588369  0.3326416488899926  0.9267437575525921   T   T   T
  0.3754639674304747  0.5840656040804711  0.9255816330768032   T   T   T
  0.6255994737920740  0.5831943264183037  0.9259145944167125   T   T   T
  0.5011337381705806  0.8332123197355170  0.9258223817633691   T   T   T
  0.7500020653243098  0.8336657395478588  0.9261112100919595   T   T   T
  0.8744046568565456  0.5831943264183037  0.9259145944167125   T   T   T
  0.1245375390413722  0.5840656040804711  0.9255816330768032   T   T   T
 -0.0011323288904637  0.8332123197355170  0.9258223817633691   T   T   T
  0.2500006560441932  0.8355147624760617  0.9269231710719148   T   T   T
  0.0000000000000000  0.1666671529851769  0.8319822727272737   F   F   F
  0.2500006560441932  0.1666671529851769  0.8319822727272737   F   F   F
  0.1250004252138268  0.4166678263492756  0.8319822727272737   F   F   F
  0.3750010812580200  0.4166678263492756  0.8319822727272737   F   F   F
  0.5000014092801166  0.1666671529851769  0.8319822727272737   F   F   F
  0.7500020653243098  0.1666671529851769  0.8319822727272737   F   F   F
  0.6250017373022132  0.4166678263492756  0.8319822727272737   F   F   F
  0.8750023933464064  0.4166678263492756  0.8319822727272737   F   F   F
  0.2500006560441932  0.6666686119407217  0.8319822727272737   F   F   F
  0.5000014092801166  0.6666686119407217  0.8319822727272737   F   F   F
  0.3750010812580200  0.9166692853048133  0.8319822727272737   F   F   F
  0.6250017373022132  0.9166692853048133  0.8319822727272737   F   F   F
  0.7500020653243098  0.6666686119407217  0.8319822727272737   F   F   F
  0.0000000000000000  0.6666686119407217  0.8319822727272737   F   F   F
  0.8750023933464064  0.9166692853048133  0.8319822727272737   F   F   F
  0.1250004252138268  0.9166692853048133  0.8319822727272737   F   F   F
  0.2500006560441932  0.1651731753909975  0.0859312702517412   T   T   T
  0.2500006560441932  0.1685269403740754  0.1397375526813032   T   T   T
```

```
Cu_COOH
  1.00000000000000
   10.2889689999999998    0.0000000000000000    0.0000000000000000
    0.0000000000000000    8.9105089999999993    0.0000000000000000
    0.0000000000000000    0.0000000000000000   22.0000000000000000
  Cu   C   O   H
  48     1     2     1
Selective dynamics
Direct
 -0.0026434498380861 -0.0043004560775275  0.0242426926429479   T   T   T
  0.1295781804700766  0.2573882626339492  0.0250482926358825   T   T   T
  0.8718369229175325  0.2516358518563478  0.0194624499754735   T   T   T
  0.6242567791132198  0.7504151233287384  0.0203041646923773   T   T   T
  0.2537988792040502 -0.0023070972721331  0.0190521075339556   T   T   T
  0.3786848169651918  0.2509637739727285  0.0199820134639334   T   T   T
  0.5006301596767433  0.0005670081231243  0.0202586095688130   T   T   T
  0.7475456206471813  0.0000246598493388  0.0198366389159795   T   T   T
  0.6249967271796402  0.2510003464543714  0.0203524073675342   T   T   T
  0.5006921373803818  0.5010794692836384  0.0203070568898880   T   T   T
  0.2522043893700576  0.5038452639107303  0.0199467852354074   T   T   T
  0.3764331723552843  0.7503877600424526  0.0200578939499750   T   T   T
  0.7493220459051477  0.5002898223659420  0.0202157115816793   T   T   T
 -0.0005016006004509  0.5029323734559192  0.0195519367968874   T   T   T
  0.8725406596803998  0.7476170050662511  0.0195979992029616   T   T   T
  0.1260271254346411  0.7492007042358442  0.0195915246907808   T   T   T
  0.1241708627978333  0.0839866825994819  0.9276623191032561   T   T   T
  0.3749114446148132  0.0843787542612427  0.9260695400147361   T   T   T
  0.2484789826146249  0.3323716261539883  0.9276265168221708   T   T   T
  0.5002034849610625  0.3345049988202362  0.9257032104318529   T   T   T
  0.6249356298322720  0.0844107815650369  0.9258903813563847   T   T   T
  0.8765302637120561  0.0821104040115995  0.9267584834062391   T   T   T
  0.7504411594469887  0.3335957598415477  0.9258681702073048   T   T   T
  0.0014897897769658  0.3316026625924493  0.9275680412842131   T   T   T
  0.3751919980252098  0.5839324767609860  0.9257584973575440   T   T   T
  0.6250014455736604  0.5840067182954106  0.9259692907300620   T   T   T
  0.5000367585304383  0.8341578872006995  0.9259262585837812   T   T   T
  0.7498343834383847  0.8337017500632425  0.9260501938600293   T   T   T
  0.8751804975000751  0.5830164992156356  0.9257161033842407   T   T   T
  0.1249059971651037  0.5834210987395891  0.9258645465657339   T   T   T
 -0.0002984639500122  0.8356210834748034  0.9268784860348299   T   T   T
  0.2494938039191034  0.8337708148260758  0.9257498504371487   T   T   T
  0.0000000000000000  0.1666666853711689  0.8319822727272737   F   F   F
  0.2499999757021314  0.1666666853711689  0.8319822727272737   F   F   F
  0.1250000850425366  0.4166666573144155  0.8319822727272737   F   F   F
  0.3750000607446680  0.4166666573144155  0.8319822727272737   F   F   F
  0.5000000485957372  0.1666666853711689  0.8319822727272737   F   F   F
  0.7500000242978686  0.1666666853711689  0.8319822727272737   F   F   F
  0.6250000364467994  0.4166666573144155  0.8319822727272737   F   F   F
  0.8750000121489308  0.4166666573144155  0.8319822727272737   F   F   F
  0.2499999757021314  0.6666667414846899  0.8319822727272737   F   F   F
  0.5000000485957372  0.6666667414846899  0.8319822727272737   F   F   F
  0.3750000607446680  0.9166667134279294  0.8319822727272737   F   F   F
  0.6250000364467994  0.9166667134279294  0.8319822727272737   F   F   F
  0.7500000242978686  0.6666667414846899  0.8319822727272737   F   F   F
  0.0000000000000000  0.6666667414846899  0.8319822727272737   F   F   F
  0.8750000121489308  0.9166667134279294  0.8319822727272737   F   F   F
  0.1250000850425366  0.9166667134279294  0.8319822727272737   F   F   F
  0.0390792076214894  0.0588367988516921  0.1069021881166430   T   T   T
  0.0993049077624076  0.1798347690976827  0.1160412303761128   T   T   T
  0.0059615003141081  0.9750626501129914  0.1555373740130926   T   T   T
  0.0382364871411454  0.0272453932905170  0.1922352293715203   T   T   T
```

```
Cu_COH
  1.00000000000000
   10.2889689999999998    0.0000000000000000    0.0000000000000000
    0.0000000000000000    8.9105089999999993    0.0000000000000000
    0.0000000000000000    0.0000000000000000   22.0000000000000000
  Cu   C   O   H
  48     1     1     1
Selective dynamics
Direct
 -0.0000298071709117 -0.0002634692267328 -0.0025051056643053   T   T   T
  0.1245942346160633  0.2503900314850363 -0.0027721914534111   T   T   T
  0.8760389239587394  0.2489418296210417 -0.0031405311045220   T   T   T
  0.6241684000432267  0.7510384743022690 -0.0038656180186319   T   T   T
  0.2486024170954893  0.0005554953603930 -0.0024719670696223   T   T   T
  0.3733746718992808  0.2496577360643296 -0.0028547647357805   T   T   T
  0.4982299799127695 -0.0020250240930274 -0.0036418299432427   T   T   T
  0.7507962150924231 -0.0028442213563852 -0.0036447282151612   T   T   T
  0.6253014978038085  0.2413502253633600  0.0011964746009389   T   T   T
  0.4957725158808432  0.5049520582452851  0.0012821106194911   T   T   T
  0.2479724146543228  0.5011758709970009 -0.0025336141520557   T   T   T
  0.3726315799604146  0.7516383949891409 -0.0029407229476351   T   T   T
  0.7498687689674001  0.5009665268992506  0.0019222406249499   T   T   T
 -0.0002192170991215  0.5008870768633092 -0.0024969939598988   T   T   T
  0.8749913862993972  0.7496778520319713 -0.0027828523049397   T   T   T
  0.1239977703910000  0.7503963374291555 -0.0018889642158562   T   T   T
  0.1244230201506426  0.0842093394727107  0.9032942422518968   T   T   T
  0.3738531325263721  0.0837783292737949  0.9030832496196211   T   T   T
  0.2496371256188876  0.3342600760407032  0.9031960658308373   T   T   T
  0.5003812987662554  0.3332645529452254  0.9034748437667111   T   T   T
  0.6246814113173643  0.0855640706905086  0.9038816239507190   T   T   T
  0.8751991112743289  0.0835847494369218  0.9030616489433602   T   T   T
  0.7491930831305259  0.3331871821172951  0.9034110832181144   T   T   T
 -0.0002443598840825  0.3343174269603146  0.9031531507687958   T   T   T
  0.3762185267270017  0.5819785191657099  0.9040525059627381   T   T   T
  0.6245645739592065  0.5818598647601902  0.9029085350771888   T   T   T
  0.4992746515069520  0.8324885984652058  0.9028198658803219   T   T   T
  0.7497084646035147  0.8323992081630450  0.9027857972131985   T   T   T
  0.8727056667733036  0.5816652946618871  0.9042963662474353   T   T   T
  0.1244864408377730  0.5846910143506528  0.9031431055218915   T   T   T
 -0.0002307217547870  0.8334013685481483  0.9031961782096428   T   T   T
  0.2489479650513167  0.8335896712308061  0.9030877842657621   T   T   T
  0.0000000000000000  0.1666666853711689  0.8090702727272756   F   F   F
  0.2499999757021314  0.1666666853711689  0.8090702727272756   F   F   F
  0.1249999878510692  0.4166666573144155  0.8090702727272756   F   F   F
  0.3750000607446680  0.4166666573144155  0.8090702727272756   F   F   F
  0.5000000485957372  0.1666666853711689  0.8090702727272756   F   F   F
  0.7500000242978686  0.1666666853711689  0.8090702727272756   F   F   F
  0.6250000364467994  0.4166666573144155  0.8090702727272756   F   F   F
  0.8750000121489308  0.4166666573144155  0.8090702727272756   F   F   F
  0.2499999757021314  0.6666666292576551  0.8090702727272756   F   F   F
  0.5000000485957372  0.6666666292576551  0.8090702727272756   F   F   F
  0.3750000607446680  0.9166666012008946  0.8090702727272756   F   F   F
  0.6250000364467994  0.9166666012008946  0.8090702727272756   F   F   F
  0.7500000242978686  0.6666666292576551  0.8090702727272756   F   F   F
  0.0000000000000000  0.6666666292576551  0.8090702727272756   F   F   F
  0.8750000121489308  0.9166666012008946  0.8090702727272756   F   F   F
```

```
 0.1250000850425366 0.9166666012008946 0.8090702727272756  F  F  F
 0.6187915800383589 0.4076856335179504 0.0559296122459504  T  T  T
 0.6204543330390947 0.4023774528653622 0.1167436427282164  T  T  T
 0.6014373500527472 0.5004909639545627 0.1347887299074546  T  T  T
```

```
Cu_CHO
  1.0000000000000000
   10.2889689999999998    0.0000000000000000    0.0000000000000000
    0.0000000000000000    8.9105089999999993    0.0000000000000000
    0.0000000000000000    0.0000000000000000   22.0000000000000000
  Cu   C   O   H
  48    1    1    1
Selective dynamics
Direct
 0.0011798007191241 0.0019968468019102 -0.0025456786050580  T  T  T
 0.1250514306065156 0.2530710448253208 -0.0024798684250556  T  T  T
 0.8779768330819938 0.2508769881413269 -0.0036903634733160  T  T  T
 0.6250510105125519 0.7573002774475034 -0.0040006577126852  T  T  T
 0.2489134718835032 0.0020471200144234 -0.0025325039260667  T  T  T
 0.3721441443328580 0.2509564338766336 -0.0036892215140571  T  T  T
 0.4972654910167923 -0.0006703349078458 -0.0031002573068348  T  T  T
 0.7528331275702937 -0.0006857059576862 -0.0031050206759528  T  T  T
 0.6251089109293716 0.2402758419010677 0.0017132712268897  T  T  T
 0.5018158098354616 0.5087505500518344 0.0001550023895679  T  T  T
 0.2507460363194415 0.5023837975909843 -0.0029227048991617  T  T  T
 0.3730230631551381 0.7531200830751708 -0.0028171743808132  T  T  T
 0.7481579281894737 0.5088233387396457 0.0001995639906171  T  T  T
-0.0007188086839217 0.5023546934835174 -0.0029056582351926  T  T  T
 0.8769932360744561 0.7530699399897935 -0.0027850201282823  T  T  T
 0.1250050560893012 0.7519925823501377 -0.0025989327572679  T  T  T
 0.1250344320154047 0.0851465016240674 0.9031910923144746  T  T  T
 0.3750153882964288 0.0846788410224242 0.9029850128456408  T  T  T
 0.2498149676596435 0.3353036969102107 0.9029596828530587  T  T  T
 0.5012977493952321 0.3352665242546025 0.9049344298836351  T  T  T
 0.6250333728523635 0.0871896489729375 0.9045401575536958  T  T  T
 0.8750543382456213 0.0846719847759864 0.9029730815628166  T  T  T
 0.7487421675934748 0.3352833716501975 0.9049645765091814  T  T  T
 0.0002135249186456 0.3352921360185692 0.9029685275070641  T  T  T
 0.3762486911688439 0.5834731321189452 0.9038466735374406  T  T  T
 0.6250084901649950 0.5850534044957392 0.9020623840549920  T  T  T
 0.4997348422444944 0.8343982472991197 0.9027467846210000  T  T  T
 0.7503168354801899 0.8344128634628882 0.9027404276486103  T  T  T
 0.8737600564295878 0.5834428726756823 0.9038656729539335  T  T  T
 0.1249839700860075 0.5853489683356932 0.9029419036579348  T  T  T
 0.0000498223498256 0.8346813175844719 0.9029599712748804  T  T  T
 0.2499471475602561 0.8347001926281188 0.9029637291656191  T  T  T
 0.0000000000000000 0.1666666853711689 0.8090702727272756  F  F  F
 0.2499999757021314 0.1666666853711689 0.8090702727272756  F  F  F
 0.1249999878510692 0.4166666573144155 0.8090702727272756  F  F  F
 0.3750000607446680 0.4166666573144155 0.8090702727272756  F  F  F
 0.5000000485957372 0.1666666853711689 0.8090702727272756  F  F  F
 0.7500000242978686 0.1666666853711689 0.8090702727272756  F  F  F
 0.6250000364467994 0.4166666573144155 0.8090702727272756  F  F  F
 0.8750000121489308 0.4166666573144155 0.8090702727272756  F  F  F
 0.2499999757021314 0.6666666292576551 0.8090702727272756  F  F  F
 0.5000000485957372 0.6666666292576551 0.8090702727272756  F  F  F
 0.3750000607446680 0.9166666012008946 0.8090702727272756  F  F  F
 0.6250000364467994 0.9166666012008946 0.8090702727272756  F  F  F
 0.7500000242978686 0.6666666292576551 0.8090702727272756  F  F  F
 0.0000000000000000 0.6666666292576551 0.8090702727272756  F  F  F
 0.8750000121489308 0.9166666012008946 0.8090702727272756  F  F  F
 0.1250000850425366 0.9166666012008946 0.8090702727272756  F  F  F
 0.6243904686337398 0.4761634965245785 0.0734445339941083  T  T  T
 0.6254046557677848 0.3398918454365405 0.0898081077362476  T  T  T
 0.6241595739858321 0.5603558486264368 0.1108530215206557  T  T  T
```

```
Cu111_C_Na
  1.00000000000000
   10.2889409999999994    0.0000000000000000    0.0000000000000000
    0.0000000000000000    8.9104840000000003    0.0000000000000000
    0.0000000000000000    0.0000000000000000   22.0000000000000000
  Cu   C   Na
  48    1    1
Selective dynamics
Direct
 0.0000000000000000 -0.0003377814527071 -0.0025755545501512  T  T  T
 0.1231629253132924 0.2504577480879673 -0.0025033021987382  T  T  T
 0.8768370746867079 0.2504577480879673 -0.0025033021987382  T  T  T
 0.6259125938473307 0.7454139794230071 -0.0036549626145535  T  T  T
 0.2476078660694067 -0.0029772830119429 -0.0023162428619936  T  T  T
 0.3686437502434388 0.2511234578780063 0.0011277613687902  T  T  T
 0.5000000485958651 -0.0110745463249114 0.0011995749194408  T  T  T
 0.7523921339305933 -0.0029772830119429 -0.0023162428619936  T  T  T
 0.6313562497565616 0.2511234578780063 0.0011277613687902  T  T  T
 0.5000000485958651 0.5008723110906823 -0.0057697525725747  T  T  T
 0.2489960549451951 0.4998742528327456 -0.0029047783151042  T  T  T
 0.3740874061526693 0.7454139794230071 -0.0036549626145535  T  T  T
 0.7510039450548049 0.4998742528327456 -0.0029047783151042  T  T  T
 0.0000000000000000 0.5003820739533293 -0.0024224901241010  T  T  T
 0.8756893540191781 0.7494178557956842 -0.0026315523488226  T  T  T
 0.1243106459808219 0.7494178557956842 -0.0026315523488226  T  T  T
 0.1252379500206910 0.0842111209822834 0.9032440560343989  T  T  T
 0.3752814667104794 0.0819467103436451 0.9041953539450751  T  T  T
 0.2505326810397632 0.3318351250330472 0.9035434454584663  T  T  T
 0.5000000485958651 0.3296956118316468 0.9032978360287637  T  T  T
 0.6247185332895208 0.0819467103436451 0.9041953539450751  T  T  T
 0.8747620499793088 0.0842111209822834 0.9032440560343989  T  T  T
 0.7494673189602364 0.3318351250330472 0.9035434454584663  T  T  T
 0.0000000000000000 0.3345967776745477 0.9029929273844945  T  T  T
 0.3743019094352480 0.5809671363726499 0.9020324472532608  T  T  T
 0.6256980905647521 0.5809671363726499 0.9020324472532608  T  T  T
 0.5000000485958651 0.8329774634039057 0.9033201239897526  T  T  T
 0.7500677649395353 0.8334680352921997 0.9030902682391000  T  T  T
 0.8753773948167450 0.5838223332209738 0.9029545903079678  T  T  T
 0.1246226051832549 0.5838223332209738 0.9029545903079678  T  T  T
 0.0000000000000000 0.8343864050481378 0.9031226543120363  T  T  T
 0.2499322350604652 0.8334680352921997 0.9030902682391000  T  T  T
 0.0000000000000000 0.1666667040757801 0.8090707727272743  F  F  F
 0.2499999757020674 0.1666667040757801 0.8090707727272743  F  F  F
 0.1250000364468988 0.4166667040757801 0.8090707727272743  F  F  F
 0.3750000121489663 0.4166667040757801 0.8090707727272743  F  F  F
 0.5000000485958651 0.1666667040757801 0.8090707727272743  F  F  F
 0.7500000242979326 0.1666667040757801 0.8090707727272743  F  F  F
 0.6249999878510337 0.4166667040757801 0.8090707727272743  F  F  F
 0.8749999635531012 0.4166667040757801 0.8090707727272743  F  F  F
 0.2499999757020674 0.6666667040757801 0.8090707727272743  F  F  F
 0.5000000485958651 0.6666667040757801 0.8090707727272743  F  F  F
 0.3750000121489663 0.9166667040757801 0.8090707727272743  F  F  F
 0.6249999878510337 0.9166667040757801 0.8090707727272743  F  F  F
 0.7500000242979326 0.6666667040757801 0.8090707727272743  F  F  F
 0.0000000000000000 0.6666667040757801 0.8090707727272743  F  F  F
 0.8749999635531012 0.9166667040757801 0.8090707727272743  F  F  F
 0.1250000364468988 0.9166667040757801 0.8090707727272743  F  F  F
 0.4999999514041349 0.1618749042937011 0.0491144587300836  T  T  T
 0.5000000485958651 0.3785853644139746 0.1146075866534982  T  T  T
```

```
Cu111_CH_Na
  1.00000000000000
   10.2889409999999994    0.0000000000000000    0.0000000000000000
    0.0000000000000000    8.9104840000000003    0.0000000000000000
    0.0000000000000000    0.0000000000000000   22.0000000000000000
  Cu   C   H   Na
  48    1    1    1
Selective dynamics
Direct
 0.0009710528667795 0.0011841619858092 -0.0012466438071970  T  T  T
 0.1243039394795032 0.2496722976546509 -0.0023174610881218  T  T  T
 0.8778587998886918 0.2513603616879290 -0.0021911217637007  T  T  T
 0.6255650918036708 0.7477101824130901 -0.0032595454733455  T  T  T
 0.2465911423828639 -0.0041580744253583 -0.0062794117199814  T  T  T
 0.3716541990486826 0.2541393061183849 0.0019005781147840  T  T  T
 0.4992782624546199 -0.0067885260199475 0.0028621113557736  T  T  T
 0.7516784492030154 -0.0009344427829300 -0.0023999291832776  T  T  T
 0.6308943951638228 0.2529298412540345 0.0012317536750232  T  T  T
 0.5008217685561098 0.5003518053622633 -0.0030471413998716  T  T  T
 0.2482593837359115 0.5004960800720499 -0.0024957696059193  T  T  T
 0.3735510722469274 0.7476018244815882 -0.0034907482028943  T  T  T
 0.7525957142027750 0.5015957794586196 -0.0029610765284040  T  T  T
 0.0005312559698770 0.5006030261011227 -0.0017692816232055  T  T  T
 0.8759378481065496 0.7513641814651643 -0.0020978165630455  T  T  T
 0.1243753203109584 0.7501384688611581 -0.0017593897066173  T  T  T
 0.1243252334781783 0.0847187325281691 0.9023046208445787  T  T  T
 0.3757519111319348 0.0831575953598714 0.9027607913455479  T  T  T
 0.2521117052750276 0.3314437368684159 0.9041941112083984  T  T  T
 0.5004815782582073 0.3318772085740160 0.9034682088772107  T  T  T
 0.6245174115148586 0.0831239607685587 0.9040030380296820  T  T  T
 0.8746555087844501 0.0843022517202502 0.9036975693673099  T  T  T
 0.7492783104320562 0.3325252917107839 0.9042301873119782  T  T  T
 0.0003534132827222 0.3353242989732609 0.9030447904463302  T  T  T
 0.3751142625915929 0.5810412080150364 0.9031443037917438  T  T  T
 0.6251380826251347 0.5820239573401279 0.9030517507027074  T  T  T
 0.4998622034992563 0.8357724525080452 0.9044036774857640  T  T  T
 0.7499945880045100 0.8338479198642170 0.9033417838760072  T  T  T
 0.8758282380174384 0.5848603019794787 0.9031359569088172  T  T  T
 0.1246597247187374 0.5837616633095896 0.9033468279414781  T  T  T
 0.0002128290682482 0.8351537753277056 0.9036727935030042  T  T  T
 0.2499446408818661 0.8320275499342789 0.9023783508056886  T  T  T
 0.0000000000000000 0.1666667040757801 0.8090707727272743  F  F  F
 0.2499999757020674 0.1666667040757801 0.8090707727272743  F  F  F
 0.1250000364468988 0.4166667040757801 0.8090707727272743  F  F  F
 0.3750000121489663 0.4166667040757801 0.8090707727272743  F  F  F
 0.5000000485958651 0.1666667040757801 0.8090707727272743  F  F  F
 0.7500000242979326 0.1666667040757801 0.8090707727272743  F  F  F
 0.6249999878510337 0.4166667040757801 0.8090707727272743  F  F  F
 0.8749999635531012 0.4166667040757801 0.8090707727272743  F  F  F
 0.2499999757020674 0.6666667040757801 0.8090707727272743  F  F  F
 0.5000000485958651 0.6666667040757801 0.8090707727272743  F  F  F
 0.3750000121489663 0.9166667040757801 0.8090707727272743  F  F  F
 0.6249999878510337 0.9166667040757801 0.8090707727272743  F  F  F
 0.7500000242979326 0.6666667040757801 0.8090707727272743  F  F  F
 0.0000000000000000 0.6666667040757801 0.8090707727272743  F  F  F
 0.8749999635531012 0.9166667040757801 0.8090707727272743  F  F  F
 0.1250000364468988 0.9166667040757801 0.8090707727272743  F  F  F
 0.5012200505039754 0.1680341634971108 0.0545738915988053  T  T  T
 0.5258760587699282 0.1752141212940815 0.1034070683173979  T  T  T
 0.2782271395285577 0.1191441241794208 0.1092969809283051  T  T  T
```

```
Cu111_CH2_Na
  1.00000000000000
   10.2889409999999994    0.0000000000000000    0.0000000000000000
    0.0000000000000000    8.9104840000000003    0.0000000000000000
    0.0000000000000000    0.0000000000000000   22.0000000000000000
  Cu   C   H   Na
  48    1    2    1
Selective dynamics
Direct
 0.0000000000000000 0.0004423110473173 -0.0020289843419120  T  T  T
 0.1248022860582389 0.2505458968489415 -0.0023378556849337  T  T  T
 0.8751977139417612 0.2505458968489415 -0.0023378556849337  T  T  T
 0.6265622049066447 0.7474809377988845 -0.0029823266120551  T  T  T
 0.2465276313840738 -0.0026902736550947 -0.0037388324452368  T  T  T
 0.3778481450506718 0.2541653683547070 0.0015294515076705  T  T  T
 0.5000000485958651 -0.0080739597091148 0.0018302025286597  T  T  T
 0.7534723686159261 -0.0026902736550947 -0.0037388324452368  T  T  T
 0.6221518549493283 0.2541653683547070 0.0015294515076705  T  T  T
 0.5000000485958651 0.5064727285879410 -0.0094608906115776  T  T  T
 0.2500895874701228 0.5004129652476573 -0.0022217097762661  T  T  T
 0.3734377950933555 0.7474809377988845 -0.0029823266120551  T  T  T
 0.7499104125298771 0.5004129652476573 -0.0022217097762661  T  T  T
 0.0000000000000000 0.4997248952476236 -0.0020564069760523  T  T  T
 0.8759630657770534 0.7484404185869813 -0.0020624683608926  T  T  T
 0.1240369342229464 0.7484404185869813 -0.0020624683608926  T  T  T
 0.1245061795425546 0.0844169249977495 0.9032673627995931  T  T  T
 0.3750921200078168 0.0833621827322018 0.9043574670197915  T  T  T
 0.2524131888843320 0.3314610302275435 0.9049783405685982  T  T  T
 0.5000000485958651 0.3318625015668515 0.9015863479519310  T  T  T
 0.6249078799921832 0.0833621827322018 0.9043574670197915  T  T  T
 0.8754938204574455 0.0844169249977495 0.9032673627995931  T  T  T
 0.7475868111156685 0.3314610302275435 0.9049783405685982  T  T  T
 0.0000000000000000 0.3342170364795174 0.9030800540955951  T  T  T
 0.3728928295212118 0.5836687377062891 0.9011804858270986  T  T  T
 0.6271071704787881 0.5836687377062891 0.9011804858270986  T  T  T
 0.5000000485958651 0.8357206328000714 0.9042838972554547  T  T  T
 0.7502175452177888 0.8334017142292693 0.9031299986935573  T  T  T
 0.8754607372109993 0.5831310197644333 0.9031861323821271  T  T  T
 0.1245392627890008 0.5831310197644333 0.9031861323821271  T  T  T
 0.0000000000000000 0.8338430094973129 0.9033290961535100  T  T  T
 0.2497824547822111 0.8334017142292693 0.9031299986935573  T  T  T
 0.0000000000000000 0.1666667040757801 0.8090707727272743  F  F  F
 0.2499999757020674 0.1666667040757801 0.8090707727272743  F  F  F
 0.1250000364468988 0.4166667040757801 0.8090707727272743  F  F  F
 0.3750000121489663 0.4166667040757801 0.8090707727272743  F  F  F
 0.5000000485958651 0.1666667040757801 0.8090707727272743  F  F  F
 0.7500000242979326 0.1666667040757801 0.8090707727272743  F  F  F
 0.6249999878510337 0.4166667040757801 0.8090707727272743  F  F  F
 0.8749999635531012 0.4166667040757801 0.8090707727272743  F  F  F
 0.2499999757020674 0.6666667040757801 0.8090707727272743  F  F  F
 0.5000000485958651 0.6666667040757801 0.8090707727272743  F  F  F
 0.3750000121489663 0.9166667040757801 0.8090707727272743  F  F  F
 0.6249999878510337 0.9166667040757801 0.8090707727272743  F  F  F
 0.7500000242979326 0.6666667040757801 0.8090707727272743  F  F  F
 0.0000000000000000 0.6666667040757801 0.8090707727272743  F  F  F
 0.8749999635531012 0.9166667040757801 0.8090707727272743  F  F  F
 0.1250000364468988 0.9166667040757801 0.8090707727272743  F  F  F
 0.4999999514041349 0.1507520904463491 0.0657591220805246  T  T  T
 0.5863902535938614 0.1420562043838089 0.0956258507640476  T  T  T
 0.4136096492144014 0.1420562043838089 0.0956258507640476  T  T  T
 0.5000000485958651 0.4306346234375043 0.1077894212880096  T  T  T
```

```
Cu111_CH3_Na
  1.00000000000000
   10.2889409999999994    0.0000000000000000    0.0000000000000000
    0.0000000000000000    8.9104840000000003    0.0000000000000000
    0.0000000000000000    0.0000000000000000   22.0000000000000000
  Cu   C   H   Na
  48    1    3    1
Selective dynamics
Direct
 0.0000360355360297 -0.0001394376845402 -0.0015554886986417  T  T  T
 0.1246763831571708 0.2500247199118820 -0.0020082157120082  T  T  T
 0.8753693016042557 0.2500016538159716 -0.0020180406423279  T  T  T
 0.6258446531299300 0.7487356031122497 -0.0022755995697505  T  T  T
 0.2464295390127757 -0.0023770018305061 -0.0035378648354331  T  T  T
 0.3757035756234707 0.2502018366167863 0.0001328779401347  T  T  T
 0.5000592812089076 0.0038781580703481 0.0018092141445203  T  T  T
 0.7536763635804169 -0.0024510650173519 -0.0035631777018209  T  T  T
 0.6241505308524570 0.2500034555055657 0.0002857010863448  T  T  T
 0.5000092595392599 0.5043075238553820 -0.0101341979338973  T  T  T
 0.2518581704626209 0.4997047024003988 -0.0019023993427139  T  T  T
 0.3742114173929579 0.7488127201646391 -0.0022698489731203  T  T  T
 0.7481564479530498 0.4996244653861577 -0.0019101668476485  T  T  T
 0.0000108588287464 0.4992516102485930 -0.0020957093095925  T  T  T
 0.8754171536454357 0.7479087101612669 -0.0018898723080596  T  T  T
 0.1246240637042911 0.7479450872312059 -0.0018885432639546  T  T  T
 0.1243435312352592 0.0839355509497273 0.9034006573517561  T  T  T
 0.3749166345668201 0.0823822661233526 0.9037670778179415  T  T  T
 0.2515645853386272 0.3319103340635833 0.9045471578971531  T  T  T
 0.5000219215872980 0.3303380249582724 0.9020930001387328  T  T  T
 0.6251265664724299 0.0823881706823745 0.9037625535583357  T  T  T
 0.8756694165891497 0.0839340539348273 0.9033962600318356  T  T  T
 0.7483831688100604 0.3318476886540503 0.9045966919178374  T  T  T
-0.0000000071101334 0.3336852641796247 0.9032681269733503  T  T  T
 0.3731117452019292 0.5849373434885847 0.9010256378986019  T  T  T
 0.6269068592041310 0.5849173488533125 0.9010258919381522  T  T  T
 0.5000210132258999 0.8359902078560051 0.9056036294345110  T  T  T
 0.7505806866814269 0.8328799916659402 0.9033163309961849  T  T  T
 0.8752862186097048 0.5828611022413217 0.9034029409054589  T  T  T
 0.1247251430293769 0.5828823329349493 0.9034039344780085  T  T  T
 0.0000092253307614 0.8337500773900357 0.9037088657885894  T  T  T
 0.2494363029348095 0.8329030923604500 0.9033186715584851  T  T  T
 0.0000000000000000 0.1666667040757801 0.8090707727272743  F  F  F
 0.2499999757020674 0.1666667040757801 0.8090707727272743  F  F  F
 0.1250000364468988 0.4166667040757801 0.8090707727272743  F  F  F
 0.3750000121489663 0.4166667040757801 0.8090707727272743  F  F  F
 0.5000000485958651 0.1666667040757801 0.8090707727272743  F  F  F
 0.7500000242979326 0.1666667040757801 0.8090707727272743  F  F  F
 0.6249999878510337 0.4166667040757801 0.8090707727272743  F  F  F
 0.8749999635531012 0.4166667040757801 0.8090707727272743  F  F  F
 0.2499999757020674 0.6666667040757801 0.8090707727272743  F  F  F
 0.5000000485958651 0.6666667040757801 0.8090707727272743  F  F  F
 0.3750000121489663 0.9166667040757801 0.8090707727272743  F  F  F
 0.6249999878510337 0.9166667040757801 0.8090707727272743  F  F  F
 0.7500000242979326 0.6666667040757801 0.8090707727272743  F  F  F
 0.0000000000000000 0.6666667040757801 0.8090707727272743  F  F  F
 0.8749999635531012 0.9166667040757801 0.8090707727272743  F  F  F
 0.1250000364468988 0.9166667040757801 0.8090707727272743  F  F  F
 0.5009688973220795 0.1386191762437269 0.0829773946353712  T  T  T
 0.4127427837204021 0.1939641395561900 0.1011626994088000  T  T  T
 0.5890241290111323 0.1943800381513245 0.1011573515708923  T  T  T
 0.5012466630164135 0.0244871376492916 0.1028728451720477  T  T  T
 0.4997252789645017 0.4747700548618784 0.1073719073520757  T  T  T
```

```
Cu_CO_Na
  1.00000000000000
   10.2889689999999998    0.0000000000000000    0.0000000000000000
    0.0000000000000000    8.9105089999999993    0.0000000000000000
    0.0000000000000000    0.0000000000000000   22.0000000000000000
  Cu   C   O   Na
  48    1    1    1
Selective dynamics
Direct
-0.0036630743610900 -0.0017526856589459 -0.0028292994308353  T  T  T
 0.1224111456679282 0.2497119448657547 0.0022645287780361  T  T  T
 0.8735770682879743 0.2494771374272768 -0.0017565868100238  T  T  T
 0.6244127772233764 0.7487581597436717 -0.0021191921028494  T  T  T
 0.2492895095583378 -0.0015493367943536 0.0011001230419753  T  T  T
 0.3762772526224131 0.2507181165538716 -0.0020682719214584  T  T  T
 0.5019277035840506 -0.0020607748209876 -0.0045290759770880  T  T  T
 0.7480310012894786 -0.0001225572011061 -0.0017373337864405  T  T  T
 0.6252842719621560 0.2494034320689378 -0.0030233098574369  T  T  T
 0.5000000891133392 0.4990810558338725 -0.0025339732343924  T  T  T
 0.2492163286131109 0.5023813793308608 -0.0037642118049124  T  T  T
 0.3752173209960682 0.7498347782558752 -0.0029223904920320  T  T  T
 0.7493180521753403 0.4988290989157256 -0.0016795303479191  T  T  T
-0.0009946218123566 0.4998353325369089 -0.0025246994535529  T  T  T
 0.8736023887122752 0.7488451738797957 -0.0023966578494177  T  T  T
 0.1237265018632136 0.7500263273155997 -0.0027992658847025  T  T  T
 0.1248839587618839 0.0831528807209017 0.9033602090134524  T  T  T
 0.3739689232213081 0.0828090358869771 0.9023133785254359  T  T  T
 0.2489252034759397 0.3326372618408693 0.9030102381119270  T  T  T
 0.4990485919590475 0.3326527988674050 0.9030723388350994  T  T  T
 0.6253004352316238 0.0839366345679085 0.9022342981476149  T  T  T
 0.8746419482185355 0.0838883965746854 0.9034946173977647  T  T  T
 0.7504632630825629 0.3338532944588491 0.9030656200454894  T  T  T
 0.0011737902369098 0.3317672071029328 0.9047079801579357  T  T  T
 0.3748553523648613 0.5834892887994805 0.9030933420913591  T  T  T
 0.6254557408840855 0.5828076623433560 0.9033439249174869  T  T  T
 0.5006363097776561 0.8323312815270710 0.9027020992430030  T  T  T
 0.7496908247966675 0.8339478878005838 0.9036740829295894  T  T  T
 0.8744237524754389 0.5829595332766193 0.9033681916127060  T  T  T
 0.1241611175134088 0.5839183576903375 0.9030813274053205  T  T  T
-0.0011467793861428 0.8332805178444407 0.9032925628757525  T  T  T
 0.2494939263927563 0.8353700063199128 0.9044399053657526  T  T  T
 0.0000000000000000 0.1666666853711689 0.8090702727272756  F  F  F
 0.2499999757021314 0.1666666853711689 0.8090702727272756  F  F  F
 0.1249999878510692 0.4166666573144155 0.8090702727272756  F  F  F
 0.3750000607446680 0.4166666573144155 0.8090702727272756  F  F  F
 0.5000000485957372 0.1666666853711689 0.8090702727272756  F  F  F
 0.7500000242978686 0.1666666853711689 0.8090702727272756  F  F  F
 0.6250000364467994 0.4166666573144155 0.8090702727272756  F  F  F
 0.8750000121489308 0.4166666573144155 0.8090702727272756  F  F  F
 0.2499999757021314 0.6666666292576551 0.8090702727272756  F  F  F
 0.5000000485957372 0.6666666292576551 0.8090702727272756  F  F  F
 0.3750000607446680 0.9166666012008946 0.8090702727272756  F  F  F
 0.6250000364467994 0.9166666012008946 0.8090702727272756  F  F  F
 0.7500000242978686 0.6666666292576551 0.8090702727272756  F  F  F
 0.0000000000000000 0.6666666292576551 0.8090702727272756  F  F  F
 0.8750000121489308 0.9166666012008946 0.8090702727272756  F  F  F
 0.1250000850425366 0.9166666012008946 0.8090702727272756  F  F  F
 0.2508590309943850 0.1694088105819589 0.0619014396491562  T  T  T
 0.2667980160620916 0.1701415625014537 0.1166511113237337  T  T  T
 0.4977427943851403 0.1635260522145348 0.1126797027236801  T  T  T
```

```
Cu_COOH_Na
  1.0000000000000000
   10.2889689999999998    0.0000000000000000    0.0000000000000000
    0.0000000000000000    8.9105089999999993    0.0000000000000000
    0.0000000000000000    0.0000000000000000   22.0000000000000000
  Cu   C   O   H   Na
  48    1    2    1    1
Selective dynamics
Direct
 0.0019664251792784 0.0000359043345504 0.9974162724176703  T  T  T
 0.1262939531917938 0.2511441386249573 0.9980615406137120  T  T  T
 0.8784031163273232 0.2507780178018967 0.9969766551441779  T  T  T
 0.6253384180545934 0.7498172894685097 0.9970332595059729  T  T  T
 0.2501008829725002 -0.0001786480258870 0.9977383059788973  T  T  T
 0.3723005622602525 0.2505543636214373 0.9957662258031429  T  T  T
 0.4984426041736634 -0.0014230696690444 0.9969700953799095  T  T  T
 0.7524461183428042 -0.0030544353575688 0.9971229393637039  T  T  T
 0.6250014082393192 0.2501286744653726 0.0058999303915746  T  T  T
 0.4995505301731965 0.5042054354310799 0.9931309013041528  T  T  T
 0.2503404841310908 0.5005493874145405 0.9964659162172212  T  T  T
 0.3745686368099016 0.7487780098701668 0.9980191637962246  T  T  T
 0.7512766292276275 0.5019715162800163 0.9967188067740267  T  T  T
 0.0014757376068057 0.5007816495428854 0.9976196695504478  T  T  T
 0.8768430357559600 0.7496847448080197 0.9974115337510410  T  T  T
 0.1259044702156405 0.7497743028570574 0.9978829458571281  T  T  T
 0.1254675736714649 0.0840230171867073 0.9033670285895989  T  T  T
 0.3753208909116462 0.0830043388634493 0.9029353888664692  T  T  T
 0.2498937925154347 0.3340071975757886 0.9023002762080391  T  T  T
 0.5025947571557500 0.3308602111699059 0.9036807818125605  T  T  T
 0.6256253405310751 0.0850026053859316 0.9047203084827921  T  T  T
 0.8752074181705358 0.0837962722337431 0.9034183299060408  T  T  T
 0.7486649136248685 0.3317491511566489 0.9046246480217126  T  T  T
 0.0003594131767527 0.3337969741425243 0.9032642687194250  T  T  T
 0.3743286462605347 0.5842541670700238 0.9015199296049671  T  T  T
 0.6266115536875781 0.5833681294598334 0.9022339222160882  T  T  T
 0.5003082499273256 0.8329929475401393 0.9032910290709371  T  T  T
 0.7504141013575613 0.8336871403847567 0.9033707408425249  T  T  T
 0.8757185113995010 0.5831601615564114 0.9031506049213657  T  T  T
 0.1248342673205800 0.5842380142414103 0.9027004104329983  T  T  T
 0.0004955985632702 0.8337772471934640 0.9032563227817573  T  T  T
 0.2500725219147547 0.8338513307907036 0.9033538181985683  T  T  T
 0.0000000000000000 0.1666666853711689 0.8090702727272756  F  F  F
 0.2499999757021314 0.1666666853711689 0.8090702727272756  F  F  F
 0.1249999878510692 0.4166666573144155 0.8090702727272756  F  F  F
 0.3750000607446680 0.4166666573144155 0.8090702727272756  F  F  F
 0.5000000485957372 0.1666666853711689 0.8090702727272756  F  F  F
```

```
 0.7500000242978686 0.1666666853711689 0.8090702727272756 F F F
 0.6250000364467994 0.4166666573144155 0.8090702727272756 F F F
 0.8750000121489308 0.4166666573144155 0.8090702727272756 F F F
 0.2499999757021314 0.6666666292576551 0.8090702727272756 F F F
 0.5000000485957372 0.6666666292576551 0.8090702727272756 F F F
 0.3750000607446680 0.9166666012008946 0.8090702727272756 F F F
 0.6250000364467994 0.9166666012008946 0.8090702727272756 F F F
 0.7500000242978686 0.6666666292576551 0.8090702727272756 F F F
 0.0000000000000000 0.6666666292576551 0.8090702727272756 F F F
 0.8750000121489308 0.9166666012008946 0.8090702727272756 F F F
 0.1250000850425366 0.9166666012008946 0.8090702727272756 F F F
 0.6316532724212953 0.2377164991077186 0.0956512257027878 T T T
 0.5625526259154315 0.3049844272000855 0.1333482142136721 T T T
 0.7239449338714705 0.1421107437104611 0.1184997097096485 T T T
 0.7164477351693086 0.1455106984198331 0.1632908494106939 T T T
 0.3961650051084132 0.4525050515355192 0.1112770014344145 T T T
```

```
Cu_COH_Na
  1.0000000000000000
   10.2889689999999998    0.0000000000000000    0.0000000000000000
    0.0000000000000000    8.9105089999999993    0.0000000000000000
    0.0000000000000000    0.0000000000000000   22.0000000000000000
  Cu   C    O    H    Na
   48     1     1     1     1
Selective dynamics
Direct
-0.0003453411812421 0.0006386326383514 -0.0008380394387495 T T T
 0.1247743601277296 0.2506315683912476 -0.0015891275967486 T T T
 0.8778563090892431 0.2484216329735255 -0.0047665327661848 T T T
 0.6254158166594604 0.7518798500376545 -0.0030886278032338 T T T
 0.2487435072235034 0.0008542299660441 -0.0018626203597381 T T T
 0.3736593095123756 0.2497966614608212 -0.0023262455454709 T T T
 0.4997438109542388 -0.0019634364495463 -0.0027109945780456 T T T
 0.7518116741427165 -0.0030366091636257 -0.0041428386110613 T T T
 0.6255150595161073 0.2405901193389310 -0.0009085071602319 T T T
 0.4952626747712717 0.5062193639731052 0.0022472384752390 T T T
 0.2482375228204097 0.5011256181539321 -0.0018988268404406 T T T
 0.3725813578268328 0.7522861813410637 -0.0024976414224964 T T T
 0.7516150643837836 0.5009545226918539 0.0021720563386235 T T T
 0.0008288880354633 0.5001447238905888 -0.0019688949788422 T T T
 0.8759446634750515 0.7499056207647955 -0.0021146132832944 T T T
 0.1242927123225671 0.7509982795591164 -0.0013377211999630 T T T
 0.1245669988779178 0.0843697038751359 0.9039511437929111 T T T
 0.3747676527580237 0.0834005290575712 0.9036100825031625 T T T
 0.2501213551137034 0.3339198167148868 0.9036010980341153 T T T
 0.5002609222667945 0.3337171719739339 0.9034832938245977 T T T
 0.6249583719196476 0.0846522281957011 0.9032999085745662 T T T
 0.8756098502171820 0.0829342411048841 0.9022522629366110 T T T
 0.7493525066528656 0.3336097529145180 0.9028702285418028 T T T
 0.0009973041385758 0.3347981075338958 0.9027755042420182 T T T
 0.3763942109775176 0.5823161130484431 0.9046018706014749 T T T
 0.6246677035339521 0.5819242520277297 0.9035987910374188 T T T
 0.4997157932312187 0.8322144860576185 0.9032249366476158 T T T
 0.7503749456224318 0.8311233715482695 0.9027094410224360 T T T
 0.8736979576310209 0.5815590192363427 0.9047762369140055 T T T
 0.1251786209159876 0.5853950742197759 0.9033412564269722 T T T
 0.0003379482870885 0.8338906613630025 0.9036623256024537 T T T
 0.2492947049890776 0.8340315389456824 0.9032783343776937 T T T
 0.0000000000000000 0.1666666853711689 0.8090702727272756 F F F
 0.2499999757021314 0.1666666853711689 0.8090702727272756 F F F
 0.1249999878510692 0.4166666573144155 0.8090702727272756 F F F
 0.3750000607446680 0.4166666573144155 0.8090702727272756 F F F
 0.5000000485957372 0.1666666853711689 0.8090702727272756 F F F
 0.7500000242978686 0.1666666853711689 0.8090702727272756 F F F
 0.6250000364467994 0.4166666573144155 0.8090702727272756 F F F
 0.8750000121489308 0.4166666573144155 0.8090702727272756 F F F
 0.2499999757021314 0.6666666292576551 0.8090702727272756 F F F
 0.5000000485957372 0.6666666292576551 0.8090702727272756 F F F
 0.3750000607446680 0.9166666012008946 0.8090702727272756 F F F
 0.6250000364467994 0.9166666012008946 0.8090702727272756 F F F
 0.7500000242978686 0.6666666292576551 0.8090702727272756 F F F
 0.0000000000000000 0.6666666292576551 0.8090702727272756 F F F
 0.8750000121489308 0.9166666012008946 0.8090702727272756 F F F
 0.1250000850425366 0.9166666012008946 0.8090702727272756 F F F
 0.6184895044010787 0.4055150836406446 0.0544223679889144 T T T
 0.6265596408503693 0.3999900797908996 0.1178866574186519 T T T
 0.6023618007637314 0.4977445267016433 0.1346364955271498 T T T
 0.7461212086602084 0.1757799593395271 0.1108825460449020 T T T
```

```
Cu_CHO_Na
  1.0000000000000000
   10.2889689999999998    0.0000000000000000    0.0000000000000000
    0.0000000000000000    8.9105089999999993    0.0000000000000000
    0.0000000000000000    0.0000000000000000   22.0000000000000000
  Cu   C    O    H    Na
   48     1     1     1     1
Selective dynamics
Direct
 0.0006524172008218 -0.0004698892584565 -0.0022872055282648 T T T
 0.1258855325561041 0.2502422734459100 -0.0020658418322762 T T T
 0.8764586957456495 0.2478580933628097 -0.0028529573364647 T T T
 0.6235302647822077 0.7512811951961138 -0.0032164658658251 T T T
 0.2501178440802390 -0.0003065977593648 -0.0021109614113073 T T T
 0.3747343088706789 0.2500868776055994 -0.0021194120735961 T T T
 0.4991916373536499 -0.0006512862615740 -0.0023556883234404 T T T
 0.7500250672241777 -0.0004276188484151 -0.0026981573631987 T T T
 0.6239360290672522 0.2487394657369011 -0.0025641196284320 T T T
 0.4968018399213991 0.4985049295811090 -0.0074157214621041 T T T
 0.2511731852016035 0.5009001370018683 -0.0014169367179833 T T T
 0.3746587877249885 0.7495275967278212 -0.0030276023028975 T T T
 0.7485147011404959 0.4994877836463109 0.0069588263937045 T T T
 0.0034937432650884 0.4999669666049643 -0.0031696788459896 T T T
 0.8753383333211732 0.7500788197113410 -0.0022670696240876 T T T
 0.1258176337149638 0.7499718399373709 -0.0018204900509274 T T T
 0.1249060034847820 0.0837619457896565 0.9034892467796054 T T T
 0.3747088901001528 0.0833576225181334 0.9034320776216791 T T T
 0.2500361581528514 0.3338617409981104 0.9033926456813403 T T T
 0.5004230669457551 0.3315032497621845 0.9021294751325863 T T T
 0.6252339436705006 0.0828690558470677 0.9033971092970162 T T T
 0.8749635868087381 0.0832131751280575 0.9032880410892742 T T T
 0.7501137701165835 0.3354988429949068 0.9047581890053512 T T T
-0.0002519646423950 0.3334023664512837 0.9033441078632070 T T T
 0.3739412626206241 0.5842571322163503 0.9015714855701059 T T T
 0.6275383477800806 0.5824279644946688 0.9033046956284070 T T T
 0.4999582534382483 0.8330626540700022 0.9028817496626359 T T T
 0.7504428184336572 0.8326929416610095 0.9032999391323608 T T T
 0.8734926673544717 0.5816404291450121 0.9047280404386465 T T T
 0.1253218698166885 0.5838991240931253 0.9031274821160273 T T T
-0.0000886676605171 0.8328020661183165 0.9036140350859732 T T T
 0.2496085106734476 0.8340123207177402 0.9029667635424788 T T T
 0.0000000000000000 0.1666666853711689 0.8090702727272756 F F F
 0.2499999757021314 0.1666666853711689 0.8090702727272756 F F F
 0.1249999878510692 0.4166666573144155 0.8090702727272756 F F F
 0.3750000607446680 0.4166666573144155 0.8090702727272756 F F F
 0.5000000485957372 0.1666666853711689 0.8090702727272756 F F F
 0.7500000242978686 0.1666666853711689 0.8090702727272756 F F F
 0.6250000364467994 0.4166666573144155 0.8090702727272756 F F F
 0.8750000121489308 0.4166666573144155 0.8090702727272756 F F F
 0.2499999757021314 0.6666666292576551 0.8090702727272756 F F F
 0.5000000485957372 0.6666666292576551 0.8090702727272756 F F F
 0.3750000607446680 0.9166666012008946 0.8090702727272756 F F F
 0.6250000364467994 0.9166666012008946 0.8090702727272756 F F F
 0.7500000242978686 0.6666666292576551 0.8090702727272756 F F F
 0.0000000000000000 0.6666666292576551 0.8090702727272756 F F F
 0.8750000121489308 0.9166666012008946 0.8090702727272756 F F F
 0.1250000850425366 0.9166666012008946 0.8090702727272756 F F F
 0.7438459497809921 0.4945018772476101 0.0960389997469290 T T T
 0.6522999449606850 0.5212245915400057 0.1309999720287230 T T T
 0.8379526439691259 0.4626916245238495 0.1183419105840742 T T T
 0.4451844174534694 0.5837569652903070 0.1121606735448590 T T T
```

```
Cu111_C_K
  1.00000000000000
   10.2889409999999994    0.0000000000000000    0.0000000000000000
    0.0000000000000000    8.9104840000000003    0.0000000000000000
    0.0000000000000000    0.0000000000000000   22.0000000000000000
  Cu   C    K
   48     1     1
Selective dynamics
Direct
-0.0000000000000000 -0.0015966831374565 -0.0026645822204923 T T T
 0.1230882587718981 0.2500915505736603 -0.0024878379326440 T T T
 0.8769117412281017 0.2500915505736603 -0.0024878379326440 T T T
 0.6256780784670276 0.7449816932644564 -0.0032945294296177 T T T
 0.2473365876588606 -0.0038350115668183 -0.0022462246298007 T T T
 0.3688462572043528 0.2504774863825571 0.0009542579051831 T T T
 0.5000000485958651 -0.0101053831533297 0.0018100608422461 T T T
 0.7526634123411395 -0.0038350115668183 -0.0022462246298007 T T T
 0.6311537427956474 0.2504774863825571 0.0009542579051831 T T T
 0.5000000485958651 0.5006572661897576 -0.0059300033213746 T T T
 0.2490279002026975 0.4992667559154050 -0.0029751215011052 T T T
 0.3743219215329722 0.7449816932644564 -0.0032945294296177 T T T
 0.7509720997973025 0.4992667559154050 -0.0029751215011052 T T T
-0.0000000000000000 0.4993529781793070 -0.0025525692149166 T T T
 0.8757418299685237 0.7476868273380732 -0.0028075273296991 T T T
 0.1242581700314766 0.7476868273380732 -0.0028075273296991 T T T
 0.1252702028817770 0.0834778224556949 0.9033462635822164 T T T
 0.3753932098772369 0.0819404907687760 0.9044423870457914 T T T
 0.2506498665161269 0.3317065113117716 0.9035555310390904 T T T
 0.5000000485958651 0.3301731774084404 0.9033949076010052 T T T
 0.6246067901227630 0.0819404907687760 0.9044423870457914 T T T
 0.8747297971182231 0.0834778224556949 0.9033462635822164 T T T
 0.7493501334838735 0.3317065113117716 0.9035555310390904 T T T
-0.0000000000000000 0.3328907697366311 0.9028616786165130 T T T
 0.3742897903943229 0.5813623069277761 0.9021082237661412 T T T
 0.6257102096056774 0.5813623069277761 0.9021082237661412 T T T
 0.5000000485958651 0.8329161552394244 0.9037805577520462 T T T
 0.7499755198690006 0.8330084195507170 0.9032683477249461 T T T
 0.8754454158439187 0.5824005921228634 0.9027145239862645 T T T
 0.1245545841560812 0.5824005921228634 0.9027145239862645 T T T
-0.0000000000000000 0.8329170920094997 0.9028417149975133 T T T
 0.2500244801309995 0.8330084195507170 0.9032683477249461 T T T
 0.0000000000000000 0.1666667040757801 0.8090707727272743 F F F
 0.2499999757020674 0.1666667040757801 0.8090707727272743 F F F
 0.1250000364468988 0.4166667040757801 0.8090707727272743 F F F
 0.3750000121489663 0.4166667040757801 0.8090707727272743 F F F
 0.5000000485958651 0.1666667040757801 0.8090707727272743 F F F
 0.7500000242979326 0.1666667040757801 0.8090707727272743 F F F
 0.6249999878510337 0.4166667040757801 0.8090707727272743 F F F
 0.8749999635531012 0.4166667040757801 0.8090707727272743 F F F
 0.2499999757020674 0.6666667040757801 0.8090707727272743 F F F
 0.5000000485958651 0.6666667040757801 0.8090707727272743 F F F
 0.3750000121489663 0.9166667040757801 0.8090707727272743 F F F
 0.6249999878510337 0.9166667040757801 0.8090707727272743 F F F
 0.7500000242979326 0.6666667040757801 0.8090707727272743 F F F
 0.0000000000000000 0.6666667040757801 0.8090707727272743 F F F
 0.8749999635531012 0.9166667040757801 0.8090707727272743 F F F
 0.1250000364468988 0.9166667040757801 0.8090707727272743 F F F
 0.4999999514041349 0.1643422700746855 0.0493585476283194 T T T
 0.5000000485958651 0.4074157779790982 0.1301217189569693 T T T
```

```
Cu111_CH_K
  1.00000000000000
   10.2889409999999994    0.0000000000000000    0.0000000000000000
    0.0000000000000000    8.9104840000000003    0.0000000000000000
    0.0000000000000000    0.0000000000000000   22.0000000000000000
  Cu   C    H    K
   48     1     1     1
Selective dynamics
Direct
 0.0008727719343549 0.0016019834819299 -0.0018164702364854 T T T
 0.1225956013495695 0.2514943015097056 -0.0045880286089270 T T T
 0.8771192477973523 0.2513236740708461 -0.0021489209837684 T T T
 0.6255183764879128 0.7475425092794509 -0.0035133045317986 T T T
 0.2476529839099089 -0.0024974211758362 -0.0047282156920899 T T T
 0.3699011603270148 0.2541235878411249 0.0010667591924323 T T T
 0.4993048466325172 -0.0070406911244976 0.0024976778678820 T T T
 0.7520334282521762 -0.0011230310296837 -0.0023674006366726 T T T
 0.6300469774835344 0.2521378186727258 0.0021064814827625 T T T
 0.5002941548124142 0.5001602985665754 -0.0031580435265945 T T T
 0.2479561337450389 0.5006300041420706 -0.0030189856115667 T T T
 0.3740378751617265 0.7473703517215174 -0.0036680994096253 T T T
 0.7518577546519091 0.5011919196218662 -0.0029400915349404 T T T
 0.0000457860144272 0.5010577470798538 -0.0019623360006088 T T T
 0.8759966780695009 0.7512517170575769 -0.0022113503238845 T T T
 0.1243701730291659 0.7508073642449908 -0.0020156812358216 T T T
 0.1249260807007323 0.0840352218370687 0.9022067520218665 T T T
 0.3757900243579774 0.0829592616173799 0.9027736439584780 T T T
 0.2521273045867676 0.3318389694327892 0.9034067698469367 T T T
 0.5004086261188905 0.3315603999790525 0.9031389329350764 T T T
 0.6244573847314869 0.0829429654346705 0.9038172540432496 T T T
 0.8746647246865464 0.0844382624012861 0.9034559447056907 T T T
 0.7486471693883583 0.3322131195961091 0.9043160228454312 T T T
-0.0007804351695801 0.3355860191284309 0.9024241741077167 T T T
 0.3751807593092781 0.5812944169209202 0.9029892606312089 T T T
 0.6251085659235434 0.5819762910820749 0.9030019433385520 T T T
 0.5000362936395422 0.8355904563804659 0.9041624135159906 T T T
 0.7500160747786133 0.8341213188035397 0.9033013215537589 T T T
 0.8753142443962681 0.5848020632102491 0.9031735303720798 T T T
 0.1244380004935321 0.5840895523770430 0.9031331099087472 T T T
 0.0000235111836212 0.8348065955306300 0.9034450388307756 T T T
 0.2500768848433687 0.8325211132387602 0.9026985171571029 T T T
 0.0000000000000000 0.1666667040757801 0.8090707727272743 F F F
 0.2499999757020674 0.1666667040757801 0.8090707727272743 F F F
 0.1250000364468988 0.4166667040757801 0.8090707727272743 F F F
 0.3750000121489663 0.4166667040757801 0.8090707727272743 F F F
 0.5000000485958651 0.1666667040757801 0.8090707727272743 F F F
 0.7500000242979326 0.1666667040757801 0.8090707727272743 F F F
 0.6249999878510337 0.4166667040757801 0.8090707727272743 F F F
 0.8749999635531012 0.4166667040757801 0.8090707727272743 F F F
 0.2499999757020674 0.6666667040757801 0.8090707727272743 F F F
 0.5000000485958651 0.6666667040757801 0.8090707727272743 F F F
 0.3750000121489663 0.9166667040757801 0.8090707727272743 F F F
 0.6249999878510337 0.9166667040757801 0.8090707727272743 F F F
 0.7500000242979326 0.6666667040757801 0.8090707727272743 F F F
 0.0000000000000000 0.6666667040757801 0.8090707727272743 F F F
 0.8749999635531012 0.9166667040757801 0.8090707727272743 F F F
 0.1250000364468988 0.9166667040757801 0.8090707727272743 F F F
 0.4975962057007321 0.1674066018254166 0.0540431062138579 T T T
 0.5140050659839821 0.1694701731930127 0.1037264699424970 T T T
 0.2382061202875548 0.1645790292204632 0.1265026423574871 T T T
```

```
Cu111_CH2_K
  1.00000000000000
   10.2889409999999994    0.0000000000000000    0.0000000000000000
    0.0000000000000000    8.9104840000000003    0.0000000000000000
    0.0000000000000000    0.0000000000000000   22.0000000000000000
  Cu   C    H    K
   48     1     2     1
Selective dynamics
Direct
 0.0002224873873113 0.0003163935992684 -0.0020478784021338 T T T
 0.1249687385456172 0.2508705047278469 -0.0025104472745621 T T T
 0.8767632274081459 0.2510060167594030 -0.0025413428605077 T T T
 0.6259621525047208 0.7493987743785214 -0.0028462723188008 T T T
 0.2472522668842999 -0.0025985749587385 -0.0031302720912737 T T T
 0.3746849874075742 0.2480872529690249 0.0000737081658896 T T T
 0.4965114934709680 -0.0028439297545854 0.0027793693575215 T T T
 0.7541773812586458 -0.0023420537489156 -0.0038593951580600 T T T
 0.6276285617423781 0.2566481340107820 0.0022161124644063 T T T
 0.4997366846314099 0.5048804811714683 -0.0066030193117151 T T T
 0.2505044389822698 0.4995634008548485 -0.0027773206765491 T T T
 0.3745858109869050 0.7484122213400121 -0.0025064457480101 T T T
 0.7526197623915298 0.5027049268483667 -0.0053164089681673 T T T
 0.0006723673890685 0.5004885596475737 -0.0022452246082774 T T T
 0.8759557735475244 0.7494084341419870 -0.0017886776604416 T T T
 0.1247282587018125 0.7487869326649078 -0.0024187872714259 T T T
 0.1247858581447915 0.0838991387752476 0.9033604978711437 T T T
 0.3749582389727943 0.0828873020742814 0.9035182198180536 T T T
 0.2525789216599477 0.3315410131675380 0.9046187141515447 T T T
 0.5002640930967510 0.3320274859405081 0.9028005343871357 T T T
 0.6246180804262749 0.0834485393341722 0.9046078908828190 T T T
 0.8754153065662784 0.0844079824438382 0.9031516117301291 T T T
 0.7480661005430126 0.3309290891946123 0.9039617449053854 T T T
 0.0006175345438146 0.3346488046428044 0.9029483111852882 T T T
 0.3739625852213739 0.5836205674437166 0.9020028203840043 T T T
 0.6257311822022255 0.5833673938248297 0.9014786774295527 T T T
 0.5000168449207490 0.8365326227903769 0.9047858282367695 T T T
 0.7498108824909557 0.8333941250478780 0.9030453058816680 T T T
 0.8765782137182004 0.5845649589085790 0.9023226354897687 T T T
 0.1252644799054097 0.5833607618695631 0.9029469745672479 T T T
 0.0000499138748201 0.8342543778457151 0.9034574828915952 T T T
 0.2501618797554047 0.8332172957200030 0.9032319788771322 T T T
 0.0000000000000000 0.1666667040757801 0.8090707727272743 F F F
 0.2499999757020674 0.1666667040757801 0.8090707727272743 F F F
 0.1250000364468988 0.4166667040757801 0.8090707727272743 F F F
 0.3750000121489663 0.4166667040757801 0.8090707727272743 F F F
 0.5000000485958651 0.1666667040757801 0.8090707727272743 F F F
 0.7500000242979326 0.1666667040757801 0.8090707727272743 F F F
 0.6249999878510337 0.4166667040757801 0.8090707727272743 F F F
 0.8749999635531012 0.4166667040757801 0.8090707727272743 F F F
 0.2499999757020674 0.6666667040757801 0.8090707727272743 F F F
 0.5000000485958651 0.6666667040757801 0.8090707727272743 F F F
 0.3750000121489663 0.9166667040757801 0.8090707727272743 F F F
 0.6249999878510337 0.9166667040757801 0.8090707727272743 F F F
 0.7500000242979326 0.6666667040757801 0.8090707727272743 F F F
 0.0000000000000000 0.6666667040757801 0.8090707727272743 F F F
 0.8749999635531012 0.9166667040757801 0.8090707727272743 F F F
 0.1250000364468988 0.9166667040757801 0.8090707727272743 F F F
 0.5127114807557542 0.1665222296720932 0.0652839778041576 T T T
 0.5716399206078046 0.1023197483271687 0.0983478866585606 T T T
 0.4301113569045938 0.2152215105837076 0.0916109196975183 T T T
 0.6194966900019986 0.4719411047650316 0.1248297992935727 T T T
```

```
Cu111_CH3_K
  1.00000000000000
   10.2889409999999994    0.0000000000000000    0.0000000000000000
    0.0000000000000000    8.9104840000000003    0.0000000000000000
    0.0000000000000000    0.0000000000000000   22.0000000000000000
  Cu   C    H    K
   48     1     3     1
Selective dynamics
Direct
-0.0002541122422658 -0.0022877311299979 -0.0018409824651647 T T T
 0.1240286897266875 0.2477345183617387 -0.0024552239895380 T T T
 0.8752609208098538 0.2480570220449411 -0.0019732329280746 T T T
 0.6260747479402016 0.7507944868920045 -0.0055214201434532 T T T
 0.2461753272206140 -0.0045548860475371 -0.0040609634679357 T T T
 0.3766820905856131 0.2469365188738322 0.0011571483953572 T T T
 0.4997690888437510 0.0005105913111601 0.0013229532710980 T T T
 0.7534187269511239 -0.0037581956955297 -0.0030981443905716 T T T
 0.6244742389697600 0.2487949206377151 0.0010624530153923 T T T
 0.5011078460215568 0.5036047792559870 -0.0046067123681609 T T T
 0.2509345960834812 0.4982293803240659 -0.0024717894016022 T T T
 0.3747777543919130 0.7480370993736114 -0.0024340057611248 T T T
 0.7502496366924755 0.4984624019712309 -0.0041843575079958 T T T
-0.0000006777425805 0.4974620366357171 -0.0019941945297135 T T T
 0.8746917607766057 0.7462739960115136 -0.0019396464915013 T T T
 0.1247944271724147 0.7462246841521614 -0.0022214026874830 T T T
 0.1245452587603858 0.0825426995073285 0.9030746723826623 T T T
 0.3747897122703161 0.0824575079453334 0.9037194216332207 T T T
 0.2527065511337340 0.3301162917388679 0.9049353644509515 T T T
 0.5001735911644465 0.3313746040979315 0.9036779224021471 T T T
 0.6247394263547028 0.0821558049879509 0.9041536511951068 T T T
 0.8758785300357451 0.0825304969908577 0.9033844006630161 T T T
 0.7480506423164152 0.3296058088153063 0.9042121880735053 T T T
 0.0000643565059620 0.3326635079304813 0.9031458588823195 T T T
 0.3748192489335574 0.5825862809962569 0.9026125811935392 T T T
 0.6256556963282450 0.5827015021212266 0.9015669560384186 T T T
 0.4988886894333719 0.8359315842168406 0.9041684895120521 T T T
 0.7511105751276285 0.8325072550124113 0.9022797451006543 T T T
 0.8758202234644029 0.5821471758890409 0.9026197461772579 T T T
 0.1253333272387310 0.5814834159403079 0.9031884594290857 T T T
 0.0003181339426979 0.8323206574214739 0.9034561678615856 T T T
 0.2499549624526802 0.8310662441772541 0.9030745542024357 T T T
 0.0000000000000000 0.1666667040757801 0.8090707727272743 F F F
 0.2499999757020674 0.1666667040757801 0.8090707727272743 F F F
 0.1250000364468988 0.4166667040757801 0.8090707727272743 F F F
 0.3750000121489663 0.4166667040757801 0.8090707727272743 F F F
 0.5000000485958651 0.1666667040757801 0.8090707727272743 F F F
 0.7500000242979326 0.1666667040757801 0.8090707727272743 F F F
 0.6249999878510337 0.4166667040757801 0.8090707727272743 F F F
 0.8749999635531012 0.4166667040757801 0.8090707727272743 F F F
 0.2499999757020674 0.6666667040757801 0.8090707727272743 F F F
 0.5000000485958651 0.6666667040757801 0.8090707727272743 F F F
 0.3750000121489663 0.9166667040757801 0.8090707727272743 F F F
 0.6249999878510337 0.9166667040757801 0.8090707727272743 F F F
 0.7500000242979326 0.6666667040757801 0.8090707727272743 F F F
 0.0000000000000000 0.6666667040757801 0.8090707727272743 F F F
 0.8749999635531012 0.9166667040757801 0.8090707727272743 F F F
 0.1250000364468988 0.9166667040757801 0.8090707727272743 F F F
 0.4890127650532369 0.1584161135634119 0.0798721392468891 T T T
 0.4034189361639263 0.2180094164178987 0.0986398680555937 T T T
 0.5767779528926331 0.2126519461405524 0.0989053651551481 T T T
 0.4857783873682347 0.0434929097531642 0.0990112208127121 T T T
 0.6574268134445839 0.6263041396910158 0.1248341221860604 T T T
```

```
Cu_CO_K
  1.00000000000000
   10.2889689999999998    0.0000000000000000    0.0000000000000000
    0.0000000000000000    8.9105089999999993    0.0000000000000000
    0.0000000000000000    0.0000000000000000   22.0000000000000000
  Cu   C    O    K
   48     1     1     1
Selective dynamics
Direct
-0.0051977566228777 0.0002701468161984 -0.0026490606321760 T T T
 0.1204656267060968 0.2518164309496060 0.0013996758969437 T T T
 0.8715105797268738 0.2519642586888863 -0.0017088343425664 T T T
 0.6221471802926478 0.7516063162475758 -0.0014094984271224 T T T
 0.2482508712151396 -0.0011169888389788 0.0004092870516225 T T T
 0.3744953323060026 0.2505899417020944 -0.0000635899670525 T T T
 0.5006767892913079 -0.0003627981960837 -0.0076068681849964 T T T
 0.7465801225791269 0.0016636585495223 -0.0016935693039824 T T T
 0.6232732516600996 0.2508163391495842 -0.0019141750126003 T T T
 0.4975835842052679 0.5019659362532005 -0.0021559105700569 T T T
 0.2471940566997432 0.5035598928243126 -0.0034488411785217 T T T
 0.3739237289624857 0.7515091447834255 -0.0025628039158526 T T T
 0.7466007498436568 0.5015938062970315 -0.0011615315845722 T T T
-0.0036321376116878 0.5019488302992811 -0.0022788007252081 T T T
 0.8710791019333816 0.7512922911997700 -0.0016799981765882 T T T
 0.1215414201718953 0.7512379724716256 -0.0024340024143545 T T T
 0.1232164224890430 0.0841743364744481 0.9032325751013827 T T T
 0.3720015821034390 0.0845622731753837 0.9020374334683837 T T T
 0.2477642668109122 0.3336329119312914 0.9033365020484995 T T T
 0.4974728700783300 0.3334455923465567 0.9041295005067733 T T T
 0.6250059133163457 0.0860461982040140 0.9020000040727760 T T T
 0.8734352404788910 0.0854330752543013 0.9034898445896276 T T T
 0.7486277403871303 0.3356472602527763 0.9035060751962449 T T T
```

```
-0.0005359745161530 0.3334923213009251 0.9046656174912463  T  T  T
 0.3730533976159480 0.5842660515033115 0.9031538018606883  T  T  T
 0.6234956402706427 0.5839060368333832 0.9035818015982416  T  T  T
 0.4990249171455793 0.8321885236559086 0.9020078181096742  T  T  T
 0.7478846290473249 0.8352464415286512 0.9038906173023037  T  T  T
 0.8723267299515362 0.5847291084313418 0.9035885301282951  T  T  T
 0.1222479540824160 0.5848506911100548 0.9032482225591255  T  T  T
-0.0030911816137898 0.8340531173150239 0.9034972453978165  T  T  T
 0.2479035537759592 0.8356986179963187 0.9042600760884314  T  T  T
 0.0000000000000000 0.1666666853711689 0.8090702727272756  F  F  F
 0.2499999757021314 0.1666666853711689 0.8090702727272756  F  F  F
 0.1249999878510692 0.4166666573144155 0.8090702727272756  F  F  F
 0.3750000607446680 0.4166666573144155 0.8090702727272756  F  F  F
 0.5000000485957372 0.1666666853711689 0.8090702727272756  F  F  F
 0.7500000242978686 0.1666666853711689 0.8090702727272756  F  F  F
 0.6250000364467994 0.4166666573144155 0.8090702727272756  F  F  F
 0.8750000121489308 0.4166666573144155 0.8090702727272756  F  F  F
 0.2499999757021314 0.6666666292576551 0.8090702727272756  F  F  F
 0.5000000485957372 0.6666666292576551 0.8090702727272756  F  F  F
 0.3750000607446680 0.9166666012008946 0.8090702727272756  F  F  F
 0.6250000364467994 0.9166666012008946 0.8090702727272756  F  F  F
 0.7500000242978686 0.6666666292576551 0.8090702727272756  F  F  F
 0.0000000000000000 0.6666666292576551 0.8090702727272756  F  F  F
 0.8750000121489308 0.9166666012008946 0.8090702727272756  F  F  F
 0.1250000850425366 0.9166666012008946 0.8090702727272756  F  F  F
 0.2458225511299182 0.1681606020191133 0.0619896781433694  T  T  T
 0.2547346563749784 0.1652227312809533 0.1170139826458728  T  T  T
 0.4841323425059502 0.0162525639880206 0.1287670457205284  T  T  T
```

```
Cu_COOH_K
  1.00000000000000
   10.2889689999999998    0.0000000000000000    0.0000000000000000
    0.0000000000000000    8.9105089999999993    0.0000000000000000
    0.0000000000000000    0.0000000000000000   22.0000000000000000
  Cu   C    O    H    K
   48    1    2    1    1
Selective dynamics
Direct
  0.0006716375147453  0.0016592434852220 -0.0039994582285268   T   T   T
  0.1239928131204256  0.2518066611444576 -0.0019956351953841   T   T   T
  0.8785127364301962  0.2513542808969675 -0.0047517058408579   T   T   T
  0.6256593248170054  0.7550513886837059 -0.0035905330491311   T   T   T
  0.2482715308461166  0.0020720017940039 -0.0024995424116967   T   T   T
  0.3708256573596928  0.2496051520386395 -0.0035906288857790   T   T   T
  0.4987579945116885  0.0019060942099592 -0.0029911039683163   T   T   T
  0.7520600007482654 -0.0009692806594767 -0.0057078996102743   T   T   T
  0.6287618293192111  0.2487469698962994  0.0050733338811951   T   T   T
  0.4952225550305104  0.5087833313677340  0.0011158906495293   T   T   T
  0.2466943062668095  0.5025818544868330 -0.0027517655813456   T   T   T
  0.3727864776530456  0.7551878622470016 -0.0029222444370609   T   T   T
  0.7535195536237832  0.5039648056868640 -0.0036839090481847   T   T   T
  0.0001009777633906  0.5024645570068070 -0.0023603783799019   T   T   T
  0.8756771896787984  0.7527949682772923 -0.0023932400426583   T   T   T
  0.1239193520498323  0.7528193904015870 -0.0022588101288666   T   T   T
  0.1253501884123319  0.0853745254414705  0.9026533340533180   T   T   T
  0.3750397088151547  0.0848024473102188  0.9029964785138765   T   T   T
  0.2500950815806890  0.3353186650306574  0.9032317756694622   T   T   T
  0.5010644396412719  0.3352139048247360  0.9057614406884558   T   T   T
  0.6242920565095104  0.0882123581914162  0.9045956662470755   T   T   T
  0.8753325392137590  0.0848936078074270  0.9016918268107375   T   T   T
  0.7479920907452696  0.3335773195158304  0.9039506012670318   T   T   T
  0.0007298552348066  0.3355630116913859  0.9025307411666522   T   T   T
  0.3760723857174578  0.5836388894500135  0.9045476261415588   T   T   T
  0.6234671400291185  0.5831523351234411  0.9044125468055537   T   T   T
  0.4998159909722482  0.8350630075172721  0.9029494912502126   T   T   T
  0.7497863139920433  0.8331565106135713  0.9019659631911141   T   T   T
  0.8748937112410148  0.5842383325575171  0.9029487206849458   T   T   T
  0.1250217327659013  0.5855955668853010  0.9028787317082722   T   T   T
  0.0001040251894907  0.8344386211507742  0.9025104950524673   T   T   T
  0.2495398488854133  0.8350236080392869  0.9029395652006738   T   T   T
  0.0000000000000000  0.1666666853711689  0.8090702727272756   F   F   F
  0.2499999757021314  0.1666666853711689  0.8090702727272756   F   F   F
  0.1249999878510692  0.4166666573144155  0.8090702727272756   F   F   F
  0.3750000607446680  0.4166666573144155  0.8090702727272756   F   F   F
  0.5000000485957372  0.1666666853711689  0.8090702727272756   F   F   F
  0.7500000242978686  0.1666666853711689  0.8090702727272756   F   F   F
  0.6250000364467994  0.4166666573144155  0.8090702727272756   F   F   F
  0.8750000121489308  0.4166666573144155  0.8090702727272756   F   F   F
  0.2499999757021314  0.6666666292576551  0.8090702727272756   F   F   F
  0.5000000485957372  0.6666666292576551  0.8090702727272756   F   F   F
  0.3750000607446680  0.9166666012008946  0.8090702727272756   F   F   F
  0.6250000364467994  0.9166666012008946  0.8090702727272756   F   F   F
  0.7500000242978686  0.6666666292576551  0.8090702727272756   F   F   F
  0.0000000000000000  0.6666666292576551  0.8090702727272756   F   F   F
  0.8750000121489308  0.9166666012008946  0.8090702727272756   F   F   F
  0.1250000850425366  0.9166666012008946  0.8090702727272756   F   F   F
  0.6034801225098311  0.3300543640527935  0.0872853208846949   T   T   T
  0.5349851587041854  0.4425569943617290  0.0979778203694021   T   T   T
  0.6604270309072086  0.2660393491693199  0.1392817342333518   T   T   T
  0.6295753599338988  0.3291700985499357  0.1733084636028147   T   T   T
  0.8599213202173603  0.0683382710499784  0.1270995436080548   T   T   T
```

```
Cu_COH_K
  1.0000000000000000
   10.2889689999999998    0.0000000000000000    0.0000000000000000
    0.0000000000000000    8.9105089999999993    0.0000000000000000
    0.0000000000000000    0.0000000000000000   22.0000000000000000
  Cu   C    O    H    K
   48    1    1    1    1
Selective dynamics
Direct
 -0.0003596298901458  0.0006204440338886 -0.0016156572183679   T   T   T
  0.1246105104890678  0.2507700836880486 -0.0018784808980217   T   T   T
  0.8779436826187423  0.2485909810065597 -0.0048189924127807   T   T   T
  0.6251974848463008  0.7523270680488974 -0.0032284283216843   T   T   T
  0.2488011133790776  0.0014080607848004 -0.0020678932443309   T   T   T
  0.3735502730756468  0.2499570440180308 -0.0023129988140463   T   T   T
  0.4994632445811326 -0.0017517810350313 -0.0031073022476760   T   T   T
  0.7517019289138923 -0.0037624366850402 -0.0052341334747164   T   T   T
  0.6259479717488601  0.2400082430975990  0.0006444941360879   T   T   T
  0.4955441506270075  0.5061729552323726  0.0016828762010159   T   T   T
  0.2480584986784248  0.5014035950477521 -0.0020890284984089   T   T   T
  0.3728138950611259  0.7527923397159632 -0.0027273812201814   T   T   T
  0.7510199452775167  0.5007605159210488  0.0022314973221977   T   T   T
  0.0002651501183149  0.5003351953028783 -0.0020604245579979   T   T   T
  0.8754297041697150  0.7500899066757077 -0.0022505144863388   T   T   T
  0.1239999724783295  0.7511469588275959 -0.0015226119199120   T   T   T
  0.1247264531367326  0.0844611716907810  0.9036064828026322   T   T   T
  0.3745970083155911  0.0837131702861855  0.9034099895766539   T   T   T
  0.2501162755734334  0.3342746475951073  0.9035561975625971   T   T   T
  0.5003344653667828  0.3336658820470981  0.9035040302281325   T   T   T
  0.6247397906281241  0.0854603888613205  0.9032789658866177   T   T   T
  0.8756644464645319  0.0833009904328613  0.9021500060931386   T   T   T
  0.7491737198232593  0.3334413412772194  0.9029169812574744   T   T   T
  0.0008075534580212  0.3347575418212765  0.9027640140274370   T   T   T
  0.3761749690652360  0.5826131223945977  0.9043228507484762   T   T   T
  0.6247421080301375  0.5818559656888704  0.9033203493581254   T   T   T
  0.4996856541509818  0.8324635573042414  0.9030460200282000   T   T   T
  0.7502466158030529  0.8311338500648129  0.9024235345473502   T   T   T
  0.8733561070568318  0.5814540213175942  0.9046530922962567   T   T   T
  0.1249357382476505  0.5853724719390719  0.9033234237077792   T   T   T
  0.0001565403223006  0.8336550959093493  0.9033954901919260   T   T   T
  0.2491527977510500  0.8343245640916617  0.9032895688087872   T   T   T
  0.0000000000000000  0.1666666853711689  0.8090702727272756   F   F   F
  0.2499999757021314  0.1666666853711689  0.8090702727272756   F   F   F
  0.1249999878510692  0.4166666573144155  0.8090702727272756   F   F   F
  0.3750000607446680  0.4166666573144155  0.8090702727272756   F   F   F
  0.5000000485957372  0.1666666853711689  0.8090702727272756   F   F   F
  0.7500000242978686  0.1666666853711689  0.8090702727272756   F   F   F
  0.6250000364467994  0.4166666573144155  0.8090702727272756   F   F   F
  0.8750000121489308  0.4166666573144155  0.8090702727272756   F   F   F
  0.2499999757021314  0.6666666292576551  0.8090702727272756   F   F   F
  0.5000000485957372  0.6666666292576551  0.8090702727272756   F   F   F
  0.3750000607446680  0.9166666012008946  0.8090702727272756   F   F   F
  0.6250000364467994  0.9166666012008946  0.8090702727272756   F   F   F
  0.7500000242978686  0.6666666292576551  0.8090702727272756   F   F   F
  0.0000000000000000  0.6666666292576551  0.8090702727272756   F   F   F
  0.8750000121489308  0.9166666012008946  0.8090702727272756   F   F   F
  0.1250000850425366  0.9166666012008946  0.8090702727272756   F   F   F
  0.6175060757628936  0.4070746095424618  0.0548810636806617   T   T   T
  0.6211624447148011  0.4085347568379901  0.1182104675227781   T   T   T
  0.5968719947098509  0.5086916911000886  0.1322498226678036   T   T   T
  0.7612657589417432  0.1512820876548467  0.1273917301515677   T   T   T
```

```
Cu_CHO_K
  1.0000000000000000
   10.2889689999999998    0.0000000000000000    0.0000000000000000
    0.0000000000000000    8.9105089999999993    0.0000000000000000
    0.0000000000000000    0.0000000000000000   22.0000000000000000
  Cu   C    O    H    K
   48    1    1    1    1
Selective dynamics
Direct
  0.0002868749668374 -0.0000253773620125 -0.0022842960603173   T   T   T
  0.1254223625666423  0.2504889962666406 -0.0020459554091887   T   T   T
  0.8759505019693484  0.2480328031036783 -0.0031388643250942   T   T   T
  0.6231448847420566  0.7523330348515277 -0.0033783422188004   T   T   T
  0.2496115626033737  0.0002500182442255 -0.0019301626619468   T   T   T
  0.3739622137759721  0.2506914665681584 -0.0021616087355025   T   T   T
  0.4988564676682082  0.0003088488456394 -0.0022034459078423   T   T   T
  0.7496463523409919  0.0003419642870282 -0.0027847579395785   T   T   T
  0.6233160088907007  0.2496691986507671 -0.0024631253592159   T   T   T
  0.4961692485643095  0.4999188441361798 -0.0062132490488251   T   T   T
  0.2496490637218173  0.5009419037665924 -0.0027040521153641   T   T   T
  0.3739974433140829  0.7510084267198563 -0.0038236767291434   T   T   T
  0.7477162433817938  0.5003003081950519  0.0064726908241901   T   T   T
  0.0028489846133069  0.5005009705056364 -0.0032005384904129   T   T   T
  0.8749077721669224  0.7506895175016789 -0.0022759440163936   T   T   T
  0.1254416742873523  0.7503135878924367 -0.0018480185512762   T   T   T
  0.1248457838013466  0.0839611413830354  0.9034498226922566   T   T   T
  0.3744654136983098  0.0835606407991146  0.9034561462023583   T   T   T
  0.2497020098655719  0.3336127106469086  0.9030528596645626   T   T   T
  0.5000828595991103  0.3323657208928507  0.9024415130216051   T   T   T
  0.6249775216881699  0.0835218232716426  0.9034024934088383   T   T   T
  0.8747957344456567  0.0832723994804773  0.9031231350737497   T   T   T
  0.7497764768954392  0.3355376388417528  0.9046182134839493   T   T   T
 -0.0003929531612269  0.3337621577432757  0.9033041296913098   T   T   T
  0.3739853795111341  0.5843708250378259  0.9017632878084497   T   T   T
  0.6270236004206324  0.5825312036228547  0.9035270734872707   T   T   T
  0.4999067611914865  0.8335510519928124  0.9027273620984717   T   T   T
  0.7502335540322246  0.8330684916386483  0.9031951255009424   T   T   T
  0.8731556249875183  0.5819152370344666  0.9046898847741739   T   T   T
  0.1245478592999267  0.5843958099659060  0.9028005214404583   T   T   T
 -0.0003840950795457  0.8330578284345078  0.9035941403288168   T   T   T
  0.2490922146018610  0.8344742274395900  0.9027561826867099   T   T   T
  0.0000000000000000  0.1666666853711689  0.8090702727272756   F   F   F
  0.2499999757021314  0.1666666853711689  0.8090702727272756   F   F   F
  0.1249999878510692  0.4166666573144155  0.8090702727272756   F   F   F
  0.3750000607446680  0.4166666573144155  0.8090702727272756   F   F   F
  0.5000000485957372  0.1666666853711689  0.8090702727272756   F   F   F
  0.7500000242978686  0.1666666853711689  0.8090702727272756   F   F   F
  0.6250000364467994  0.4166666573144155  0.8090702727272756   F   F   F
  0.8750000121489308  0.4166666573144155  0.8090702727272756   F   F   F
  0.2499999757021314  0.6666666292576551  0.8090702727272756   F   F   F
  0.5000000485957372  0.6666666292576551  0.8090702727272756   F   F   F
  0.3750000607446680  0.9166666012008946  0.8090702727272756   F   F   F
  0.6250000364467994  0.9166666012008946  0.8090702727272756   F   F   F
  0.7500000242978686  0.6666666292576551  0.8090702727272756   F   F   F
  0.0000000000000000  0.6666666292576551  0.8090702727272756   F   F   F
  0.8750000121489308  0.9166666012008946  0.8090702727272756   F   F   F
  0.1250000850425366  0.9166666012008946  0.8090702727272756   F   F   F
  0.7503547778073341  0.4919890473821117  0.0954605426032739   T   T   T
  0.6629386090880663  0.5207462870362362  0.1323434992587302   T   T   T
  0.8457634600076195  0.4556066898506050  0.1154141595095718   T   T   T
  0.4171451827648248  0.5859762392368580  0.1301347754561560   T   T   T
```

```
Cu111_C_Mg
  1.00000000000000
   10.2889409999999994    0.0000000000000000    0.0000000000000000
    0.0000000000000000    8.9104840000000003    0.0000000000000000
    0.0000000000000000    0.0000000000000000   22.0000000000000000
  Cu   C    Mg
   48    1    1
Selective dynamics
Direct
 -0.0005602839059893 -0.0000925964820752 -0.0024620185954524   T   T   T
  0.1211854841664306  0.2511667969042596 -0.0026874347019473   T   T   T
  0.8759192869791987  0.2512977873746354 -0.0026078218868831   T   T   T
  0.6267785695845527  0.7495423852431969 -0.0023244601474242   T   T   T
  0.2464332631178779 -0.0062562618963981 -0.0031396915343941   T   T   T
  0.3629591136272467  0.2420011731032649 -0.0055099099174702   T   T   T
  0.5001700415706304 -0.0045011274331033  0.0043345443214613   T   T   T
  0.7525209944911982 -0.0016747297725898 -0.0023938293049553   T   T   T
  0.6297664898345661  0.2536890392390604  0.0028606170746265   T   T   T
  0.5030493440844640  0.5070624556880909 -0.0059091848116061   T   T   T
  0.2531336098239025  0.4988142197253922  0.0004878460239251   T   T   T
  0.3748983760824511  0.7462056014164089 -0.0014448992476345   T   T   T
  0.7518446203794836  0.5025774389189047 -0.0030171291000994   T   T   T
  0.0007436417721490  0.5004196371815878 -0.0021678919292613   T   T   T
  0.8763165950319001  0.7503508471226574 -0.0023670820619615   T   T   T
  0.1243958436177180  0.7478742577003511 -0.0018563182440651   T   T   T
  0.1252042899523626  0.0850239635557202  0.9033432003841375   T   T   T
  0.3748717006949988  0.0798897978919002  0.9023114946090425   T   T   T
  0.2489074438949481  0.3343543826486348  0.9024015863340256   T   T   T
  0.5000311521162263  0.3315817440075109  0.9032702229845112   T   T   T
  0.6242983535440834  0.0831642674998248  0.9049049974400113   T   T   T
  0.8746776956928248  0.0847515719728840  0.9032526393413366   T   T   T
  0.7489124515871146  0.3326038475573286  0.9040441718645504   T   T   T
 -0.0009418425605616  0.3355769379892671  0.9028982048446081   T   T   T
  0.3742273816064096  0.5824918543561609  0.9022710252577621   T   T   T
  0.6262445581267492  0.5833266144497552  0.9019440440884848   T   T   T
  0.4999990598556057  0.8344856895497764  0.9050525066969255   T   T   T
  0.7502155476763054  0.8340428021266779  0.9033821937897808   T   T   T
  0.8759858857914181  0.5845660477990728  0.9030518972193635   T   T   T
  0.1256156818102698  0.5836798192882579  0.9040025062418405   T   T   T
  0.0004537531069245  0.8347341288484015  0.9033695645819545   T   T   T
  0.2500579366988330  0.8319470834224595  0.9034326728135559   T   T   T
  0.0000000000000000  0.1666667040757801  0.8090707727272743   F   F   F
  0.2499999757020674  0.1666667040757801  0.8090707727272743   F   F   F
  0.1250000364468988  0.4166667040757801  0.8090707727272743   F   F   F
  0.3750000121489663  0.4166667040757801  0.8090707727272743   F   F   F
  0.5000000485958651  0.1666667040757801  0.8090707727272743   F   F   F
  0.7500000242979326  0.1666667040757801  0.8090707727272743   F   F   F
  0.6249999878510337  0.4166667040757801  0.8090707727272743   F   F   F
  0.8749999635531012  0.4166667040757801  0.8090707727272743   F   F   F
  0.2499999757020674  0.6666667040757801  0.8090707727272743   F   F   F
  0.5000000485958651  0.6666667040757801  0.8090707727272743   F   F   F
  0.3750000121489663  0.9166667040757801  0.8090707727272743   F   F   F
  0.6249999878510337  0.9166667040757801  0.8090707727272743   F   F   F
  0.7500000242979326  0.6666667040757801  0.8090707727272743   F   F   F
  0.0000000000000000  0.6666667040757801  0.8090707727272743   F   F   F
  0.8749999635531012  0.9166667040757801  0.8090707727272743   F   F   F
  0.1250000364468988  0.9166667040757801  0.8090707727272743   F   F   F
  0.4925545067307568  0.1740940571749170  0.0514475253171677   T   T   T
  0.4279621811640409  0.3819117793437176  0.0913621249045310   T   T   T
```

```
Cu111_CH_Mg
  1.00000000000000
   10.2889409999999994    0.0000000000000000    0.0000000000000000
    0.0000000000000000    8.9104840000000003    0.0000000000000000
    0.0000000000000000    0.0000000000000000   22.0000000000000000
  Cu   C    H    Mg
   48    1    1    1
Selective dynamics
Direct
 -0.0005992328484014 -0.0000594628470185 -0.0002639371343128   T   T   T
  0.1261890066636037  0.2460928609901638 -0.0013955163764424   T   T   T
  0.8763125827694639  0.2500570811435137 -0.0020613082250881   T   T   T
  0.6258517112670245  0.7493943610227902 -0.0030608053529465   T   T   T
  0.2411172628299124 -0.0117037315729312 -0.0089087780603266   T   T   T
  0.3781346761985195  0.2653205249807395 -0.0021710433529177   T   T   T
  0.4981119470197091 -0.0058500091693918  0.0041270518810071   T   T   T
  0.7515778078214762 -0.0016983929940654 -0.0023869848199613   T   T   T
  0.6276849160889668  0.2498843173197034  0.0025139022119566   T   T   T
  0.5027388964684371  0.5051789535257922 -0.0032604172732284   T   T   T
  0.2476153101742983  0.5013222090563964 -0.0026963418871884   T   T   T
  0.3732561963367484  0.7487663364256401 -0.0034319140012435   T   T   T
  0.7518203442342847  0.5001985422013229 -0.0028339727805115   T   T   T
 -0.0004245642407893  0.4979398218920584 -0.0022772113053036   T   T   T
  0.8749741518525682  0.7493621221710336 -0.0023405275434028   T   T   T
  0.1221701626561641  0.7456468730219006 -0.0019726994176461   T   T   T
  0.1228518898995749  0.0846846296452399  0.9015176707178896   T   T   T
  0.3748391649462116  0.0837494970002445  0.9032858443743633   T   T   T
  0.2515203569195102  0.3312836109600005  0.9037845914908906   T   T   T
  0.5016322127568976  0.3332473199695995  0.9025209967727098   T   T   T
  0.6238444304093159  0.0832743112573070  0.9045523279028981   T   T   T
  0.8746756099681168  0.0836614050182471  0.9039765536753872   T   T   T
  0.7487492953779360  0.3315620616809921  0.9046375867669234   T   T   T
  0.0000213284617820  0.3336807312908049  0.9032544173795208   T   T   T
  0.3754378202339601  0.5808782678958445  0.9031386720726491   T   T   T
  0.6259411127770973  0.5832590278991104  0.9028736460973564   T   T   T
  0.4995568445825604  0.8368365287700428  0.9047056093346290   T   T   T
  0.7496298301132137  0.8340385292709257  0.9033408564968501   T   T   T
  0.8753700374050812  0.5838453473327121  0.9030092528203264   T   T   T
  0.1243330095858817  0.5823794081816888  0.9031135447740839   T   T   T
  0.0002699838583816  0.8345409005135821  0.9038308255117269   T   T   T
  0.2498476018264190  0.8293842408631903  0.9010381644379065   T   T   T
  0.0000000000000000  0.1666667040757801  0.8090707727272743   F   F   F
  0.2499999757020674  0.1666667040757801  0.8090707727272743   F   F   F
  0.1250000364468988  0.4166667040757801  0.8090707727272743   F   F   F
  0.3750000121489663  0.4166667040757801  0.8090707727272743   F   F   F
  0.5000000485958651  0.1666667040757801  0.8090707727272743   F   F   F
  0.7500000242979326  0.1666667040757801  0.8090707727272743   F   F   F
  0.6249999878510337  0.4166667040757801  0.8090707727272743   F   F   F
  0.8749999635531012  0.4166667040757801  0.8090707727272743   F   F   F
  0.2499999757020674  0.6666667040757801  0.8090707727272743   F   F   F
  0.5000000485958651  0.6666667040757801  0.8090707727272743   F   F   F
  0.3750000121489663  0.9166667040757801  0.8090707727272743   F   F   F
  0.6249999878510337  0.9166667040757801  0.8090707727272743   F   F   F
  0.7500000242979326  0.6666667040757801  0.8090707727272743   F   F   F
  0.0000000000000000  0.6666667040757801  0.8090707727272743   F   F   F
  0.8749999635531012  0.9166667040757801  0.8090707727272743   F   F   F
  0.1250000364468988  0.9166667040757801  0.8090707727272743   F   F   F
  0.4918696917020829  0.1687011121877919  0.0585531713143166   T   T   T
  0.5382796418162142  0.1848734628685476  0.1034631526337826   T   T   T
  0.2867272515252815  0.1240789322772834  0.0872102680565210   T   T   T
```

```
Cu111_CH2_Mg
  1.00000000000000
   10.2889409999999994    0.0000000000000000    0.0000000000000000
    0.0000000000000000    8.9104840000000003    0.0000000000000000
    0.0000000000000000    0.0000000000000000   22.0000000000000000
  Cu   C    H    Mg
   48    1    2    1
Selective dynamics
Direct
  0.0001049910537319  0.0007743242368565 -0.0015945154967866   T   T   T
  0.1256991417470114  0.2497501901872672 -0.0028886840699417   T   T   T
  0.8763990817165626  0.2511366392908044 -0.0022894939527629   T   T   T
  0.6261490222491373  0.7512563214747545 -0.0022323404334084   T   T   T
  0.2433911553403560 -0.0028845926711517 -0.0064955780957342   T   T   T
  0.3775090915427993  0.2530219017658385 -0.0005297393518174   T   T   T
  0.5055954484963562  0.0036796896562820 -0.0028401012726639   T   T   T
  0.7557779320618602 -0.0010917050874086 -0.0039514008958780   T   T   T
  0.6253861712613715  0.2533905964888163  0.0030067851197886   T   T   T
  0.5017970651111474  0.5048462786580464 -0.0030742019854342   T   T   T
  0.2509329211981802  0.5000144091342883 -0.0024346455120333   T   T   T
  0.3743423146300950  0.7492440946632372 -0.0025213517627182   T   T   T
  0.7515789763689650  0.5015697423850716 -0.0025096852938914   T   T   T
  0.0012760134756217  0.5006940483912654 -0.0021558969330576   T   T   T
  0.8768174057312644  0.7494016823321984 -0.0023405170704232   T   T   T
  0.1268086009091199  0.7492970119491424 -0.0001824059509456   T   T   T
  0.1235653212496291  0.0843313836107032  0.9021693611187457   T   T   T
  0.3748688698405603  0.0835972866093906  0.9026926357389536   T   T   T
  0.2526285422636849  0.3316231848921085  0.9045050272819849   T   T   T
  0.5014959421667720  0.3333366695716161  0.9034944573689773   T   T   T
  0.6266601561187093  0.0845560562602813  0.9029779313562624   T   T   T
  0.8761443076657530  0.0844966108017220  0.9030177174721519   T   T   T
  0.7486092107922711  0.3323003379518619  0.9050068652610938   T   T   T
  0.0003248080649701  0.3350408546127393  0.9030721712869401   T   T   T
  0.3760408187497267  0.5827374213838462  0.9030050266296979   T   T   T
  0.6266573741101340  0.5840328333254495  0.9031096626230732   T   T   T
  0.5006345751539818  0.8357686352797139  0.9038441613055068   T   T   T
  0.7506712572323163  0.8332758582024945  0.9033662870813481   T   T   T
  0.8759791635897696  0.5838231319011459  0.9031433466358063   T   T   T
  0.1259500200477121  0.5840494922922780  0.9037494299805181   T   T   T
  0.0014789391618116  0.8339278153353669  0.9039472664971961   T   T   T
  0.2506513446990368  0.8316479686468633  0.9017384245074945   T   T   T
  0.0000000000000000  0.1666667040757801  0.8090707727272743   F   F   F
  0.2499999757020674  0.1666667040757801  0.8090707727272743   F   F   F
  0.1250000364468988  0.4166667040757801  0.8090707727272743   F   F   F
  0.3750000121489663  0.4166667040757801  0.8090707727272743   F   F   F
  0.5000000485958651  0.1666667040757801  0.8090707727272743   F   F   F
  0.7500000242979326  0.1666667040757801  0.8090707727272743   F   F   F
  0.6249999878510337  0.4166667040757801  0.8090707727272743   F   F   F
  0.8749999635531012  0.4166667040757801  0.8090707727272743   F   F   F
  0.2499999757020674  0.6666667040757801  0.8090707727272743   F   F   F
  0.5000000485958651  0.6666667040757801  0.8090707727272743   F   F   F
  0.3750000121489663  0.9166667040757801  0.8090707727272743   F   F   F
  0.6249999878510337  0.9166667040757801  0.8090707727272743   F   F   F
  0.7500000242979326  0.6666667040757801  0.8090707727272743   F   F   F
  0.0000000000000000  0.6666667040757801  0.8090707727272743   F   F   F
  0.8749999635531012  0.9166667040757801  0.8090707727272743   F   F   F
  0.1250000364468988  0.9166667040757801  0.8090707727272743   F   F   F
  0.4953867497912626  0.1472883539942303  0.0708952995733323   T   T   T
  0.5840036585653253  0.1610593628455502  0.0988069078948462   T   T   T
  0.4196457451113988  0.2194109629656823  0.0927521719953134   T   T   T
  0.3805848231377865 -0.0642026530269646  0.0897914442700555   T   T   T
```

```
Cu111_CH3_Mg
  1.00000000000000
   10.2889409999999994    0.0000000000000000    0.0000000000000000
    0.0000000000000000    8.9104840000000003    0.0000000000000000
    0.0000000000000000    0.0000000000000000   22.0000000000000000
  Cu   C    H    Mg
   48    1    3    1
Selective dynamics
Direct
 -0.0027359487452240 -0.0043754568635416 -0.0068173600985616   T   T   T
  0.1287835023952933  0.2537792616343713 -0.0039967693494748   T   T   T
  0.8800633799718048  0.2484218250668393 -0.0015072256950890   T   T   T
  0.6274696162480992  0.7514118954014301 -0.0030769620414036   T   T   T
  0.2485998417345680 -0.0004376757745358 -0.0033349313287874   T   T   T
  0.3781905000852883  0.2510659351408259  0.0018287226168584   T   T   T
  0.5020349084627646  0.0030550072592761  0.0005709533208134   T   T   T
  0.7546225637519232 -0.0020831282217095 -0.0041386040079691   T   T   T
  0.6274066702206462  0.2500894803056429  0.0002568002766875   T   T   T
  0.5020221433483222  0.5054569655795110 -0.0041957632970312   T   T   T
  0.2531447744894887  0.5025741232509059 -0.0027690155266182   T   T   T
```

```
0.3762812150555111 0.7530056843101038 -0.0029277602653818 T T T
0.7520620408026845 0.4997150428967186 -0.0031774094734525 T T T
0.0017039897781349 0.5007368180444111 -0.0027305314549287 T T T
0.8759215580073481 0.7475509626335272 -0.0025830448060246 T T T
0.1259402801694427 0.7508407629899486 -0.0018917149547777 T T T
0.1256428448749478 0.0839056587070564 0.9011586237319609 T T T
0.3762185161567066 0.0848358393580458 0.9040872902819473 T T T
0.2547852118589440 0.3329356952417259 0.9043505878503966 T T T
0.5013238818833720 0.3339892019685560 0.9037032477989753 T T T
0.6257984135845465 0.0844036569715676 0.9034179557123373 T T T
0.8749974002002158 0.0851903525480299 0.9018895323105166 T T T
0.7491702340595650 0.3317665987033355 0.9049452845155873 T T T
0.0010755975238052 0.3340812875881106 0.9026140977455062 T T T
0.3762909916471308 0.5851316900788723 0.9026210712388850 T T T
0.6264630362148895 0.5843794269449691 0.9023867537967466 T T T
0.5010274730509257 0.8378556630868831 0.9046718675659219 T T T
0.7509324567207318 0.8334745078503879 0.9028350553931650 T T T
0.8759270274517140 0.5828061508646292 0.9028116206524984 T T T
0.1265990001591007 0.5831665884258230 0.9033396267305831 T T T
0.0008874440526520 0.8312821520500323 0.9020658282492159 T T T
0.2508724262295093 0.8338654783341108 0.9030739885460232 T T T
0.0000000000000000 0.1666667040757801 0.8090707727272743 F F F
0.2499999757020674 0.1666667040757801 0.8090707727272743 F F F
0.1250000364468988 0.4166667040757801 0.8090707727272743 F F F
0.3750000121489663 0.4166667040757801 0.8090707727272743 F F F
0.5000000485958651 0.1666667040757801 0.8090707727272743 F F F
0.7500000242979326 0.1666667040757801 0.8090707727272743 F F F
0.6249999878510337 0.4166667040757801 0.8090707727272743 F F F
0.8749999635531012 0.4166667040757801 0.8090707727272743 F F F
0.2499999757020674 0.6666667040757801 0.8090707727272743 F F F
0.5000000485958651 0.6666667040757801 0.8090707727272743 F F F
0.3750000121489663 0.9166667040757801 0.8090707727272743 F F F
0.6249999878510337 0.9166667040757801 0.8090707727272743 F F F
0.7500000242979326 0.6666667040757801 0.8090707727272743 F F F
0.0000000000000000 0.6666667040757801 0.8090707727272743 F F F
0.8749999635531012 0.9166667040757801 0.8090707727272743 F F F
0.1250000364468988 0.9166667040757801 0.8090707727272743 F F F
0.5181361523422484 0.1665040798274534 0.0779118509221307 T T T
0.4360683667051162 0.2283710735455537 0.0985199334164623 T T T
0.6082903417696488 0.2182008734789637 0.0960209298949538 T T T
0.5154894633201903 0.0515982194477249 0.0970399984914697 T T T
0.0965485187982339 0.0835243387599714 0.0895825478056277 T T T


Cu_CO_Mg
  1.00000000000000
   10.2889689999999998   0.0000000000000000   0.0000000000000000
    0.0000000000000000   8.9105089999999993   0.0000000000000000
    0.0000000000000000   0.0000000000000000  22.0000000000000000
  Cu   C   O   Mg
   48   1   1   1
Selective dynamics
Direct
-0.0038746508618628 -0.0018359747006447 -0.0024365156405026 T T T
 0.1218806067520855 0.2498929169964806 0.0047126220846522 T T T
 0.8739211279124360 0.2496344614388250 -0.0013590890818825 T T T
 0.6243110896272518 0.7475211271400286 -0.0021766528630801 T T T
 0.2496777015511665 -0.0019441405343107 0.0007382910761242 T T T
 0.3743201732864977 0.2537740772790741 -0.0061186664036410 T T T
 0.5022535401452811 -0.0054604748426429 -0.0055889853532170 T T T
 0.7477901508187683 0.0000709525569215 -0.0004483853574719 T T T
 0.6252320207894244 0.2493202772541479 -0.0014028114438569 T T T
 0.4999766721563724 0.4986980426625119 -0.0026502223980670 T T T
 0.2477681840527368 0.5022157847942003 -0.0040035113429717 T T T
 0.3745713636851943 0.7484728593640131 -0.0033296237965227 T T T
 0.7496193032086379 0.4992287063369192 -0.0019503845759646 T T T
-0.0016105100434591 0.4998644591526408 -0.0024032734475786 T T T
 0.8735508822947615 0.7490601685689108 -0.0025178654564373 T T T
 0.1233179280650308 0.7501373665428887 -0.0030917664071586 T T T
 0.1245189090915078 0.0833856128325387 0.9036697573487508 T T T
 0.3738963804129490 0.0820170765770433 0.9016968994426040 T T T
 0.2473226189640430 0.3330310398827157 0.9021464975649892 T T T
 0.5003305969776412 0.3328420950344906 0.9022138225579345 T T T
 0.6252907266983109 0.0842222306712151 0.9020279202259147 T T T
 0.8740324654883133 0.0839340400285103 0.9040473682152294 T T T
 0.7503173351909240 0.3334943413715156 0.9036535001497555 T T T
 0.0009284464638762 0.3314945587117562 0.9051321723876350 T T T
 0.3745694993731226 0.5821103702299093 0.9030820926002715 T T T
 0.6256088988118819 0.5827268246551160 0.9033977022540870 T T T
 0.5005123502943930 0.8312868790666114 0.9023035065203859 T T T
 0.7492591467632733 0.8346182789881318 0.9039991156078279 T T T
 0.8743961982220961 0.5831456036907031 0.9032252867733024 T T T
 0.1233092317064660 0.5838388645334937 0.9029003141955927 T T T
-0.0011591622455658 0.8339158097779671 0.9033117957186156 T T T
 0.2491837579490601 0.8347280837809987 0.9041173559675558 T T T
 0.0000000000000000 0.1666666853711689 0.8090702727272756 F F F
 0.2499999757021314 0.1666666853711689 0.8090702727272756 F F F
 0.1249999878510692 0.4166666573144155 0.8090702727272756 F F F
 0.3750000607446680 0.4166666573144155 0.8090702727272756 F F F
 0.5000000485957372 0.1666666853711689 0.8090702727272756 F F F
 0.7500000242978686 0.1666666853711689 0.8090702727272756 F F F
 0.6250000364467994 0.4166666573144155 0.8090702727272756 F F F
 0.8750000121489308 0.4166666573144155 0.8090702727272756 F F F
 0.2499999757021314 0.6666666292576551 0.8090702727272756 F F F
 0.5000000485957372 0.6666666292576551 0.8090702727272756 F F F
 0.3750000607446680 0.9166666012008946 0.8090702727272756 F F F
 0.6250000364467994 0.9166666012008946 0.8090702727272756 F F F
 0.7500000242978686 0.6666666292576551 0.8090702727272756 F F F
 0.0000000000000000 0.6666666292576551 0.8090702727272756 F F F
 0.8750000121489308 0.9166666012008946 0.8090702727272756 F F F
 0.1250000850425366 0.9166666012008946 0.8090702727272756 F F F
 0.2550365222576667 0.1701794143601022 0.0595528076832894 T T T
 0.2916906057421180 0.1686316250047930 0.1138403558748413 T T T
 0.4894087664768157 0.1530689940120651 0.0917060383225011 T T T


Cu_COOH_Mg
  1.0000000000000000
   10.2889689999999998   0.0000000000000000   0.0000000000000000
    0.0000000000000000   8.9105089999999993   0.0000000000000000
    0.0000000000000000   0.0000000000000000  22.0000000000000000
  Cu   C   O   H   Mg
   48   1   2   1   1
Selective dynamics
Direct
-0.0000315460080764 0.0004408356061998 0.9976251518672620 T T T
 0.1247881416608736 0.2512559961895229 0.9988281219739156 T T T
 0.8764869531264761 0.2506292863266855 0.9974790973889256 T T T
 0.6253242065480895 0.7513209273644572 0.9970020977649161 T T T
 0.2486801529215339 -0.0011879654549667 0.9976217605427689 T T T
 0.3692530164408027 0.2463395225009043 0.9925871224761599 T T T
 0.4973068197369423 -0.0013569562639154 0.9969249634631677 T T T
 0.7508582608759375 -0.0012975943392705 0.9971854449244649 T T T
 0.6220483101771732 0.2514080148165916 0.0104316497754173 T T T
 0.5004902581191720 0.5068908849409758 0.9920286933380557 T T T
 0.2499624803040837 0.4994096318074240 0.9979039270967140 T T T
 0.3736314367723332 0.7482658215627346 -0.0014359358964547 T T T
 0.7503610060513501 0.5026977487778606 0.9971220768399814 T T T
-0.0003807698358284 0.5007823331596151 0.9977328265614789 T T T
 0.8754352459078123 0.7504003983964579 0.9974161856596885 T T T
 0.1240705911070371 0.7497995254718560 0.9978489180788263 T T T
 0.1247543989218118 0.0845026049239007 0.9036801212188403 T T T
 0.3744815934342275 0.0814907895322770 0.9019389388241801 T T T
 0.2481384936033693 0.3347068874372813 0.9013286301321616 T T T
 0.5021874245513360 0.3313490331883429 0.9032495968636934 T T T
 0.6245338393572569 0.0865835998844704 0.9054764048299839 T T T
 0.8744711791663820 0.0845955585865119 0.9034553674147108 T T T
 0.7475181101351024 0.3321967210951943 0.9056571507627169 T T T
-0.0006976880464152 0.3340291065451699 0.9037253691523963 T T T
 0.3732002431153610 0.5840144436051403 0.9010633804958045 T T T
 0.6266983736175402 0.5840527687824726 0.9017941628923145 T T T
 0.4996541800003688 0.8324462384756478 0.9034553762482354 T T T
 0.7499913780358819 0.8347181112304949 0.9031651693253181 T T T
 0.8750566485953077 0.5836410437601836 0.9031769549778952 T T T
 0.1245612463303391 0.5839657554969457 0.9033804103696723 T T T
-0.0003297580802004 0.8343264320194836 0.9032295840954030 T T T
 0.2494869275199552 0.8331161808824780 0.9035390559995937 T T T
 0.0000000000000000 0.1666666853711689 0.8090702727272756 F F F
 0.2499999757021314 0.1666666853711689 0.8090702727272756 F F F
 0.1249999878510692 0.4166666573144155 0.8090702727272756 F F F
 0.3750000607446680 0.4166666573144155 0.8090702727272756 F F F
 0.5000000485957372 0.1666666853711689 0.8090702727272756 F F F
 0.7500000242978686 0.1666666853711689 0.8090702727272756 F F F
 0.6250000364467994 0.4166666573144155 0.8090702727272756 F F F
 0.8750000121489308 0.4166666573144155 0.8090702727272756 F F F
 0.2499999757021314 0.6666666292576551 0.8090702727272756 F F F
 0.5000000485957372 0.6666666292576551 0.8090702727272756 F F F
 0.3750000607446680 0.9166666012008946 0.8090702727272756 F F F
 0.6250000364467994 0.9166666012008946 0.8090702727272756 F F F
 0.7500000242978686 0.6666666292576551 0.8090702727272756 F F F
 0.0000000000000000 0.6666666292576551 0.8090702727272756 F F F
 0.8750000121489308 0.9166666012008946 0.8090702727272756 F F F
 0.1250000850425366 0.9166666012008946 0.8090702727272756 F F F
 0.6211839849709712 0.2534305220410334 0.1009296619362232 T T T
 0.5347932077630465 0.3112918310219221 0.1356843170085101 T T T
 0.7203926564992706 0.1878013027956250 0.1305878640280347 T T T
 0.7044351687876259 0.1995119322503994 0.1747370449294294 T T T
 0.3947281453411251 0.4083813846344631 0.0888459210052478 T T T


Cu_COH_Mg
  1.0000000000000000
   10.2889689999999998   0.0000000000000000   0.0000000000000000
    0.0000000000000000   8.9105089999999993   0.0000000000000000
    0.0000000000000000   0.0000000000000000  22.0000000000000000
  Cu   C   O   H   Mg
   48   1   1   1   1
Selective dynamics
Direct
-0.0013464182069625 0.0016208904889353 -0.0001940361092660 T T T
 0.1249434946606944 0.2517457586212582 -0.0016090208435768 T T T
 0.8800273514820479 0.2500282246234092 -0.0047928638743210 T T T
 0.6243282606543944 0.7519617557544593 -0.0027564913144569 T T T
 0.2483697295108802 0.0015912524063691 -0.0020506871961602 T T T
 0.3713894541278708 0.2509787137601185 -0.0025321722967073 T T T
 0.4988803953032631 -0.0027489430409599 -0.0022886494585096 T T T
 0.7510640853174894 -0.0029973463526027 -0.0017039691315046 T T T
 0.6192740950435910 0.2411048092578119 -0.0047959649039403 T T T
 0.4946444816932395 0.5063747502688415 0.0049990946990790 T T T
 0.2478281443534229 0.5024274263167232 -0.0019309128751972 T T T
 0.3722220222269155 0.7526158121632702 -0.0023342681154911 T T T
 0.7525012562971396 0.5016901274462068 0.0019685070887408 T T T
 0.0009096480754042 0.5011179961052329 -0.0020920091034919 T T T
 0.8757498263193280 0.7501481696834691 -0.0024101224135337 T T T
 0.1240659676280884 0.7521506900101903 -0.0017959030737467 T T T
 0.1240546927461209 0.0847155015168587 0.9041113215226262 T T T
 0.3748459669788803 0.0838319398452430 0.9038057384587715 T T T
 0.2493693461309388 0.3347843441042186 0.9034371206713769 T T T
 0.4988492518674412 0.3348273423802356 0.9026063416376504 T T T
 0.6247070312776128 0.0830010123126032 0.9025252289152460 T T T
 0.8744855554141556 0.0826743373323093 0.9024061028814419 T T T
 0.7492298535986336 0.3334727448408992 0.9023422244992279 T T T
 0.0006820870342496 0.3347310541777532 0.9025820516186814 T T T
 0.3765387295208909 0.5829447018417692 0.9050553934141096 T T T
 0.6241778609695371 0.5821294473251206 0.9037954258824328 T T T
 0.4993091420187037 0.8324604127174472 0.9034492335499843 T T T
 0.7499581477589965 0.8318602593041780 0.9035879905540718 T T T
 0.8733632873360933 0.5820231861216739 0.9044305550293260 T T T
 0.1248469785283164 0.5859137525731734 0.9032523985575761 T T T
-0.0001441728709151 0.8351379871347662 0.9038447698453069 T T T
 0.2492499205801024 0.8346734796908566 0.9031910176002187 T T T
 0.0000000000000000 0.1666666853711689 0.8090702727272756 F F F
 0.2499999757021314 0.1666666853711689 0.8090702727272756 F F F
 0.1249999878510692 0.4166666573144155 0.8090702727272756 F F F
 0.3750000607446680 0.4166666573144155 0.8090702727272756 F F F
 0.5000000485957372 0.1666666853711689 0.8090702727272756 F F F
 0.7500000242978686 0.1666666853711689 0.8090702727272756 F F F
 0.6250000364467994 0.4166666573144155 0.8090702727272756 F F F
 0.8750000121489308 0.4166666573144155 0.8090702727272756 F F F
 0.2499999757021314 0.6666666292576551 0.8090702727272756 F F F
 0.5000000485957372 0.6666666292576551 0.8090702727272756 F F F
 0.3750000607446680 0.9166666012008946 0.8090702727272756 F F F
 0.6250000364467994 0.9166666012008946 0.8090702727272756 F F F
 0.7500000242978686 0.6666666292576551 0.8090702727272756 F F F
 0.0000000000000000 0.6666666292576551 0.8090702727272756 F F F
 0.8750000121489308 0.9166666012008946 0.8090702727272756 F F F
 0.1250000850425366 0.9166666012008946 0.8090702727272756 F F F
 0.6226263931855686 0.4019289036197151 0.0530481758693589 T T T
 0.6391975256037250 0.3732372531136766 0.1174700847530995 T T T
 0.6478211100520350 0.4677371053609289 0.1397612037442534 T T T
 0.7490212062334625 0.1845278071302773 0.0911266282019556 T T T


Cu_CHO_Mg
  1.0000000000000000
   10.2889689999999998   0.0000000000000000   0.0000000000000000
    0.0000000000000000   8.9105089999999993   0.0000000000000000
    0.0000000000000000   0.0000000000000000  22.0000000000000000
  Cu   C   O   H   Mg
   48   1   1   1   1
Selective dynamics
Direct
-0.0013647991318323 -0.0025026888646421 -0.0024754687004474 T T T
 0.1227460236851675 0.2478039432341580 -0.0022726815883302 T T T
 0.8739118715110543 0.2458835248402612 -0.0031274944550816 T T T
 0.6234018099107178 0.7557889378122886 -0.0079040992074209 T T T
 0.2475497597119092 -0.0032225286261199 -0.0019265274444211 T T T
 0.3714922041308042 0.2464501278394008 -0.0023937650053641 T T T
 0.4969753781803276 -0.0026864401950329 -0.0018185551570536 T T T
 0.7487071170576614 -0.0008311170474471 -0.0029454732807092 T T T
 0.6210750393581663 0.2475900724899090 -0.0025033711058522 T T T
 0.4924963676548966 0.4940995500894734 -0.0071593063490188 T T T
 0.2476392394980990 0.4983254286110591 -0.0011226840851201 T T T
 0.3749022923215370 0.7459731137061913 -0.0003204989097668 T T T
 0.7454435871937937 0.4992649304744607 0.0089766397148330 T T T
-0.0004065665710682 0.4984199975450757 -0.0027787624240008 T T T
 0.8735424883508702 0.7490325239375886 -0.0022084369313660 T T T
 0.1233838902300033 0.7478597794036209 -0.0018872463148209 T T T
 0.1240259253891158 0.0824747015514245 0.9033600393570346 T T T
 0.3732428557581615 0.0818513036749538 0.9036468470871755 T T T
 0.2485065657139481 0.3329712432192226 0.9037054732639330 T T T
 0.4983669963325497 0.3302548059911409 0.9021193472343462 T T T
 0.6237258567122842 0.0816409023806985 0.9035605358671946 T T T
 0.8742017986231275 0.0828042180328439 0.9030566788847246 T T T
 0.7483818091943752 0.3353120626167047 0.9051995253177358 T T T
-0.0017550634102158 0.3328968960293006 0.9034146514251391 T T T
 0.3722114302183427 0.5834248403815046 0.9016631385166588 T T T
 0.6255304476187534 0.5806067049346104 0.9029613736084217 T T T
 0.4969030583008240 0.8328111825370724 0.9013903868596843 T T T
 0.7506622163402676 0.8328231449561611 0.9020542685554944 T T T
 0.8714741370588196 0.5811844006415949 0.9050971086890292 T T T
 0.1233557212405706 0.5823350078712718 0.9035445742186186 T T T
-0.0007161161892514 0.8316193163990285 0.9034332908547204 T T T
 0.2489705463552401 0.8318400653557518 0.9039565698700754 T T T
 0.0000000000000000 0.1666666853711689 0.8090702727272756 F F F
 0.2499999757021314 0.1666666853711689 0.8090702727272756 F F F
 0.1249999878510692 0.4166666573144155 0.8090702727272756 F F F
 0.3750000607446680 0.4166666573144155 0.8090702727272756 F F F
 0.5000000485957372 0.1666666853711689 0.8090702727272756 F F F
 0.7500000242978686 0.1666666853711689 0.8090702727272756 F F F
 0.6250000364467994 0.4166666573144155 0.8090702727272756 F F F
 0.8750000121489308 0.4166666573144155 0.8090702727272756 F F F
 0.2499999757021314 0.6666666292576551 0.8090702727272756 F F F
 0.5000000485957372 0.6666666292576551 0.8090702727272756 F F F
 0.3750000607446680 0.9166666012008946 0.8090702727272756 F F F
 0.6250000364467994 0.9166666012008946 0.8090702727272756 F F F
 0.7500000242978686 0.6666666292576551 0.8090702727272756 F F F
 0.0000000000000000 0.6666666292576551 0.8090702727272756 F F F
 0.8750000121489308 0.9166666012008946 0.8090702727272756 F F F
 0.1250000850425366 0.9166666012008946 0.8090702727272756 F F F
 0.7542770041476126 0.4946495844837308 0.0975223159643954 T T T
 0.6762765252749156 0.5517113151566218 0.1362112575100374 T T T
 0.8371736681814825 0.4342371438394870 0.1192218786642245 T T T
 0.5323042926703515 0.6453513550044743 0.0895206113960944 T T T
```
